# Supplementary material for: Caerulomycin and collismycin antibiotics share a trans-acting flavoprotein-dependent assembly line for 2,2’-bipyridine formation
Source: Nat Commun. 2021 May 25;12:3124. doi: 10.1038/s41467-021-23475-4 (PMC8149447; doi:10.1038/s41467-021-23475-4)
Supplement: Supplementary file 1 — Supplemetry Information [file 41467_2021_23475_MOESM1_ESM.pdf]

Supplementary Information

Caerulomycin and Collismycin Antibiotics Share a *trans*-Acting Flavoprotein-Dependent Assembly Line for 2,2'-Bipyridine Formation

Bo Pang,[1,#] Rijing Liao,[2,#] Zhijun Tang,[1#] Shengjie Guo,[1] Zhuhua Wu,[1] and Wen Liu[1,3,*]

[1] State Key Laboratory of Bioorganic and Natural Products Chemistry, Center for Excellence in Molecular Synthesis, Shanghai Institute of Organic Chemistry, University of Chinese Academy of Sciences, 345 Lingling Road, Shanghai 200032, China;

[2] Shanghai Institute of Precision Medicine, Ninth People's Hospital, Shanghai Jiao Tong University School of Medicine, Shanghai 200125, China;

[3] Huzhou Center of Bio-Synthetic Innovation, 1366 Hongfeng Road, Huzhou 313000, China;

* To whom correspondence should be addressed: Shanghai Institute of Organic Chemistry, Chinese Academy of Sciences, 345 Lingling Rd., Shanghai 200032, China. Wen Liu, Email: wliu@sioc.ac.cn, Tel: 86-21-54925111, Fax: 86-21-64166128

[#] These authors equally contribute to this work.

SUPPLIMENTARY METHODS

General materials and methods. Biochemicals and media were purchased from Sinopharm Chemical Reagent Co., Ltd. (China), Oxoid Ltd. (U.K.) or Sigma-Aldrich Co. LLC. (USA) unless otherwise stated. Restriction endonucleases were purchased from Thermo Fisher Scientific Co. Ltd. (USA). Chemical reagents were purchased from standard commercial sources. The bacterial strains, plasmids and primers used in this study are summarized in **Supplementary Tables 1**, **2**, and **3**, respectively.

DNA isolation and manipulation in *E. coli* or actinobacteria were carried out according to standard methods[1,2]. PCR amplifications were carried out on an Applied Biosystems Veriti Thermal Cycler (Thermo Fisher Scientific Inc., USA) using either Taq DNA polymerase (Vazyme Biotech Co. Ltd, China) for routine verification or PrimeSTAR HS DNA polymerase (Takara Biotechnology Co., Ltd. Japan) for high fidelity amplification. Primer synthesis was performed at Shanghai Sangon Biotech Co. Ltd. (China). DNA sequencing was performed at Shanghai Majorbio Biotech Co. Ltd. (China).

High performance liquid chromatography (HPLC) analysis was carried out on an Agilent 1260 HPLC system (Agilent Technologies Inc., USA) equipped with a DAD detector. Semi-preparative HPLC was performed on an Agilent 1100 system equipped with a DAD detector (Agilent Technologies Inc., USA). HPLC Electrospray ionization MS (HPLC-ESI-MS) and tandem MS (MS/MS) for small molecules were performed on a Thermo Fisher LTQ Fleet ESI-MS spectrometer (Thermo Fisher Scientific Inc., USA), and the data were analyzed using Thermo Xcalibur software. NanoLC-MS/MS and MS/MS for peptides were performed on an EASY-nLC 1200 (Thermo Fisher Scientific Inc., USA) coupled with a

Q Exactive HF mass spectrometer (Thermo Fisher Scientific Inc., USA), and the data were analyzed using pymzML, pFind[3] and Thermo Xcalibur. High resolution ESI-MS (HR-ESI-MS) analysis for small molecules was carried out on an Agilent 6230B Accurate Mass TOF LC/MS System (Agilent Technologies Inc., USA), and the data were analyzed using Agilent MassHunter Qualitative Analysis software. NMR data were recorded on an Agilent 500 MHz PremiumCompact+ NMR spectrometer (Agilent Technologies Inc., USA).

Protein expression and purification. The recombinant proteins CaeA1, PCP_{CaeA2}, CaeB1, ColB1, ColG2, CaeA3 and ColA3, as well as the chimeric NRPS proteins $CaeA3_{C}ColA3_{A\text{-}PCP}$, CaeA3-N' and ColA3-N' that arise from domain swapping, were produced in a form tagged by 6 × His at N-terminus or by both MBP and 6 × His at N-terminus, while CaeA2 and its variants (e.g., $CaeA2^{F2042L}$, $CaeA2^{F2042I}$, $CaeA2^{F2042V}$, Ct_{CaeA2}, CaeA2-△Ct, $CaeA2^{F2042L}$△Ct and $PCP\text{-}Ct_{CaeA2}$) were expressed in a form tagged by 8 × His at C-terminus, Trx and 6 × His at N-terminus or Sumo and 6 × His at N-terminus.

In general, the genes coding for the above proteins were amplified by PCR individually from the CAE-producing strain *Actinoalloteichus cyanogriseus* or the COL-producing strain *Streptomyces roseosporus*, both of which are listed in **Supplementary Table 1**, using the corresponding primers listed in **Supplementary Table 3**. The PCR products were first cloned into the vector pMD19-T for sequencing to confirm the fidelity and then into the expression vector pET-28a(+) (for N-terminal 6 × His-tagged proteins or N-terminal 6 × His and Sumo-tagged proteins), pSJ5 (for N-terminal 6 × His and Trx-tagged proteins), pQ8 (for N-terminal 6 × His and MBP-tagged proteins), or pET-37b(+) (for

C-terminal 8 × His proteins). For expression of the chimeric NRPS protein CaeA3$_{C}$ColA3$_{A\text{-}PCP}$ (containing the C domain of CaeA3 and the A and PCP domains of ColA3), a 1.4 kb DNA fragment amplified by PCR from *A. cyanogriseus* using the primers CaeA3-C-For and CaeA3-C-Rev was cloned into pMD19-T, yielding pQL1046. Similarly, a 1.8 kb DNA fragment amplified by PCR from *S. roseosporus* using the primers ColA3-APCP-For and ColA3-APCP-Rev was cloned into pMD19-T, yielding pQL1047. The 1.4 kb NdeI-NheI fragment and the 1.8 kb NheI-HindIII fragment were recovered and co-ligated into the NdeI-HindIII site of pET-28a(+) to give pQL1048. For expression of the chimeric NRPS protein CaeA3-N' (i.e., a CaeA3 variant with the docking region of ColA3), a 3.2 kb DNA fragment amplified by PCR from pQL1031 using the primers CaeA3-Core-For and CaeA3-Core-Rev was ligated with a 5.3 kb DNA fragment, which contain the ColA3 docking region and the pET28a vector and was amplified by PCR from pQL1037 using the primers ColA3-DR-For and ColA3-DR-Rev, giving pQL1057. For expression of the chimeric NRPS protein ColA3-N' (a ColA3 variant containing the docking region of CaeA3), a 3.2 kb DNA fragment amplified by PCR from pQL1037 using the primers ColA3-Core-For and ColA3-Core-Rev was cloned into the NdeI-HindIII site of pET-28a(+), giving pQL1058.

The resulting recombinant plasmids, which are listed in **Supplementary Table 2**, were transferred into *E. coli* BL21(DE3) for expression of CaeA2^{F2042L}, PCP$_{CaeA2}$, Trx-tagged Ct$_{CaeA2}$, CaeB1, MBP-fused CaeB1, ColG2 and ColB1 or into *E. coli* BAP1, an engineered strain capable of co-expressing the PPTase Sfp[4], for expression of CaeA1, CaeA3, ColA3, ColA3-N', CaeA3-N', CaeA3$_{C}$ColA3$_{A\text{-}PCP}$ and CaeA2 and its variants CaeA2^{F2042L}, CaeA2^{F2042I}, CaeA2^{F2042V}, CaeA2-△Ct, CaeA2^{F2042L}△Ct and Sumo-tagged PCP-Ct$_{CaeA2}$.

The culture of each *E. coli* transformant was incubated in Luria-Bertani (LB) medium containing 50 μg/mL kanamycin at 37°C and 220 rpm until the cell density reached 0.6-0.8 at OD_{600}. Protein expression was induced by the addition of isopropyl-β-D-thiogalactopyranoside (IPTG) to the final concentration of 0.1 mM, followed by further incubation for 20 h at 25˚C. The cells were harvested by centrifuging at 5000 rpm for 20 min at 4˚C and were re-suspended in 30 mL of lysis buffer (50 mM Tris-HCl, 300 mM NaCl, 10% glycerol, pH 7.8). After disruption by FB-110X Low Temperature Ultra-pressure Continuous Flow Cell Disrupter (Shanghai Litu Mechanical Equipment Engineering Co., Ltd, China), the soluble fraction was collected and subjected to purification of each target protein using a HisTrap FF column (GE Healthcare, USA). The desired protein fractions, as determined by SDS-PAGE, were concentrated and desalted using a PD-10 Desalting Column (GE Healthcare, USA) according to the manufacturer's protocols. The concentration of protein was determined by Bradford assay using bovine serum albumin (BSA) as the standard.

Sequence analysis. Open reading frames (ORFs) were identified using the FramePlot 4.0beta program (http://nocardia.nih.go.jp/fp4/). The deduced proteins were compared with other known proteins in the databases using available BLAST methods (http://blast.ncbi.nlm.nih.gov/Blast.cgi). Amino acid sequence alignments were performed using Vector NT1 and ESPript 3.0 (http://espript.ibcp.fr/ESPript/ESPript/).

Chemical synthesis of 4-hydroxy-2,2'-bipyridine-6-carboxyloyl-*S*-CoA (2,2'-bipyidinyl-*S*-CoA). The precursor 4-hydroxy-2,2'-bipyridinyl-6-carboxylic acid hydrobromide (**20**) was synthesized

according to the method described previously[4] .

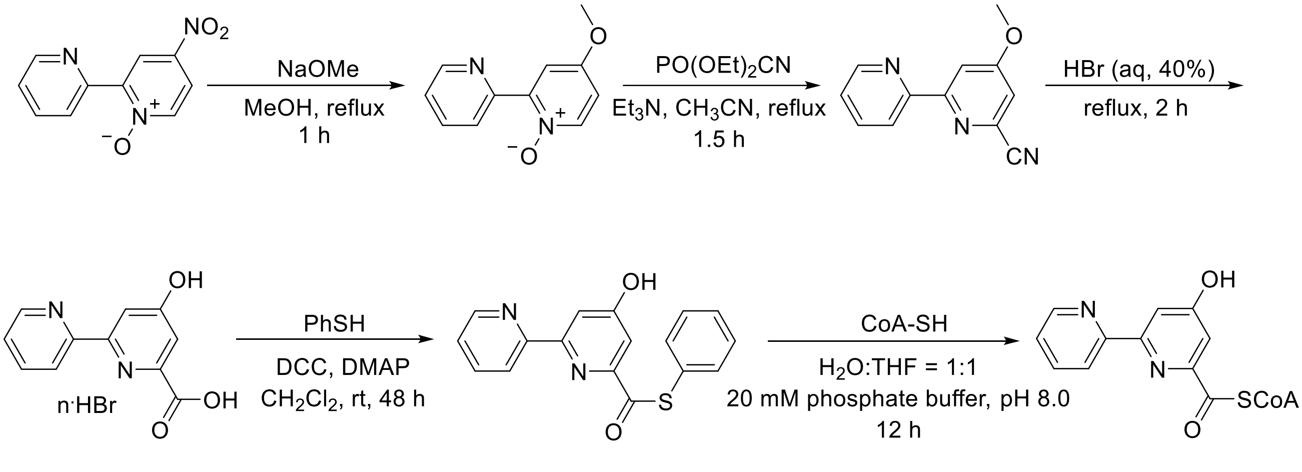

20 **18** **2,2'-bipyridinyl-*S*-CoA**

4-Hydroxy-2,2'-bipyridinyl-6-carboxylic acid hydrobromide (415 mg) was suspended in 15 mL of anhydrous CH_2Cl_2. Then, 721 mg of dicyclohexylcarbodiimide (DCC, 3.5 mmol), 0.58 mL of thiophenol (5.6 mmol) and 427 mg of 4-dimethylaminopyridine (DMAP, 3.5 mmol) were added into this CH_2Cl_2 suspension stirred at 0°C under argon atmosphere. The mixture was stirred at room temperature for 48 h before adding 0.3 mL of AcOH. After filtering, the organic phase of the reaction mixture was treated with saturated aqueous solution of $NaHCO_3$ and brine, and subsequent concentrated to dryness under reduced pressure. The resulting residue was re-dissolved in EtOAc, washed with 5% (w/v) citric acid solution, and filtered over diatomite. The organic phase was then dried over anhydrous Na_2SO_4 overnight, filtered, and concentrated under reduced pressure. Further purification was carried out by chromatography on silica gel using 2:1 *n*-hexane/EtOAc as the eluent, followed by concentration to yield *S*-phenyl 4-hydroxy-2,2'-bipyridinyl-6-carbothioate (**18**, 50.2 mg). for the purified compound, ^{1}H NMR (500 MHz, DMSO-d_6,) δ 11.48 (s, 1H), 8.72 (ddd, J = 4.3, 1.5,

0.8 Hz, 1H), 8.49 (d, *J* = 7.9 Hz, 1H), 8.07 (d, *J* = 2.3 Hz, 1H), 8.04 (td, *J* = 7.7, 1.8 Hz, 1H), 7.57 – 7.48 (m, 6H), 7.30 (d, *J* = 2.3 Hz, 1H); and ^{13}C NMR (125 MHz, DMSO-d_6) δ 191.7, 166.9, 157.1, 154.2, 152.4, 149.9, 138.1, 135.3, 129.9, 129.7, 128.6, 125.3, 121.2, 112.1, 108.5. ESI-HRMS Calcd. for $C_{17}H_{13}N_2O_2S^+$ 309.0698 $[M+H]^+$, found 309.0696.

Next, 30 mg of CoA-SH (0.039 mmol, Sangon Biotech, China) was dissolved in 1.8 mL phosphate buffer (40 mM, pH = 8.0), and the pH was adjusted to 8.0 with 1 M NaOH, followed by the addition of the solution of 18 mg of the above obtained *S*-phenyl 4-hydroxy-2,2'-bipyridinyl-6-carbothioate in 1.8 mL THF, stirring at room temperature under argon atmosphere for 12 h. After removal of THF under reduced pressure, the mixture was washed with ether (to remove the unreacted thioester), and then subjected to semi-preparation by HPLC to give 2,2'-bipyridinyl-*S*-CoA as a white powder (8 mg). This semi-preparation was carried out on an Aglient Zorbax column (SB-C18, 5 μm, 9.4 x 250 mm, Agilent Technologies Inc., USA) by gradient elution of solvent A (H_2O containing 5 mM NH_4Ac) and solvent B (CH_3CN) at a flow rate of 3 mL/min over a 30-min period as follows: T = 0 min, 5% B; T = 2 min, 5% B; T = 20 min, 90% B; T = 25 min, 90% B; T = 26 min, 5% B and T = 30 min, 5% B (mAU at 254 nm). For purified 2,2'-bipyridinyl-*S*-CoA, ESI-HRMS Calcd. for $C_{17}H_{13}N_2O_2S^+$ 966.1682 $[M+H]^+$, found 966.1670.

The CAE 2,2'-bipyridine intermediate **1** and the COL 2,2'-bipyridine intermediate **16** were synthesized using the methods previously reported[4].

Site-specific mutation of CaeA2 and its truncated proteins. Rolling cycle PCR amplification (using

the primers listed in **Supplementary Table 3**) followed by subsequent DpnI digestion was conducted according to the standard procedure of the Mut Express II Fast Mutagenesis Kit (Vazyme Biotech Co. Ltd, China). The yielded recombinant plasmids were listed in **Supplementary Table 2**. Each mutation was confirmed by Sanger sequencing. CaeA2^{F2042L}, CaeA2^{F2042L}ΔCt, CaeA2^{F2042I}, and CaeA2^{F2042V} were expressed in *E.coli* BAP1. All the mutant proteins were purified to homogeneity, and then concentrated according to the procedures for the native proteins described above.

Determination of the flavin cofactor. Each protein (CaeB1 or ColB1) solution at the concentration of 1 mg/ml was incubated at 100°C for 5 min for denaturation and then subjected to HPLC-DAD analysis on an Agilent Zorbax column (SB-C18, 5 μm, 4.6 x 250 mm, Agilent Technologies Inc., USA) by gradient elution of solvent A (H_2O containing 20 mM ammonium acetate) and solvent B (CH_3CN) at a flow rate of 1 mL/min over a 35-min period as follows: T = 0 min, 5% B; T = 2 min, 5% B; T = 20 min, 90% B; T = 25 min, 90% B; T = 30 min, 5% B and T = 35, 5% B (λ at 448 nm), using standard FAD as control. The supernatant of CaeB1 or ColB1 was subjected to ESI-HRMS analysis to confirmed the identity of FAD (ESI *m/z* $[M+H]^+$, calcd. 786.1644; found 786.1593).

Reconstitution of the 2,2'-bipyridine assembly line *in vitro*. The initial reaction was conducted at 30˚C for 1 h in a 100 μL reaction mixture containing 50 mM Tris-HCl (pH 7.5), 1 mM TCEP, 10 mM $MgCl_2$, 1 mM picolinic acid, 1 mM malonyl-*S*-CoA, 100 μM L-cysteine, 1 mM L-leucine, 10 μM CaeA1, 1 μM CaeA2, 1 μM CaeA3, and 4 mM ATP. To determine the necessary *trans* partner, CaeB1, CaeA4 or both of them were added into the above mixture, respectively, with the final concentration of 1 μM for each protein. Each reaction was quenched with 100 μL of CH_3CN after incubation. To

examine the production of the dethiolated 2,2'-bipyridine intermediate **1**, reaction mixtures were subjected to HPLC analysis on an Agilent Zorbax column (SB-C18, 5 μm, 4.6 x 250 mm, Agilent Technologies Inc., USA) using a DAD detector, by gradient elution of solvent A (H_2O containing 0.1% TFA) and solvent B (CH_3CN containing 0.1% TFA) at a flow rate of 1 mL/min over a 35-min period as follows: T = 0 min, 5% B; T = 2 min, 5% B; T = 20 min, 90% B; T = 25 min, 90% B; T = 30 min, 5% B and T = 35, 5% B (λ at 315 nm). For HPLC-ESI-MS analysis, TFA was replaced by 0.1% formic acid.

To evaluate the participation of the enzymes and substrates in the production of **1**, each component was omitted from the 100 μL reaction mixture that contains 50 mM Tris-HCl (pH 7.5), 1 mM TCEP, 10 mM $MgCl_2$, 1 mM picolinic acid, 1 mM malonyl-*S*-CoA, 100 μM L-cysteine, 1 mM L-leucine, 10 μM CaeA1, 1 μM CaeA2, 1 μM CaeA3, 1 μM CaeB1, and 4 mM ATP. To evaluate the mutation effects of CaeA2 on **1** production, CaeA2 was replaced with its variants CaeA2^{F2042L}, CaeA2^{F2042I}, CaeA2^{F2042V}, CaeA2-△Ct or CaeA2^{F2042L}△Ct. To mechanistically trace the transformation process, L-[1,2,3-$^{13}C_3$,^{15}N]cysteine and L-[2,3,3-D_3]cysteine were used to replace unlabeled L-cysteine, respectively. To evaluate the changeability of the enzymes in the reaction mixture, 1) CaeB1 was replaced with ColB1 (with the final concentration of 10 μM); and 2) CaeA3 was replaced with ColA3, CaeA3$_C$ColA3$_{A\text{-}PCP}$, CaeA3-N' and ColA3-N', respectively. Reactions are conducted at 30˚C for 1 h and then quenched with 100 μL of CH_3CN. The production of **1** was monitored by HPLC or HPLC-ESI-MS under conditions as described above.

For H_2S examination during **1** production, the reaction was conducted at 30˚C for 1 h in the 100 μL,

TCEP-involving reaction mixture that contains 50 mM Tris-HCl (pH 7.5), 1 mM TCEP, 10 mM $MgCl_2$, 1 mM picolinic acid, 1 mM malonyl-*S*-CoA, 100 μM L-cysteine, 1 mM L-leucine, 10 μM CaeA1, 1 μM CaeA2, 1 μM CaeA3, 1 μM CaeB1, and 4 mM ATP. Then, the TCEP derivative **2** was analyzed by HPLC-ESI-MS under conditions as described above. Alternatively, H_2S examination was conducted in the reaction mixture where TCEP was omitted, in the presence of **19** (1 μM), a dual-reactable fluorescent probe used for highly selective and sensitive detection of biological H_2S[5]. The reaction of **19** with Na_2S to yield **21** serves as the control reaction. **21** was examined by HPLC-FLD under conditions as described above with excitation at 370 nm and relative emission at 450 nm.

To examine whether the CAE assembly line provides the thiolated intermediate in COL biosynthesis, ColG2 (with the final concentration of 1 μM) and SAM (with the final concentration of 1 mM) were added into the 100 μL reaction mixture that contains 50 mM Tris-HCl (pH 7.5), 1 mM TCEP, 10 mM $MgCl_2$, 1 mM picolinic acid, 1 mM malonyl-*S*-CoA, 100 μM L-cysteine, 1 mM L-leucine, 10 μM CaeA1, 1 μM CaeA2, 1 μM CaeB1, 1 μM CaeA3 (ColA3, $CaeA3_C ColA3_{A\text{-}PCP}$, CaeA3-N' or ColA3-N'), and 4 mM ATP. Reactions are conducted at 30˚C for 1 h and then quenched with 100 μL of CH_3CN. The production of the thiolated 2,2'-bipyridine intermediate **16** was examined by HPLC or HPLC-ESI-MS under conditions as described above.

For sulfhydryl-2,2'-bipyridinyl-L-leucine (**15**) examination, the reaction was conducted at 30˚C for 1 h in the 50 μL reaction mixture contains 50 mM Tris-HCl (pH 7.5), 1 mM TCEP, 10 mM $MgCl_2$, 1 mM picolinic acid, 1 mM malonyl-*S*-CoA, 100 μM L-cysteine, 1 mM L-leucine, 10 μM CaeA1, 1 μM CaeA2, 1 μM ColA3, 1 μM CaeB1, and 4 mM ATP. The reaction mixture was treated with 1% SDS

and 5 mM TCEP at 55˚C for 30 min to release the free thiol of **15**, and then was incubated with 25 mM iodoacetamide (IAA) at 30˚C for 30 min. The production of **17** was examined by HPLC or HPLC-ESI-MS under conditions as described above.

To examine O_2 dependence under anaerobic conditions, gas exchange for O_2 elimination was conducted in an anaerobic glovebox overnight before the incubation of the related reaction mixture at 30˚C for 1 h. All assays were performed at least in triplicate and each had at least two parallel samples.

***In vitro* assays of PCP *S*-aminoacylation on CaeA2 by nanoLC-MS/MS.** The CaeA2 recombinant protein that was purified from *E.coli* BAP1 was subjected to complete or partial protease hydrolysis with trypsin, Glu-C or chymotrypsin as well as a variety of their combinations to map **SLGGDSIMGIQF$_{2042}$VSR** of CaeA2, the MS-detectable sequence that contains the Ppant-modified active-site L-serine residue (underlined). The digestion mixtures were filtrated using Microcon YM-10 (MilliporeSigma, USA) by centrifugation and stored at - 80°C before analysis. For nanoLC-MS/MS analysis, each sample was loaded on a trap column (75 µm i.d., 2 cm, C18, 5 µm, 100 Å, Thermo Fisher Scientific Inc., USA) for online desalting, and then was separated using a reversed phase column (75 µm i.d., 10.2 cm, C18, 3 µm, 120 Å, New Objective Inc., USA) by gradient elution of solvent A (H_2O containing 0.1% formic acid) and solvent B (80% CH_3CN containing 0.1% formic acid) at a flow rate of 300 nL/min over a 1.5 h period as follows: T = 0 min, 18% B; T = 45 min, 45% B; T = 50 min, 100% B; and T = 90 min, 100% B. For MS analysis, the nano-ESI voltage and capillary temperature were set at 2.2 kV and 275 ℃, respectively. The MS data were acquired in data-dependent mode. Each full-scan MS (*m/z* 350−2000, resolution of 60 k) was followed with 10 HCD MS/MS scans

(normalized collision energy of 33, resolution of 15 k) for the most intense precursor ions. The maximum ion injection time for MS and MS/MS were 50 and 45 ms, and the auto gain control target for MS and MS/MS were 3×10^6 and 5×10^4, respectively. The dynamic-exclusion time was set as 40 s.

To obtain the sequence **SLGGDSIMGIQF$_{2042}$VSR**, the 50 μL solution that contains 50 mM Tris-HCl (pH 7.5) and 5 μM CaeA2 was treated with 4 μg of trypsin (sequencing grade, Promega Corp., USA) at 30°C for 20 min (leading to complete digestion) and then with 0.6 μg of chymotrypsin (sequencing grade, Promega Corp., USA) at 30°C for 10 min (leading to partial digestion that retains the C-terminal sequence **F$_{2042}$VSR**). To obtain the engineered sequence **SLGGDSIMGIQL$_{2042}$VSR**, the CaeA2^{F2042L} recombinant protein that was purified from *E.coli* BAP1 and its variants different in *S*-(amino)acylation underwent complete digestion with trypsin (4 μg) and chymotrypsin (0.6 μg) at 30°C for 20 min.

To identify the target sequence that contains the Ppant-modified active-site L-serine residue, the raw MS data was processed using the pFind software by setting Ppant as a variable posttranslational modification. The selected sequence was then validated by HR-MS/MS analysis of the parent ion, its associated fragmented peptide ions and particularly the characteristic Ppant ejection ion[6].

To prepare L-cysteinyl-*S*-CaeA2^{F2042L} (**3**), the reaction was conducted at 30°C for 10 min in a 50 μL reaction mixture containing 50 mM Tris-HCl (pH 7.5), 1 mM TCEP, 10 mM $MgCl_2$, 100 μM L-cysteine, 5 μM CaeA2^{F2042L} (or CaeA2^{F2042L}△Ct) and 4 mM ATP. To prepare (3-sulfhydryl)-pyruvoyl-*S*-

CaeA2^{F2042L} (**6**), the reaction was conducted at 30˚C for 10 min in a 50 μL reaction mixture containing 50 mM Tris-HCl (pH 7.5), 1 mM TCEP, 10 mM $MgCl_2$, 100 μM L-cysteine, 5 μM CaeA2^{F2042L} (or CaeA2^{F2042L}△Ct), 1 μM CaeB1 and 4 mM ATP. To prepare 2,2'-bipyridinyl-*S*-CaeA2^{F2042L} (**9**), the reaction was conducted at 30˚C for 10 min in a 50 μL reaction mixture containing 50 mM Tris-HCl (pH 7.5), 1 mM TCEP, 10 mM $MgCl_2$, 1 mM picolinic acid, 1 mM malonyl-*S*-CoA, 100 μM L-cysteine, 1 μM CaeA1, 1 μM CaeB1, 5 μM CaeA2^{F2042L} (or CaeA2^{F2042L}△Ct) and 4 mM ATP. For isotope labeling, L-[1,2,3-$^{13}C_3$,^{15}N]cysteine and L-[2,3,3-D_3]cysteine were used to replace unlabeled L-cysteine, respectively. For thiol derivatization, each reaction mixture was treated with 10 mM iodoacetamide (IAA) at 30°C for 2 min. Protease digestion and subsequent nanoLC-MS/MS analyses were conducted using approaches as described above.

To probe potential intermediates, all MS/MS data of the derivatives from the sequence (**SLGGDSIMGIQL$_{2042}$VSR)** were extracted from raw MS data using script written by Python (https://github.com/billpb610/AlFinder/blob/master/AlFinderS2.py). The script also recorded information about precursor ions, fragment iond and Ppant ejection ions for further analysis. All examinations were performed at least in triplicate and each had at least two parallel samples.

***In vitro* preparation of 2,2'-bipyridinyl-*S*-CaeA2^{F2042L} (9).** To prepare **9** as a positive control in *S*-aminoacylation assays, the reaction was conducted at 30˚C for 30 min in a 50 uL reaction mixture containing 50 mM Tris-HCl (pH 7.5), 1 mM TCEP, 10 mM $MgCl_2$, 100 μM 2,2'-bipyridinyl-*S*-CoA, 5 μM Sfp, and 10 μM CaeA2^{F2042L} in apo-form. Protease digestion and subsequent nanoLC-MS/MS analyses were conducted using approaches as described above.

***In vitro* preparation of 2,2'-bipyridinyl-*S*-PCP$_{\mathbf{CaeA2}}$ (10).** The reaction was conducted at 30˚C for 1 h in an 80 μL reaction mixture containing 62.5 mM Tris^{-}HCl (pH = 7.5), 1.25 mM TCEP, 12.5 mM $MgCl_2$, 1 mM 2,2'-bipyridinyl-*S*-CoA, 100 μM PCP$_{CaeA2}$ (derived from wild-type CaeA2) in apo-form, and 4 μM Sfp.

To produce the dethiolated 2,2'-bipyridine intermediate **1**, the reaction mixture was combined with the following components in a new 100 μL reaction mixture: 1 mM L-leucine, 5 μM CaeA3, and 4 mM ATP. After incubation at 30˚C for 2 h, the reaction was quenched with 100 μL of CH_3CN for HPLC or HPLC-ESI-MS analysis under conditions as described above.

Measurement of protein-protein interactions by isothermal titration calorimetry (ITC). ITC was performed with MicroCal-ITC200 (Malvern) at 25℃. A 400 μl aliquot of 60 μM PCP-Ct$_{CaeA2}$, Ct, PCP$_{CaeA2}$ or CaeA2△Ct was placed in the stirred cell, and 120 μl aliquot of 400 μM MBP-fused CaeB1 or ColB1 was prepared in the syringe. All the recombinant proteins were prepared by Ni-affinity chromatography followed by size-exclusion chromatography (SEC) for purification, and then were exchanged in the 50 mM Tris-HCl (PH = 7.5) buffer containing 100 mM NaCl, 0.1 mM TCEP and 5 % glycerol. The titration was performed as follows: 1 μl of protein in the syringe over 0.8 s for the first injection, followed by 19 injections of 2 μl protein in the stirred cell at 120 s intervals. The heat of reaction per injection was determined by integration of the peak areas using the MicroCal-PEAQ-ITC software, which provides the best-fit values for the heat of binding (ΔH), the stoichiometry of binding (N), and the dissociation constant (K_d). The heats of dilution were determined by injecting flavoprotein

alone into the buffer and were subtracted from the corresponding experiments before curve fitting. All assays were performed at least in triplicate and each had at least two parallel samples.

Analysis of the functional exchangeability between CaeA2 and ColA2 *in vivo.* A 4.4 kb DNA fragment amplified by PCR from the CAE-producing *A. cyanogriseus* strain using the primers cae-CO-for and cae-Ct-rev was cloned into pMD19-T, yielding pQL1053. After sequencing to confirm the fidelity, this PCR product was recovered by digestion with BglII-EcoRV and then utilized to replace the 4.4 kb BglII-EcoRV fragment of pQL1022[7], a pSET152 derivative previously constructed for *colA2* expression under the control of *PermE** (the constitutive promoter for expressing the erythromycin-resistance gene in *Saccharopolyspora erythraea*). The resulting recombinant plasmid pQL1054, which carries the chimeric gene *col/caeA2* coding for the hybrid protein that harbors the PKS module from ColA2 and the NRPS module from CaeA2, was transferred by conjugation into the *△colA2 S. roseosporus* mutant strain[7], yielding the recombinant strain QL2006. The fermentation of QL2006 and the examination of CAE or COL production were conducted according the methods described previously[7].

SUPPLIMENTARY FIGURES

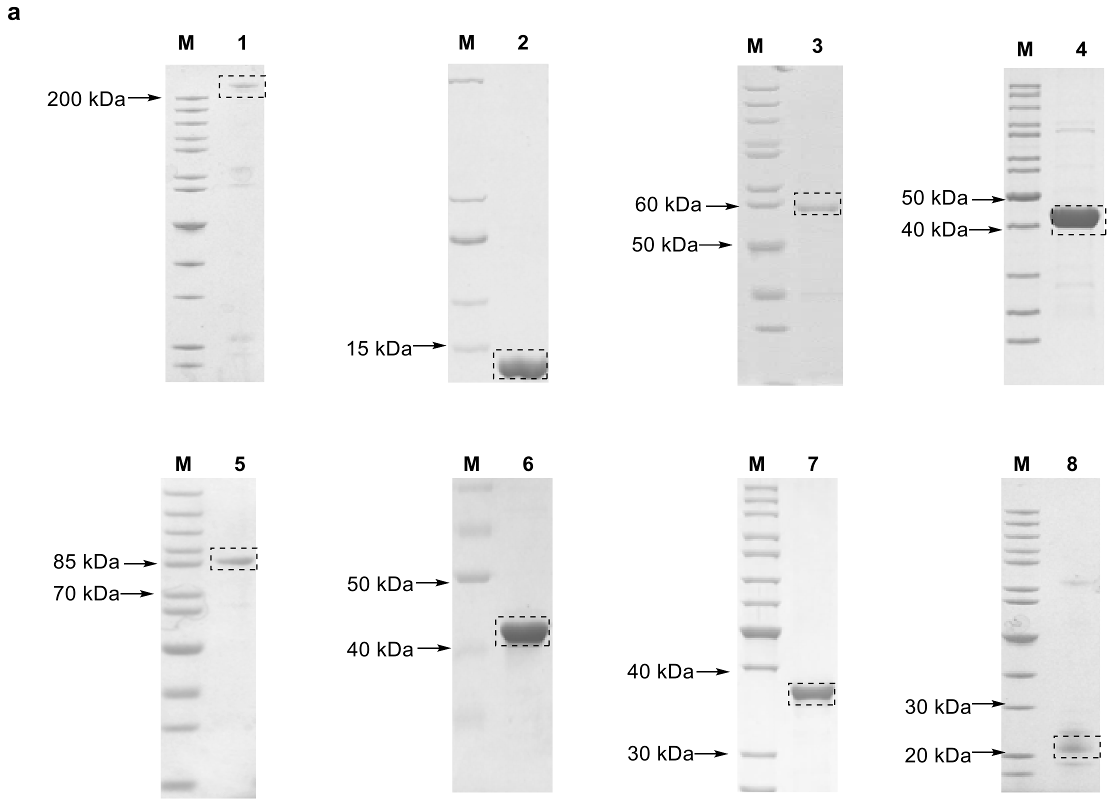

Supplementary Figure 1. Coomassie-stained SDS-PAGE analysis of purified recombinant proteins. Lane M, protein standard. The target proteins are indicated in the dashed rectangles. All results were repeated at least 3 times independently with similar results.

(**a**) Proteins produced in *E. coli* BL21(DE3). Lane 1, CaeA2^{F2042L} (269 kDa); Lane 2, PCP$_{CaeA2}$ (derived from CaeA2, 13 kDa); Lane 3, Trx-fused Ct$_{CaeA2}$ (58 kDa), Lane 4, CaeB1 (43 kDa); Lane 5, MBP-fused CaeB1 (87 kDa), Lane 6, ColB1 (43 kDa); Lane 7, ColG2 (39 kDa); and Lane 8, CaeA4 (25 kDa).

b

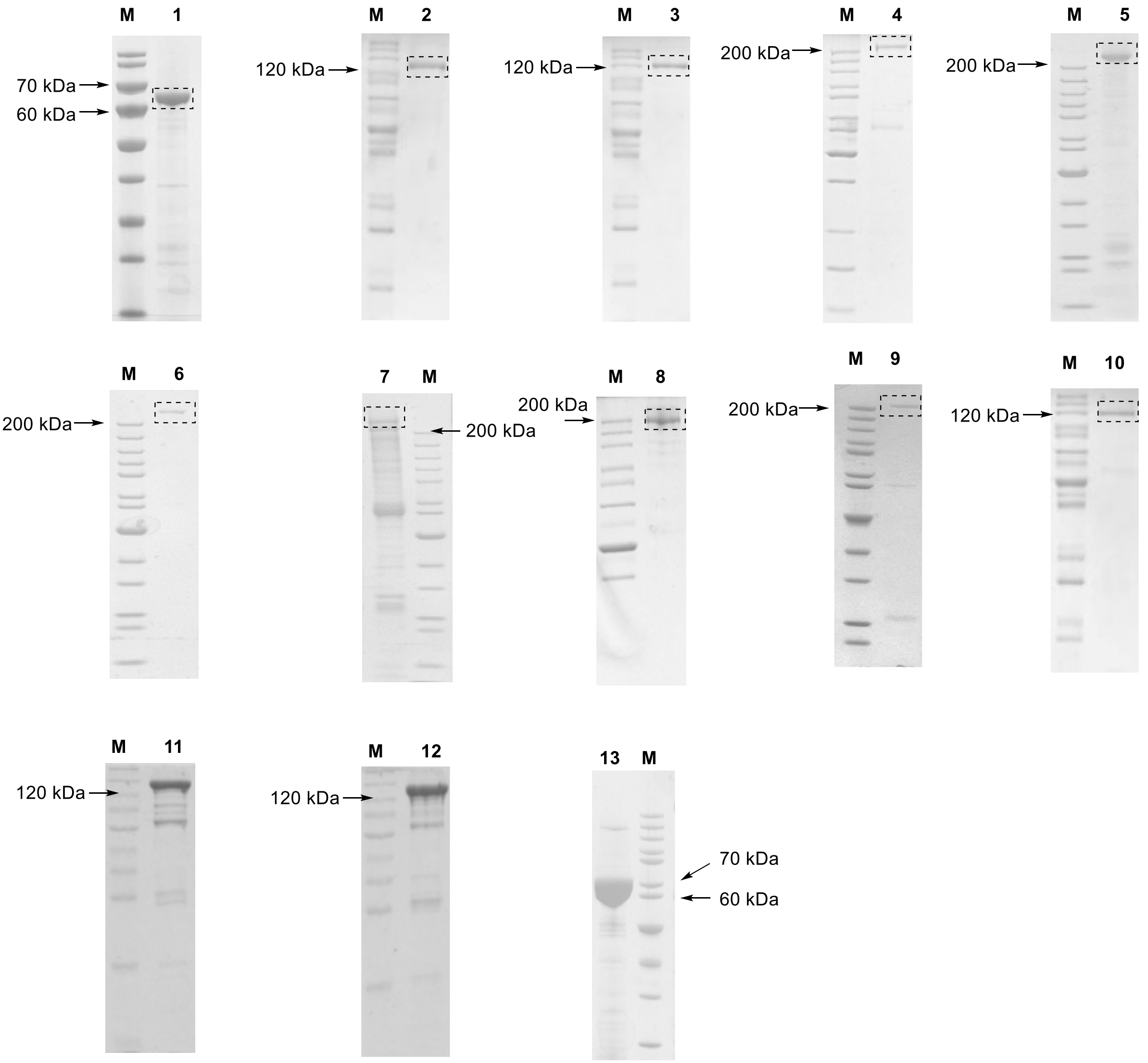

(**b**) Proteins produced in *E. coli* BAP1. Lane 1, CaeA1 (69 kDa); Lane 2, CaeA3 (118 kDa); Lane 3, ColA3 (121 kDa); Lane 4, CaeA2 (269 kDa); Lane 5, CaeA2^{F2042L} (269 kDa); Lane 6, CaeA2^{F2042I} (269 kDa); Lane 7, CaeA2^{F2042V} (269 kDa); Lane 8, CaeA2-△Ct (225 kDa); Lane 9, CaeA2^{F2042L}△Ct (225 kDa); Lane 10, CaeA3$_{C}$ColA3$_{A\text{-}PCP}$ (120 kDa); Lane 11, CaeA3-N’ (121 kDa); Lan 12, ColA3-N’ (120 kDa); and Lane 13, SUMO-fused PCP-Ct (62 kDa).

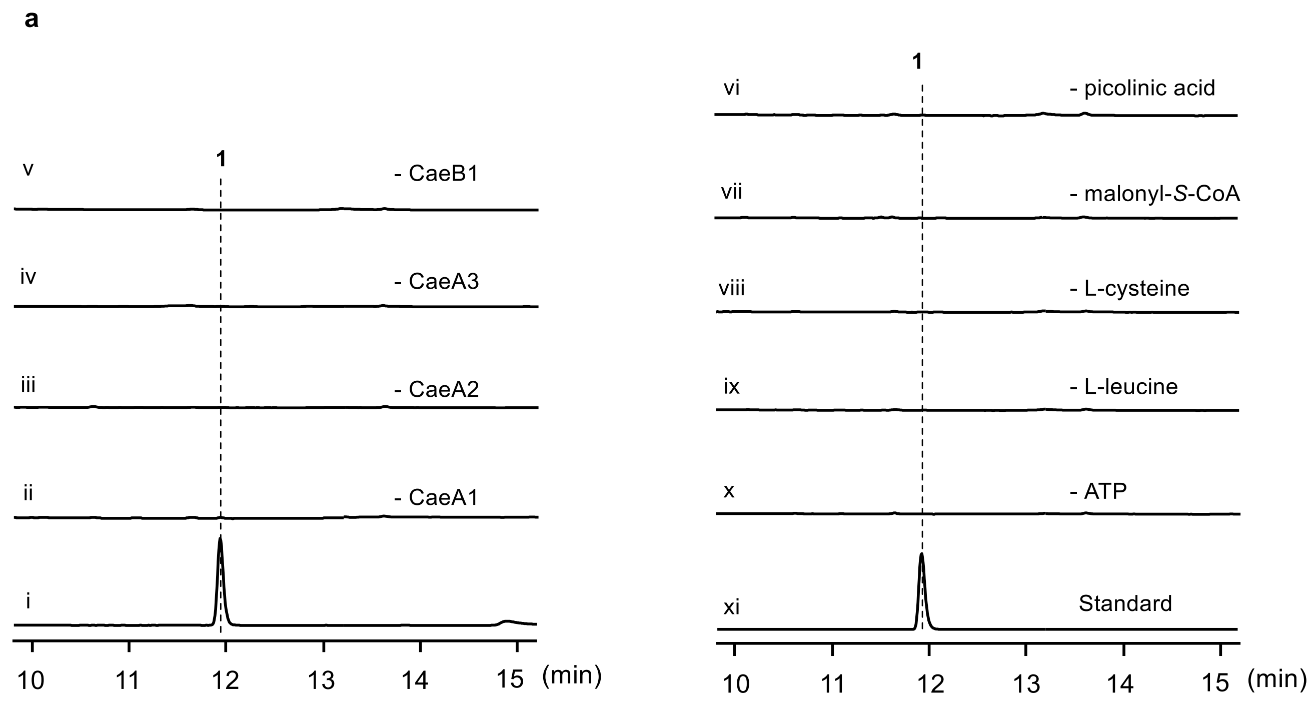

Supplementary Figure 2. Supplementary data for *in vitro* reconstitution of the 2,2'-bipyridine assembly line.

(**a**) Determination of component necessity in the production of the CAE 2,2'-bipyridine intermediate **1**. Tested reactions were derived from the combination of CaeA1, CaeA2, CaeA3 and CaeB1 with picolinic acid, malonyl-*S*-CoA, L-cysteine, L-leucine, and ATP (i), and included those lacking the enzyme CaeA1 (ii), CaeA2 (iii), CaeA3 (iv) or CaeB1 (v) and the substrate picolinic acid (vi), malonyl-*S*-CoA (vii), L-cysteine (viii), L-leucine (ix) or ATP (x, for the activity of the A domains), respectively. Synthesized **1** was used as the standard (xi).

b

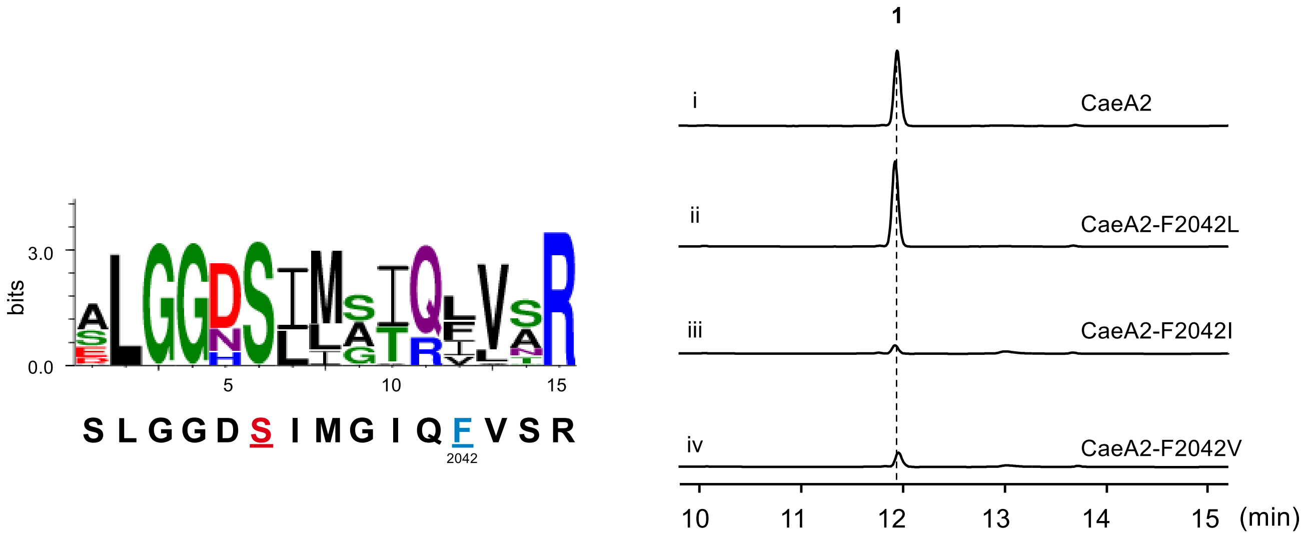

(**b**) Activity assays of CaeA2 variants in the production of **1**. The CaeA2 variants, i.e., CaeA2^{F2042L}, CaeA2^{F2042V} and CaeA2^{F2042I}, were designed according to residue conservative analysis (left), which was conducted by comparing the 15-aa sequence **SLGGDSIMGIQF$_{2042}$VSR** that is derived from the PCP domain of CaeA2 (the Ppant-modified active-site L-serine residue and the target L-phenylalanine residue are highlighted in color and underlined) with the corresponding sequences of 500 homologous PCP domains or proteins from NCBI NR database. Tested reactions were derived from the combination of CaeA1, CaeA2, CaeA3 and CaeB1 with picolinic acid, malonyl-*S*-CoA, L-cysteine, L-leucine, and ATP (i), and included those in which CaeA2 was replaced with CaeA2^{F2042L} (ii), CaeA2^{F2042V} (iii) and CaeA2^{F2042I} (iv), respectively.

a

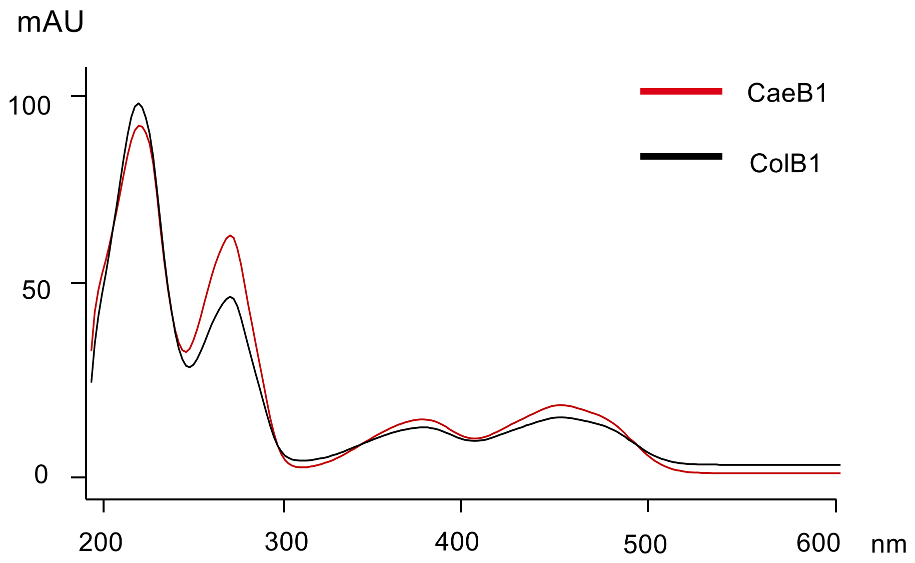

Supplementary Figure 3. Analysis of the flavoproteins CaeB1 and ColB1.

(**a**) UV spectra of the recombinant proteins CaeB1 and ColB1 that were purified from *E. coli* BL21(DE3).

(**b)** Determination of flavin cofactors associated with CaeB1 and ColB1. (i) authentic FMN; (ii) authentic FAD, (iii) boiled CaeB1, (iv) boiled CaeB1-S168A, (v) boiled CaeB1-E372A, and (vi) boiled ColB1. For examination by HPLC, UV absorbance at 375 nm.

(**c**) Activity assays of CaeB1 variants in the production of **1**. Tested reactions were derived from the combination of CaeA1, CaeA2, CaeA3 and CaeB1 with picolinic acid, malonyl-*S*-CoA, L-cysteine, L-leucine, and ATP (i), and included those in which CaeB1 was replaced with CaeB1-E372A (ii), CaeB1-S168A (iii) , and CaeB1-S168A with excess of FAD (iv), respectively.

b

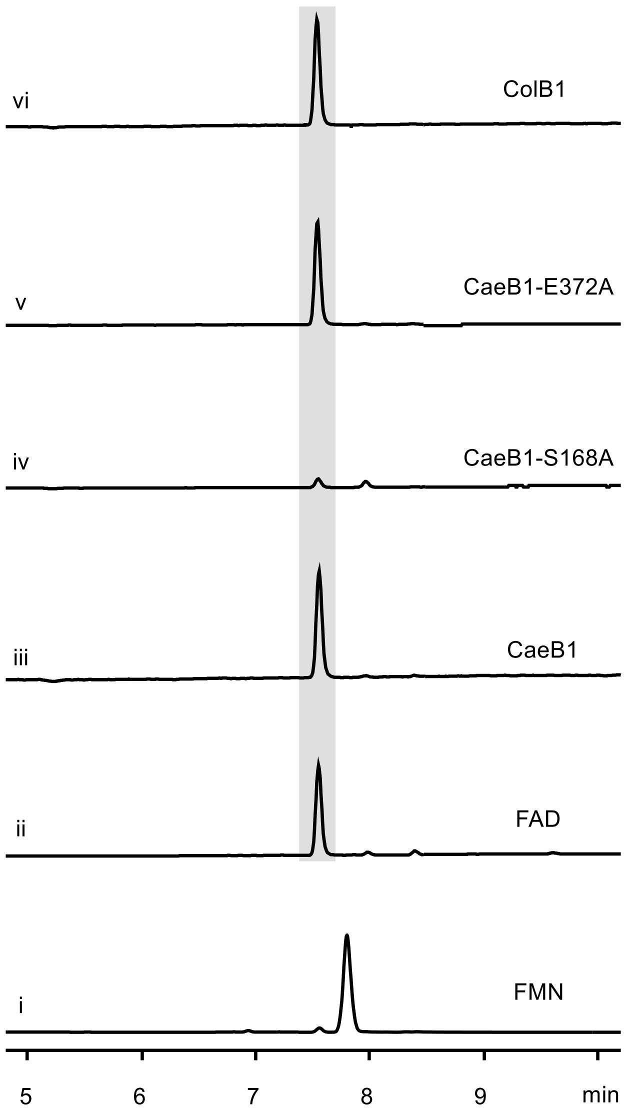

vi
ColB1
v
CaeB1-E372A
iv
CaeB1-S168A
iii
CaeB1
ii
FAD
i
FMN
5
6
7
8
9
min

c

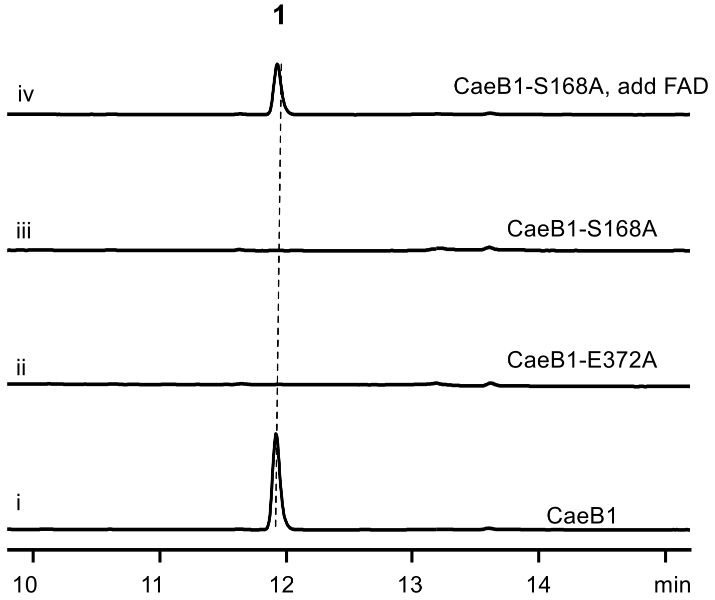

1
iv
CaeB1-S168A, add FAD
iii
CaeB1-S168A
ii
CaeB1-E372A
i
CaeB1
10
11
12
13
14
min

d

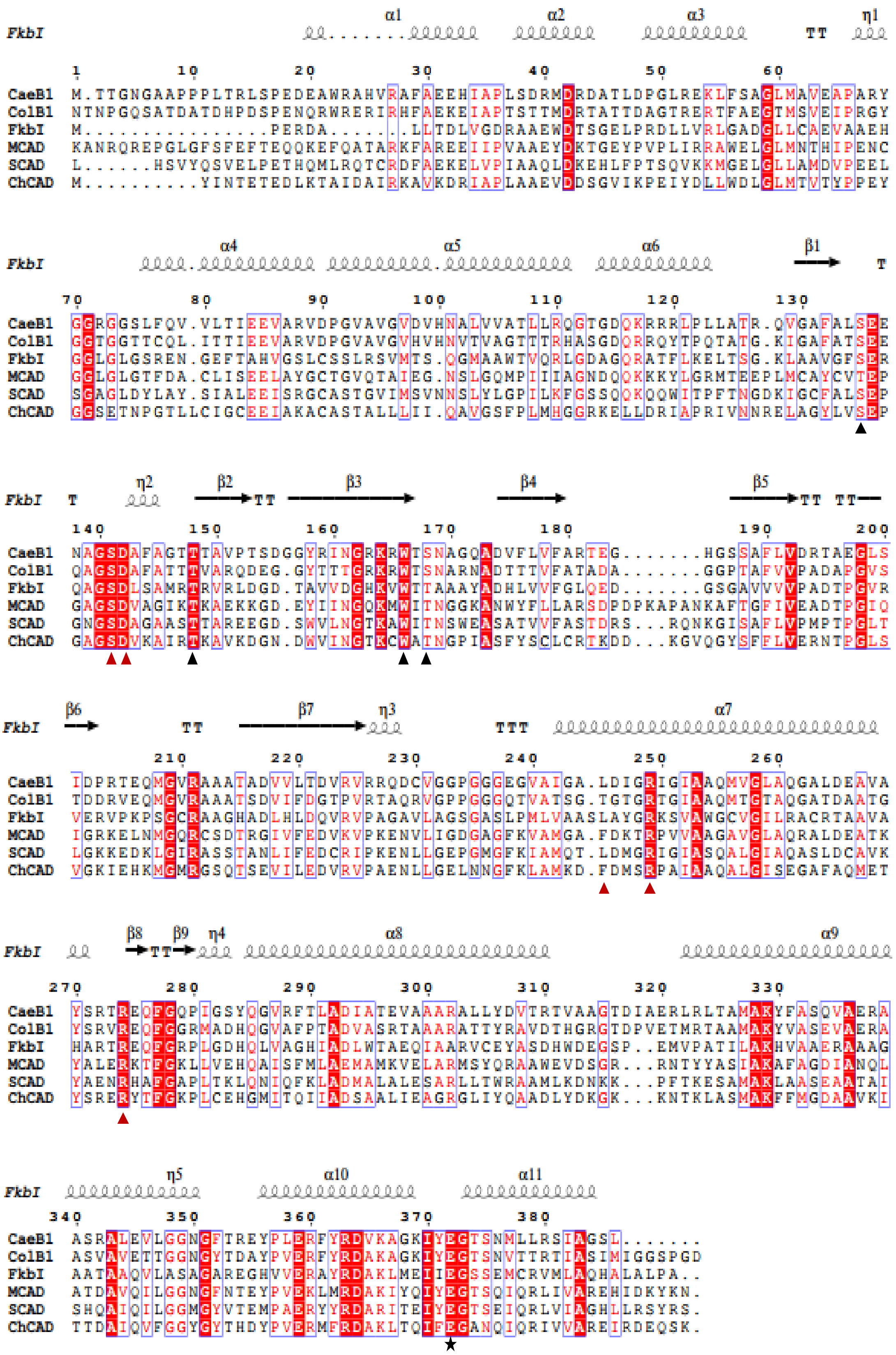
FkbI
α1 α2 α3 η1 α4 α5 α6 β1 η2 β2 β3 β4 β5 β6 β7 η3 α7 β8 β9 η4 α8 α9 η5 α10 α11
CaeB1 M.TTGNGAAPPPLTRLSPEDEAWRAHVRAFAEEHIAPLSDRMDRDATLDPGLREKLFSAGLMAVEAPARY
ColB1 NTNPGQSATDATDHPDSPENQRWRERIRHFAEKEIAPTSTTMDRTATTDAGTRERTFAEGTMSVEIPRGY
FkbI M................PERDA......LLTDLVGDRAAEWDTSGELPRDLLVRLGADGLLCAEVAAEH
MCAD KANRQREPGLGFSFEFTEQQKEFQATARKFAREEIIPVAAEYDKTGEYPVPLIRRAWELGLMNTHIPENC
SCAD L......HSVYQSVELPETHQMLRQTCRDFAEKELVPIAAQLDKEHLFPTSQVKKMGELGLLAMDVPEEL
ChCAD M..........YINTETEDLKTAIDAIRKAVKDRIAPLAAEVDDSGVIKPEIYDLLWDLGLMTVTYPPEY
CaeB1 GGRGGSLFQV.VLTIEEVARVDPGVAVGVDVHNALVVATLLRQGTGDQKRRRLPLLATR.QVGAFALSEE
ColB1 GGTGGTTCQL.ITTIEEVARVDPGVAVGVHVHNVTVAGTTTRHASGDQRRQYTPQTATG.KIGAFATSEE
FkbI GGLGLGSREN.GEFTAHVGSLCSSLRSVMTS.QGMAAWTVQRLGDAGQRATFLKELTSG.KLAAVGFSER
MCAD GGLGLGTFDA.CLISEELAYGCTGVQTAIEG.NSLGQMPIIIAGNDQQKKKYLGRMTEEPLMCAYCVTEP
SCAD SGAGLDYLAY.SIALEEISRGCASTGVIMSVNNSLYLGPILKFGSSQQKQQWITPFTNGDKIGCFALSEP
ChCAD GGSETNPGTLLCIGCEEIAKACASTALLLII.QAVGSFPLMHGGRKELLDRIAPRIVNNRELAGYLVSEP
CaeB1 NAGSDAFAGTTTAVPTSDGGYRINGRKRWTSNAGQADVFLVFARTEG.......HGSSAFLVDRTAEGLS
ColB1 QAGSDAFATTTVARQDEG.GYTTTGRKRWTSNARNADTTTVFATADA.......GGPTAFVVPADAPGVS
FkbI QAGSDLSAMRTRVRLDGD.TAVVDGHKVWTTAAAYADHLVVFGLQED.......GSGAVVVVPADTPGVR
MCAD GAGSDVAGIKTKAEKKGD.EYIINGQKMWITNGGKANWYFLLARSDPDPKAPANKAFTGFIVEADTPGIQ
SCAD GNGSDAGAASTTAREEGD.SWVLNGTKAWITNSWEASATVVFASTDRS...RQNKGISAFLVPMPTPGLT
ChCAD GAGSDVKAIRTKAVKDGN.DWVINGTKCWATNGPIASFYSCLCRTKDD...KGVQGYSFFLVERNTPGLS
CaeB1 IDPRTEQMGVRAAATADVVLTDVRVRRQDCVGGPGGGEGVAIGA.LDIGRIGIAAQMVGLAQGALDEAVA
ColB1 TDDRVEQMGVRAAATSDVIFDGTPVRTAQRVGPPGGGQTVATSG.TGTGRTGIAAQMTGTAQGATDAATG
FkbI VERVPKPSGCRAAGHADLHLDQVRVPAGAVLAGSGASLPMLVAASLAYGRKSVAWGCVGILRACRTAAVA
MCAD IGRKELNMGQRCSDTRGIVFEDVKVPKENVLIGDGAGFKVAMGA.FDKTRPVVAAGAVGLAQRALDEATK
SCAD LGKKEDKLGIRASSTANLIFEDCRIPKENLLGEPGMGFKIAMQT.LDMGRIGIASQALGIAQASLDCAVK
ChCAD VGKIEHKMGMRGSQTSEVILEDVRVPAENLLGELNNGFKLAMKD.FDMSRPAIAAQALGISEGAFAQMET
CaeB1 YSRTREQFGQPIGSYQGVRFTLADIATEVAAARALLYDVTRTVAAGTDIAERLRLTAMAKYFASQVAERA
ColB1 YSRVREQFGGRMADHQGVAFPTADVASRTAAARATTYRAVDTHGRGTDPVETMRTAAMAKYVASEVAERA
FkbI HARTREQFGRPLGDHQLVAGHIADLWTAEQIAARVCEYASDHWDEGSP..EMVPATILAKHVAAERAAAG
MCAD YALERKTFGKLLVEHQAISFMLAEMAMKVELARMSYQRAAWEVDSGR...RNTYYASIAKAFAGDIANQL
SCAD YAENRHAFGAPLTKLQNIQFKLADMALALESARLLTWRAAMLKDNKK...PFTKESAMAKLAASEAATAI
ChCAD YSRERYTFGKPLCEHGMITQIIADSALIEAGRGLIYQAADLYDKGK...KNTKLASMAKFFMGDAAVKI
CaeB1 ASRALEVLGGNGFTREYPLERFYRDVKAGKIYEGTSNMLLRSIAGSL.......
ColB1 ASVAVETTGGNGYTDAYPVERFYRDAKAGKIYEGTSNVTTRTIASIMIGGSPGD
FkbI AATAAQVLASAGAREGHVVERAYRDAKLMEIIEGSSEMCRVMLAQHALALPA..
MCAD ATDAVQILGGNGFNTEYPVEKLMRDAKIYQIYEGTSQIQRLIVAREHIDKYKN.
SCAD SHQAIQILGGMGYVTEMPAERYYRDARITEIYEGTSEIQRLVIAGHLLRSYRS.
ChCAD TTDAIQVFGGYGYTHDYPVERMFRDAKLTQIFEGANQIQRIVVAREIRDEQSK.

(**d**) Sequence alignment of CaeB1 and ColB1 with various FAD-dependent dehydrogenases. The homologs include FkbI (1R2G) in FK520 biosynthesis[8], medium chain acyl-CoA dehydrogenase (MCAD, 1T9G_A)[9], short chain acyl-CoA dehydrogenase (SCAD, 1JQI_A)[10] and cyclohexanecarboxyl-CoA dehydrogenase (ChCAD, ABC76100.1)[11]. Residues important for dehydrogenase activity, FAD-binding and phosphopantetheine binding are indicated by star, black triangle and red triangle, respectively.

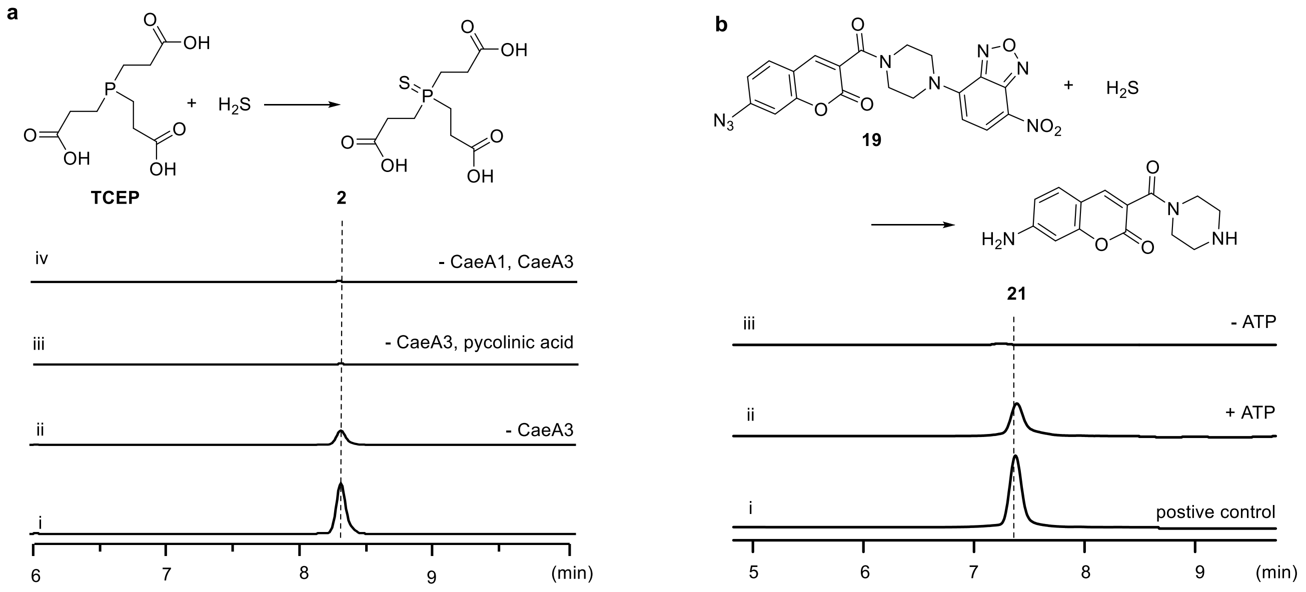

Supplementary Figure 4. H_2S production during the *in vitro* reconstitution of the 2,2'-bipyridine assembly line. (**a**) H_2S examination in TCEP-containing reaction mixtures by HPLC-MS. Electrospray ionization (ESI) *m/z* $[M + H]^+$ at 283.0405 (calcd.) for **2**, which is generated by a reaction of H_2S with TCEP. The initial reaction was conducted by combining CaeA1, CaeA2, CaeA3 and CaeB1 with picolinic acid, malonyl-*S*-CoA, L-cysteine, L-leucine, and ATP (i), and reactions in the absence of CaeA3 (ii), picolinic acid and CaeA3 (iii) or CaeA1 and CaeA3 (iv) were also conducted. (**b**) H_2S examination in the TCEP-free reaction mixtures by HPLC using a fluorescence detector (excitation at 370 nm and emission at 450 nm). The reaction of the fluorescent probe **19** with Na_2S to yield **21** was used as the positive control (i). H_2S production was examined in the reactions where CaeA1, CaeA2, CaeA3 and CaeB1 were combined with picolinic acid, malonyl-*S*-CoA, L-cysteine and L-leucine in the presence (ii) and absence (iii) of ATP.

a

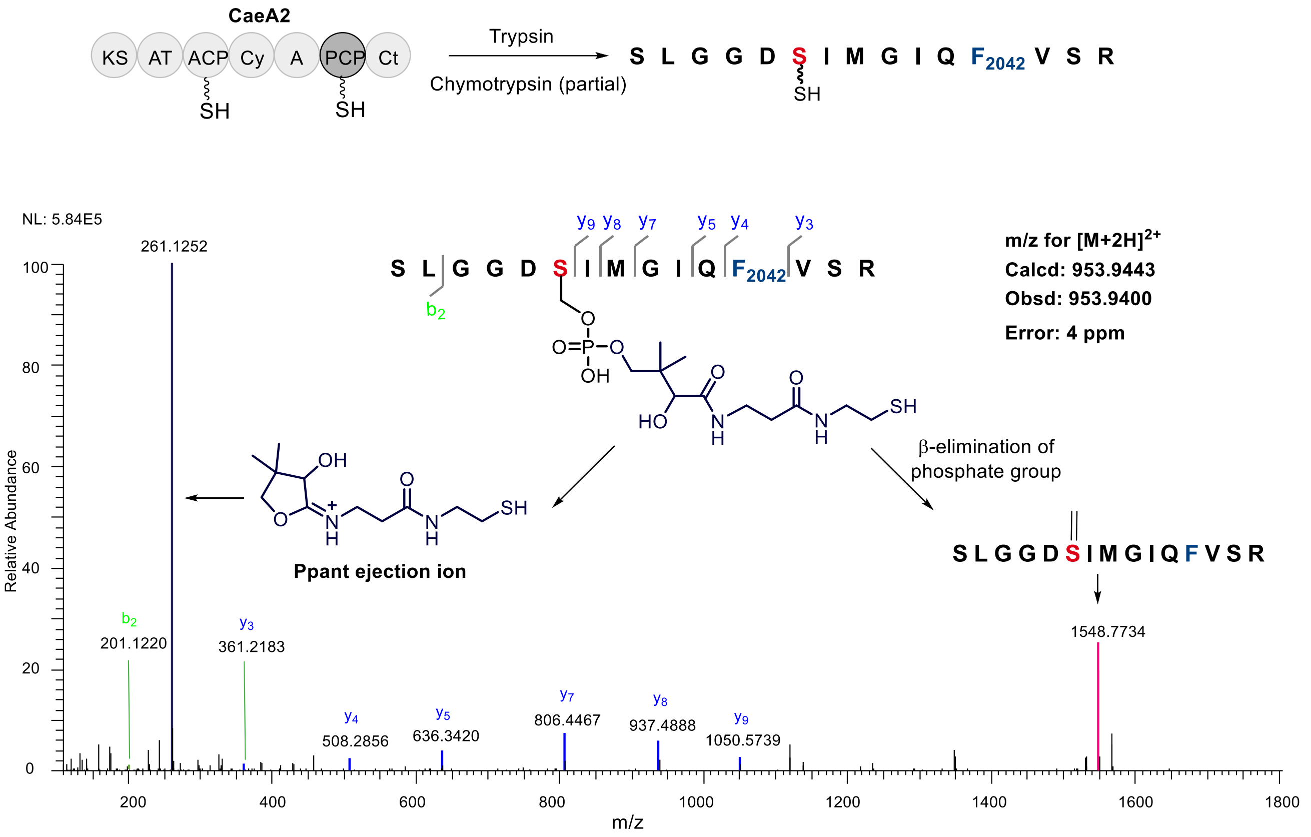

Supplementary Figure 5. Determination of the 15-aa sequence that is derived from the PCP domain of CaeA2 and contains the Ppant-modified active-site L-serine residue (red) by nanoLC-MS/MS. (**a**) Ppant-modified CaeA2 by treatment with trypsin (complete digestion) and chymotrypsin (partial digestion).

b

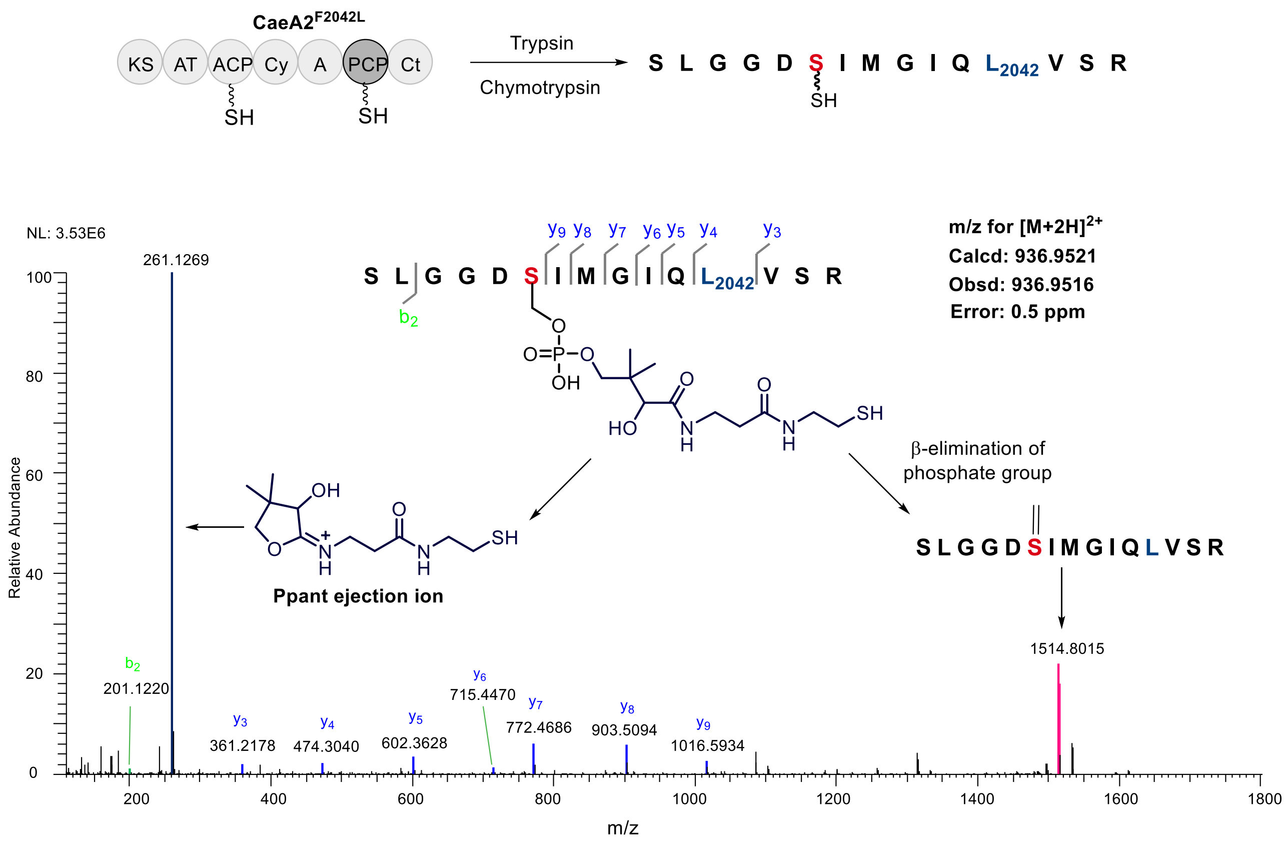

(**b**) Ppant-modified CaeA2^{F2042L} by treatment with trypsin (complete digestion) and chymotrypsin (complete digestion).

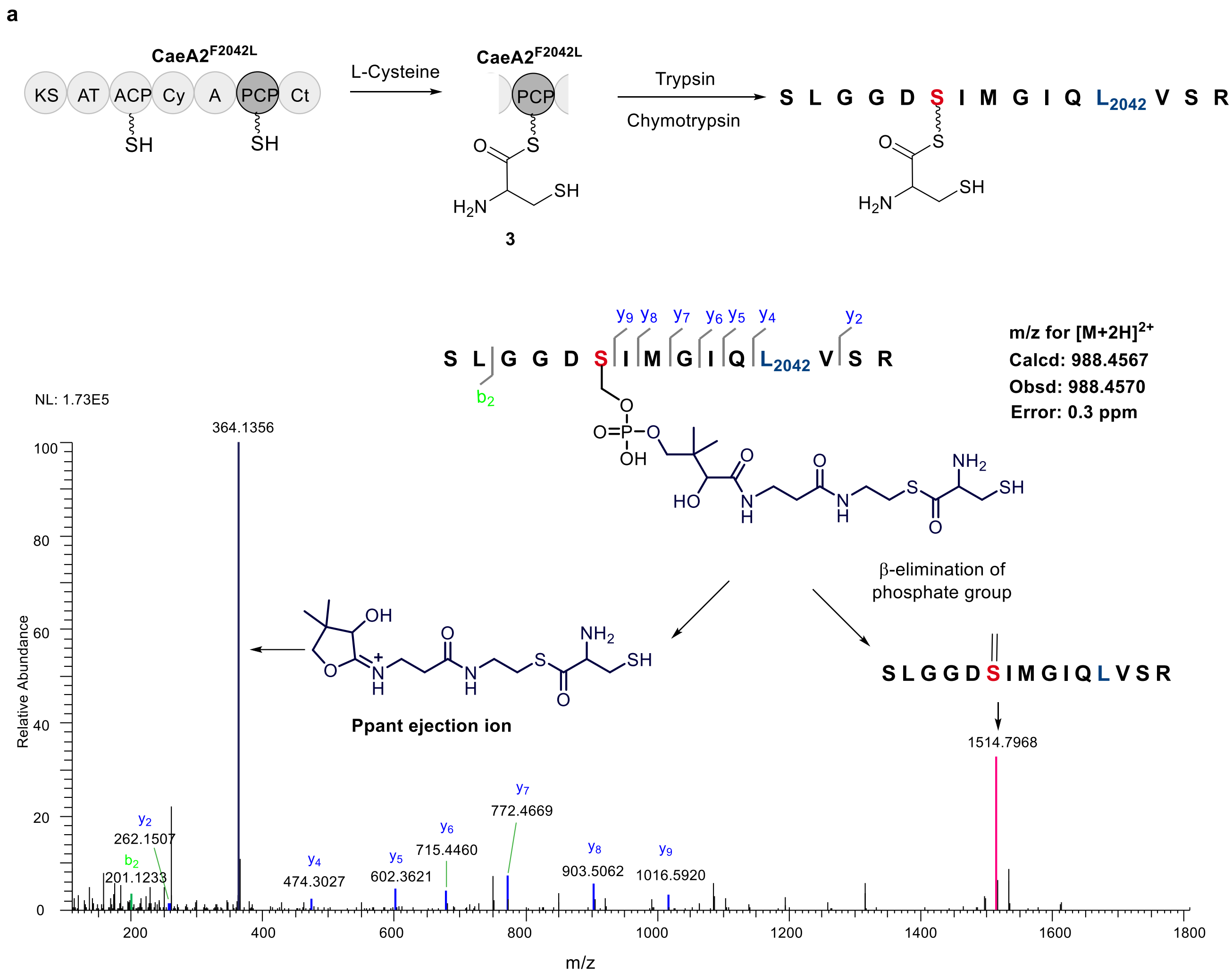

Supplementary Figure 6. Characterization of L-cysteinyl-*S*-CaeA2^{F2042L} (**3**) by nanoLC-MS/MS following complete digestion with trypsin and chymotrypsin. (**a**) Incubation of thiolated CaeA2^{F2042L} and L-cysteine.

b

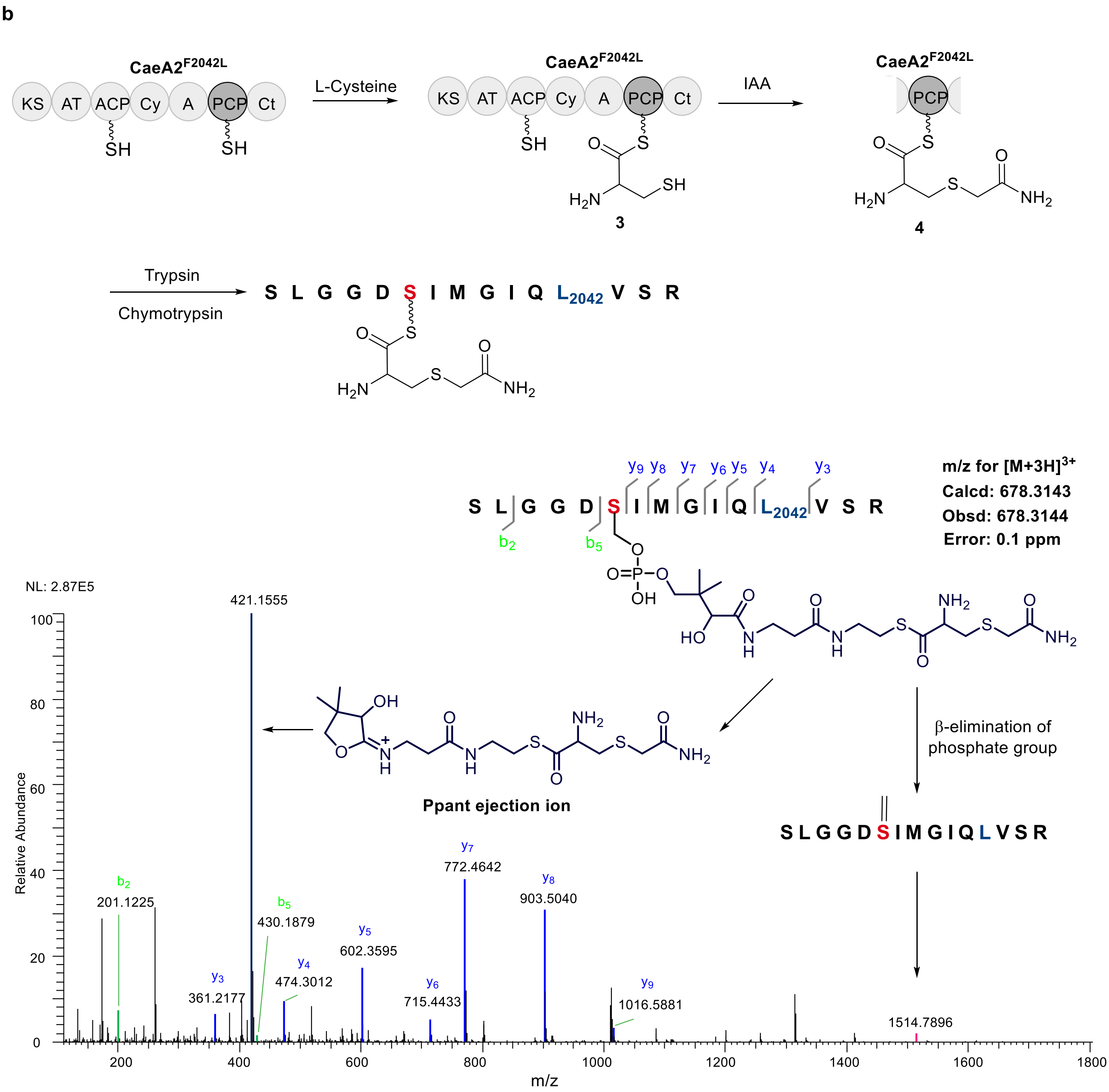

(**b**) Treatment of with IAA after the incubation of thiolated CaeA2^{F2042L} and L-cysteine.

a

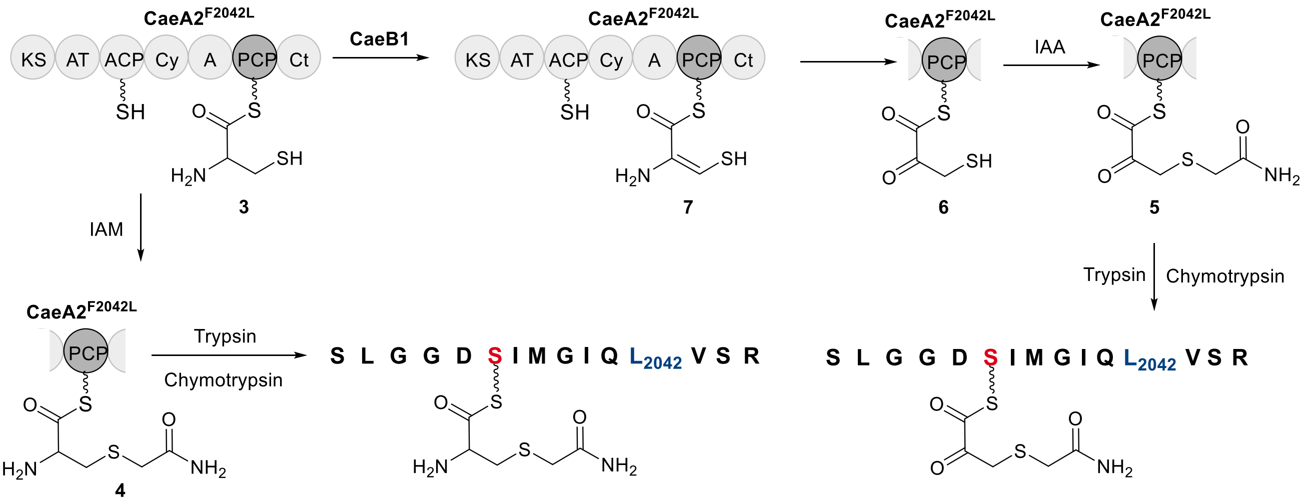

CaeA2^F2042L
KS AT ACP Cy A PCP Ct
CaeB1
SH
3
7
6
5
IAA
IAM
Trypsin
Chymotrypsin
4
S L G G D S I M G I Q L2042 V S R

i

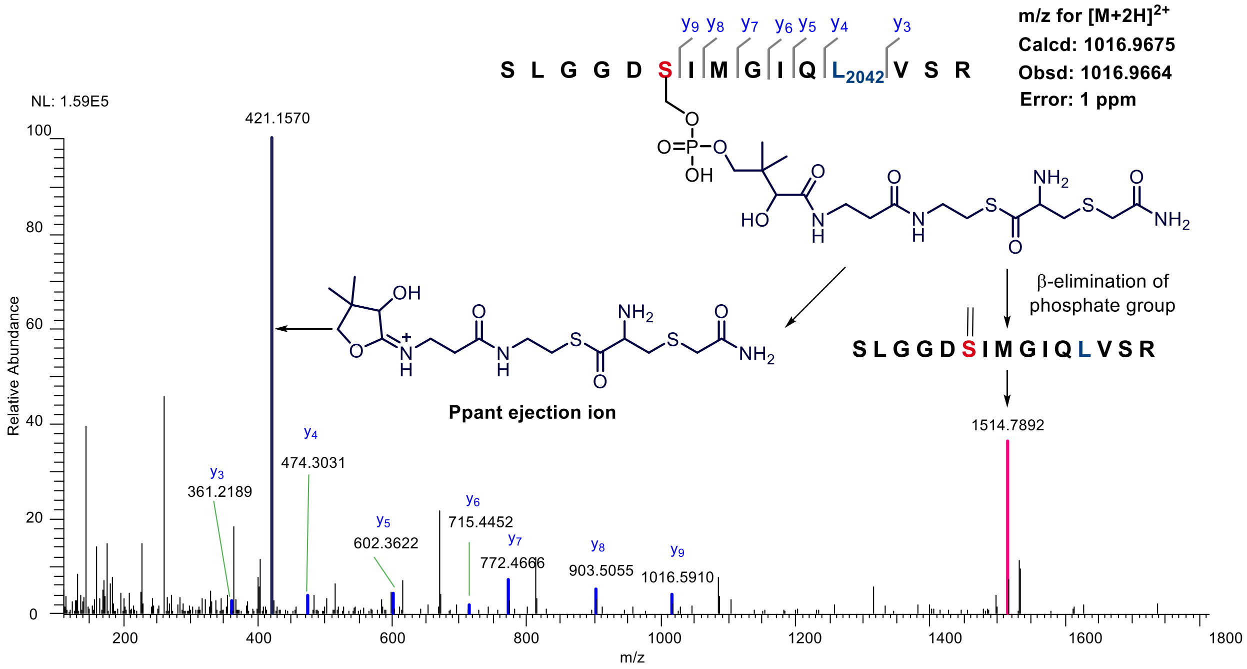

m/z for [M+2H]2+
Calcd: 1016.9675
Obsd: 1016.9664
Error: 1 ppm
S L G G D S I M G I Q L2042 V S R
y9 y8 y7 y6 y5 y4 y3
β-elimination of phosphate group
SLGGDSIMGIQLVSR
Ppant ejection ion
NL: 1.59E5
421.1570
474.3031
361.2189
602.3622
715.4452
772.4666
903.5055
1016.5910
1514.7892
Relative Abundance
m/z

ii

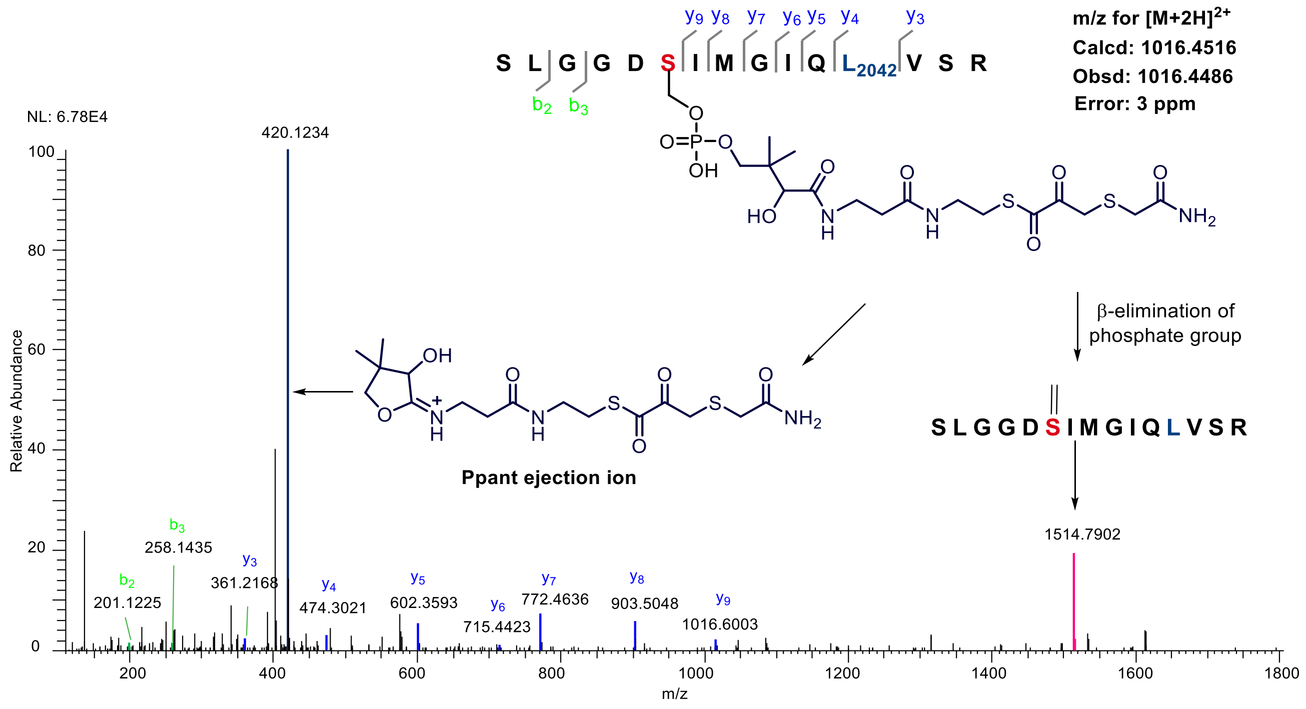

Supplementary Figure 7. Characterization of (3-sulfhydryl)-pyruvoyl-*S*-CaeA2^{F2042L} (**6**) by nanoLC-MS/MS following treatment with IAA and subsequent complete digestion with trypsin and chymotrypsin.(**a**) Incubation of thiolated CaeA2^{F2042L}, CaeB1 and L-cysteine. MS-detectable products include **4** (i) and **5** (ii).

b

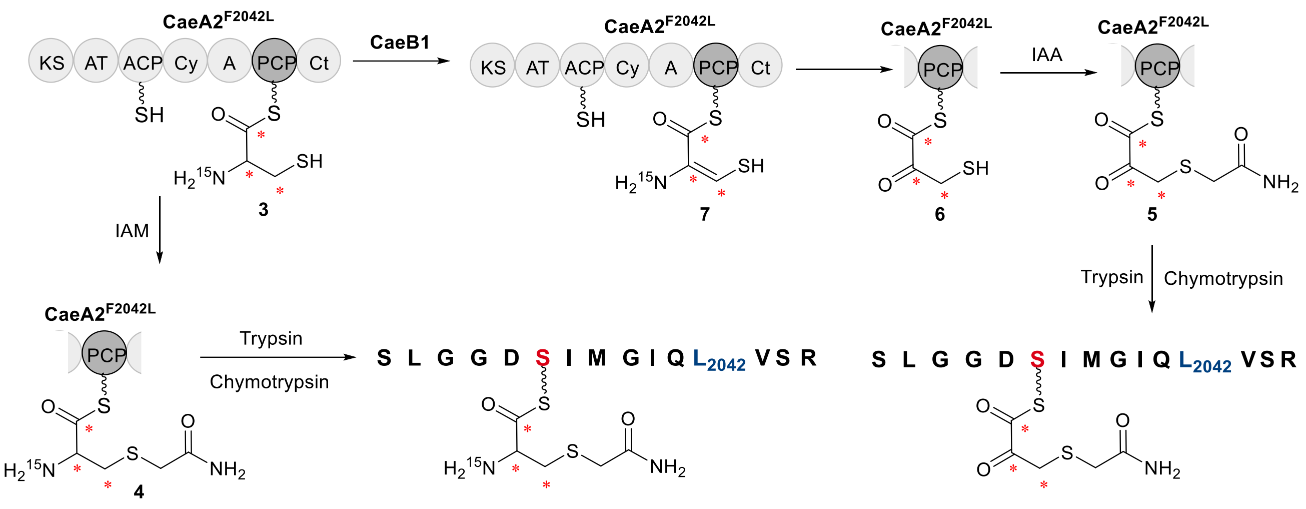
CaeA2F2042L
KS AT ACP Cy A PCP Ct
CaeB1
IAA
IAM
Trypsin
Chymotrypsin
3
7
6
5
4
S L G G D S I M G I Q L2042 V S R

i

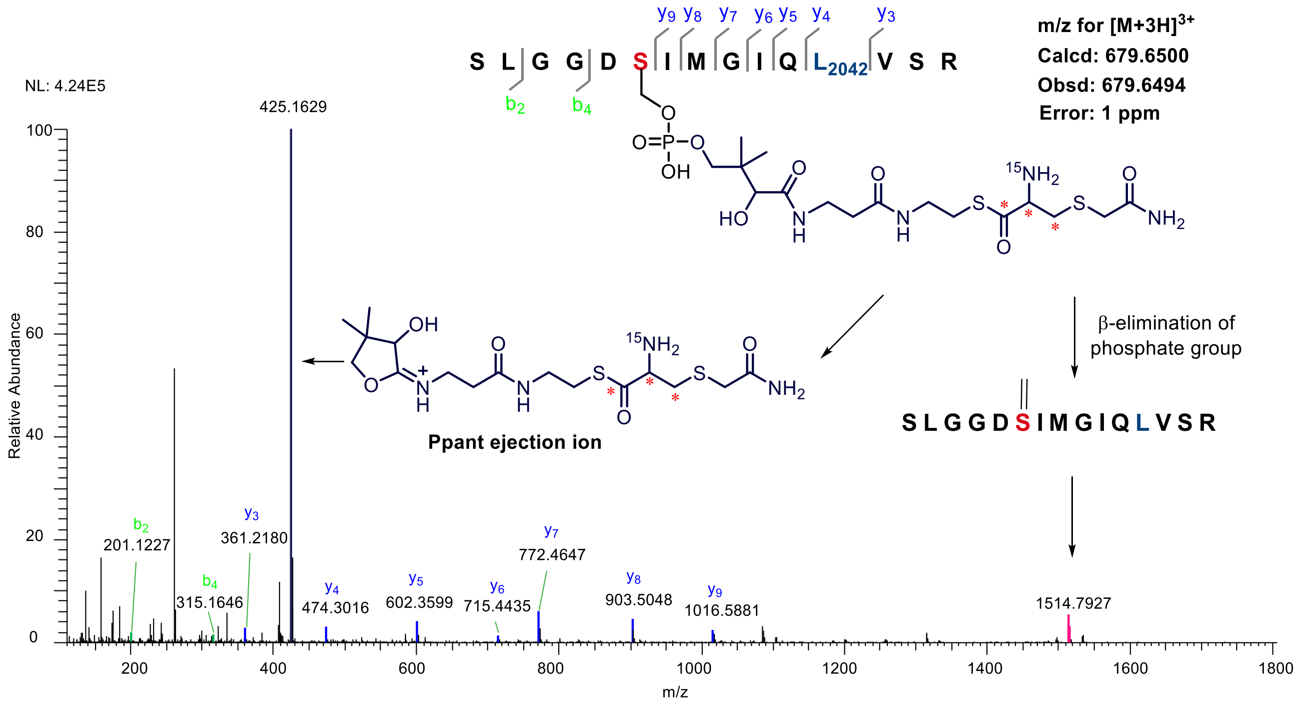
NL: 4.24E5
425.1629
m/z for [M+3H]3+
Calcd: 679.6500
Obsd: 679.6494
Error: 1 ppm
S L G G D S I M G I Q L2042 V S R
Ppant ejection ion
β-elimination of phosphate group
S L G G D S I M G I Q L V S R
Relative Abundance
m/z
201.1227
315.1646
361.2180
474.3016
602.3599
715.4435
772.4647
903.5048
1016.5881
1514.7927

ii

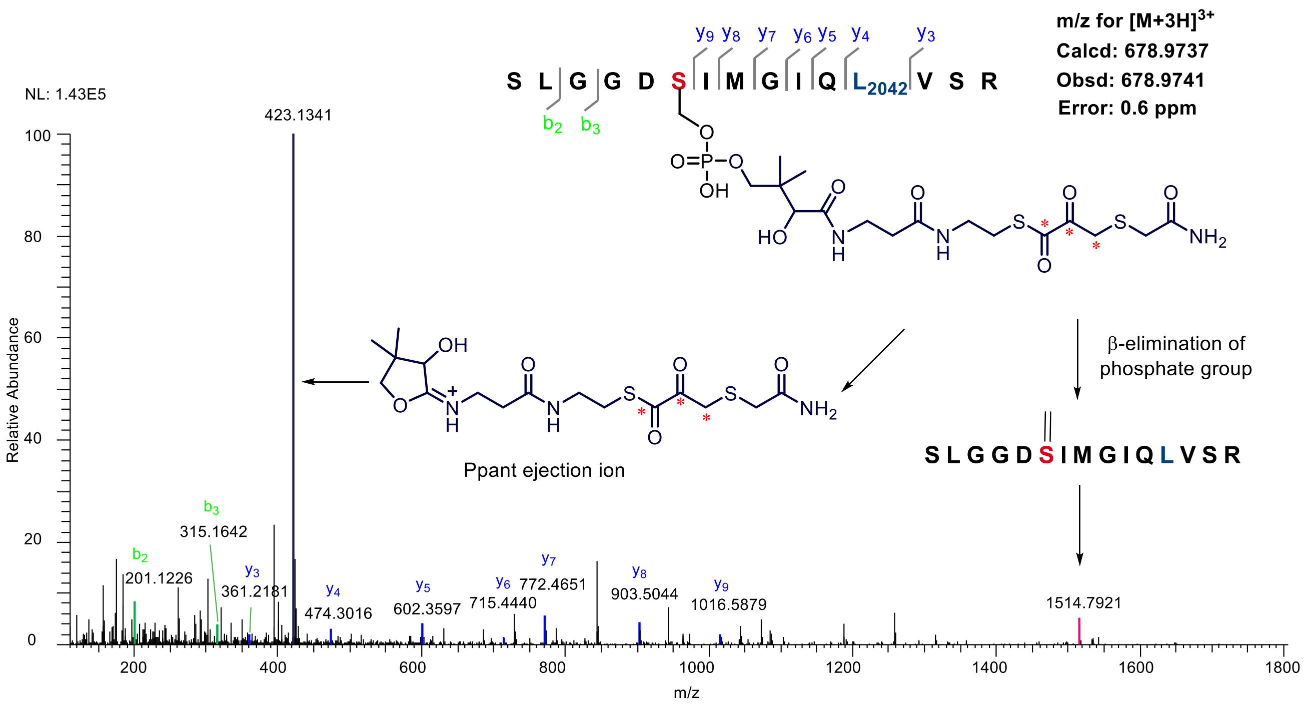

(**b**) Incubation of thiolated CaeA2^{F2042L}, CaeB1 and L-[1,2,3-$^{13}C_3$,^{15}N]cysteine. MS-detectable products include ^{15}N and/or ^{13}C labelled **4** (i) and **5** (ii).

c

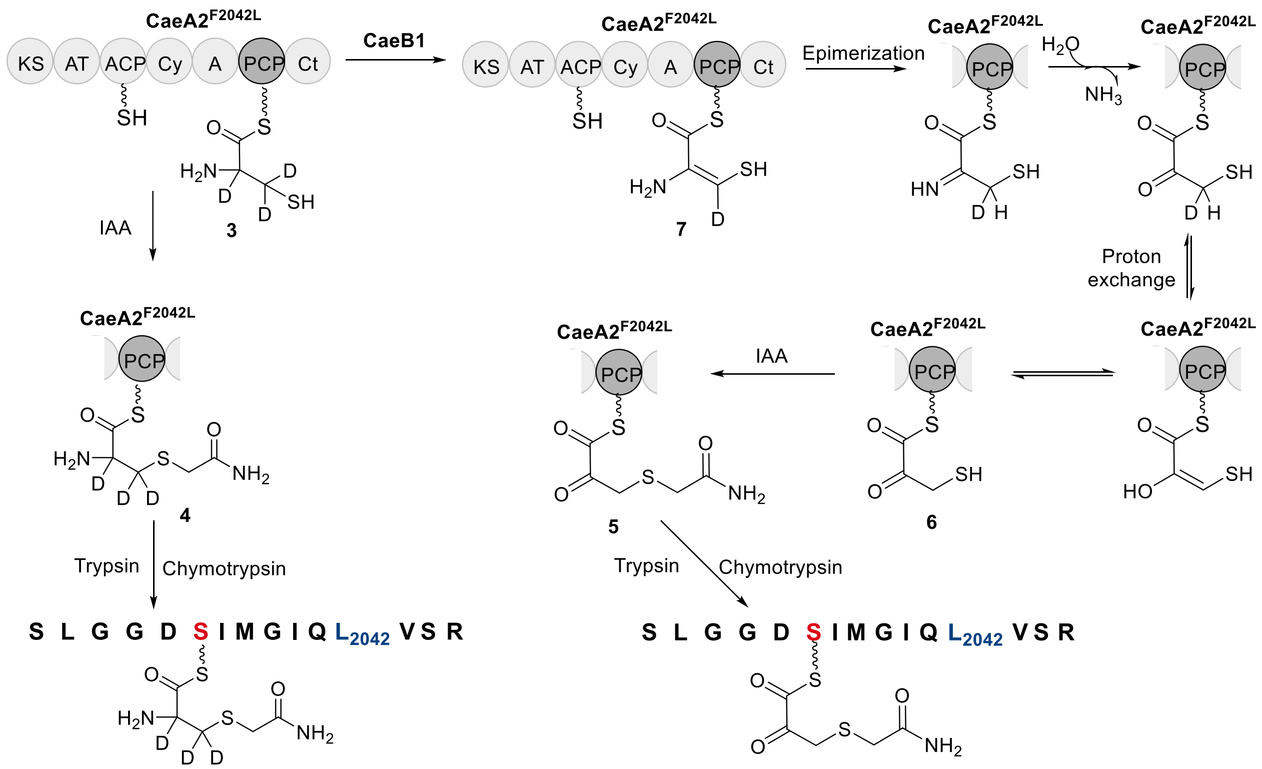

CaeA2F2042L
KS AT ACP Cy A PCP Ct
CaeB1
Epimerization
H2O
NH3
Proton exchange
IAA
Trypsin
Chymotrypsin
3
4
5
6
7
S L G G D S I M G I Q L2042 V S R

i

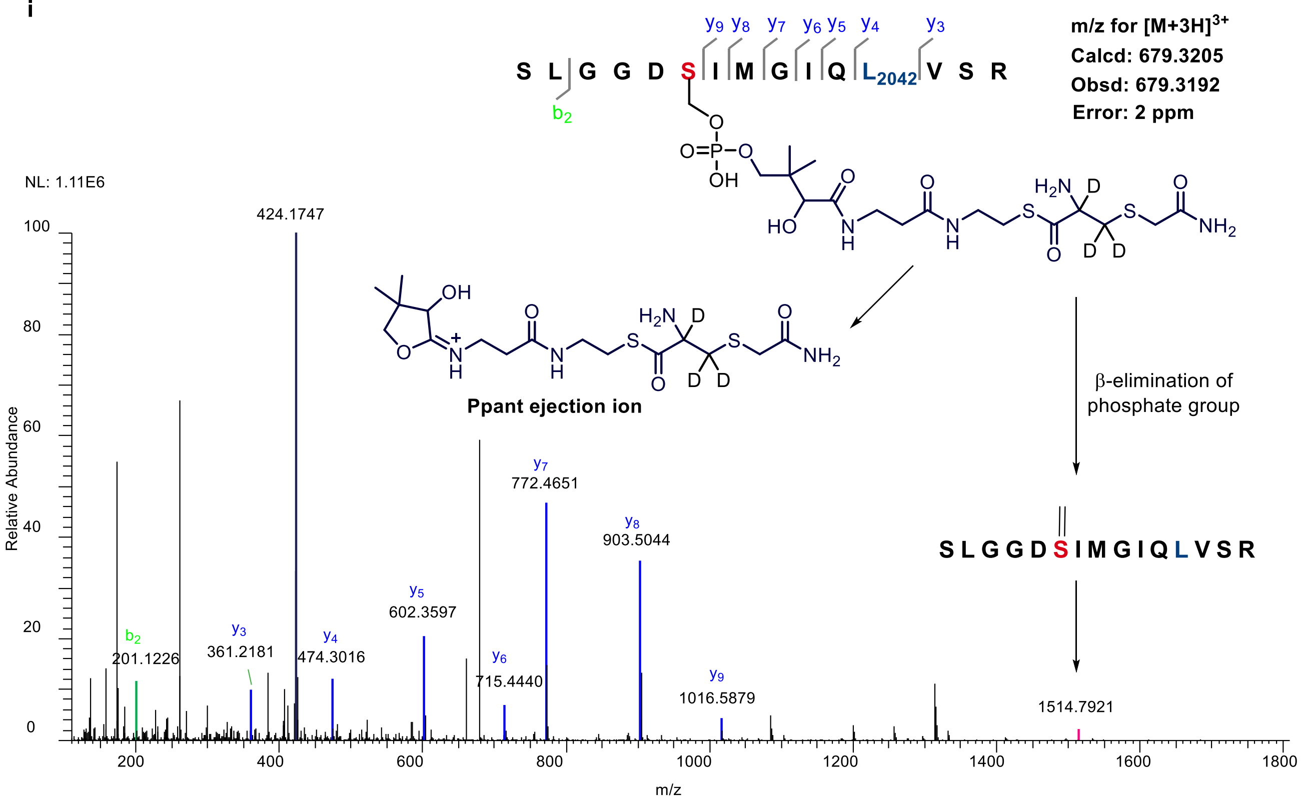

m/z for [M+3H]3+
Calcd: 679.3205
Obsd: 679.3192
Error: 2 ppm
S L G G D S I M G I Q L2042 V S R
y9 y8 y7 y6 y5 y4 y3
b2
Ppant ejection ion
β-elimination of phosphate group
SLGGDSIMGIQLVSR
NL: 1.11E6
Relative Abundance
m/z
424.1747
201.1226
361.2181
474.3016
602.3597
715.4440
772.4651
903.5044
1016.5879
1514.7921

ii

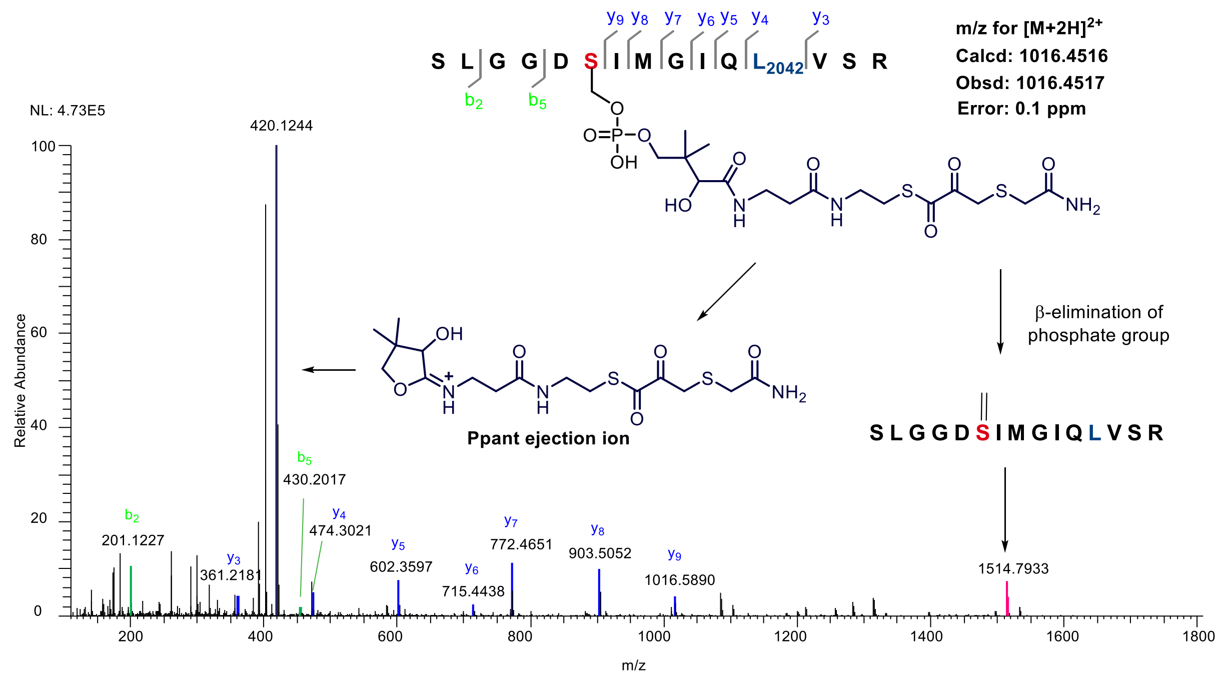

(**c**) Incubation of thiolated CaeA2^{F2042L}, CaeB1 and L-[2,3,3-D_3]cysteine. MS-detectable products include labelled **4** (i) and unlabeled **5** (ii).

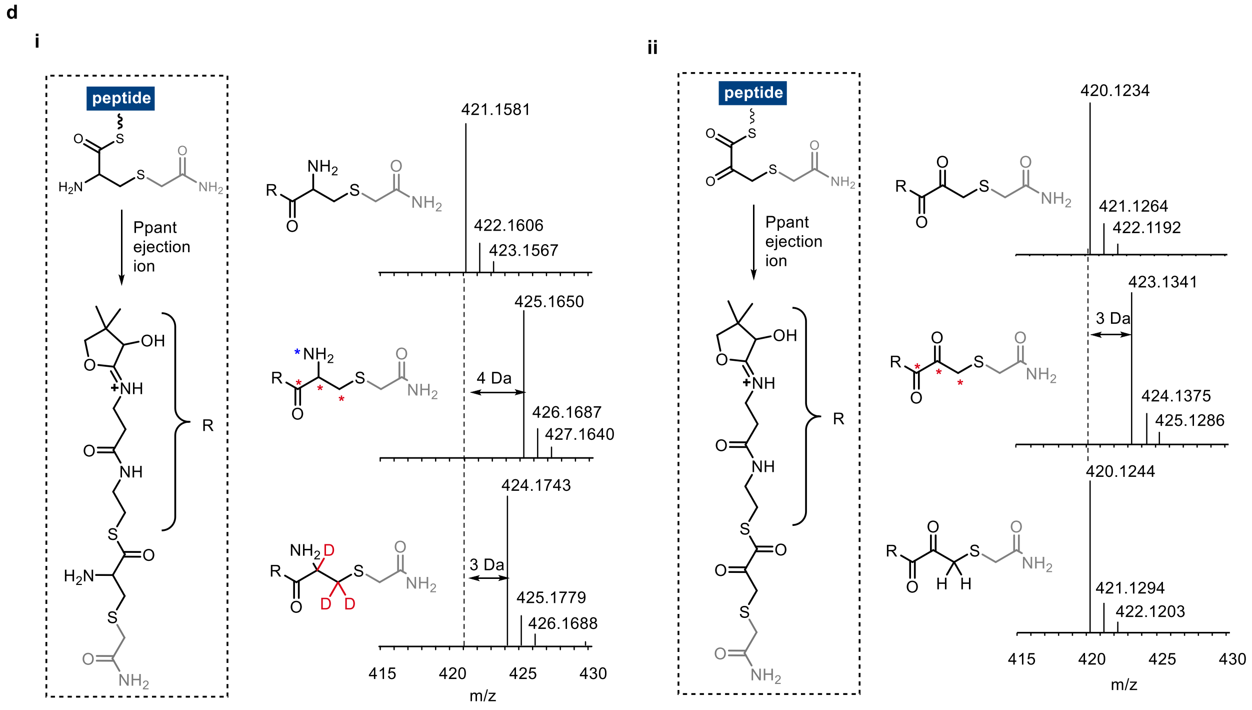

(**d**) MS comparison of **4** (i) and **5** (ii) in their associated Ppant ejection products, using L-cysteine (top), L-[1,2,3-$^{13}C_3$,^{15}N]cysteine (middle) and L-[2,3,3-D_3]cysteine (down) as the substrates, respectively.

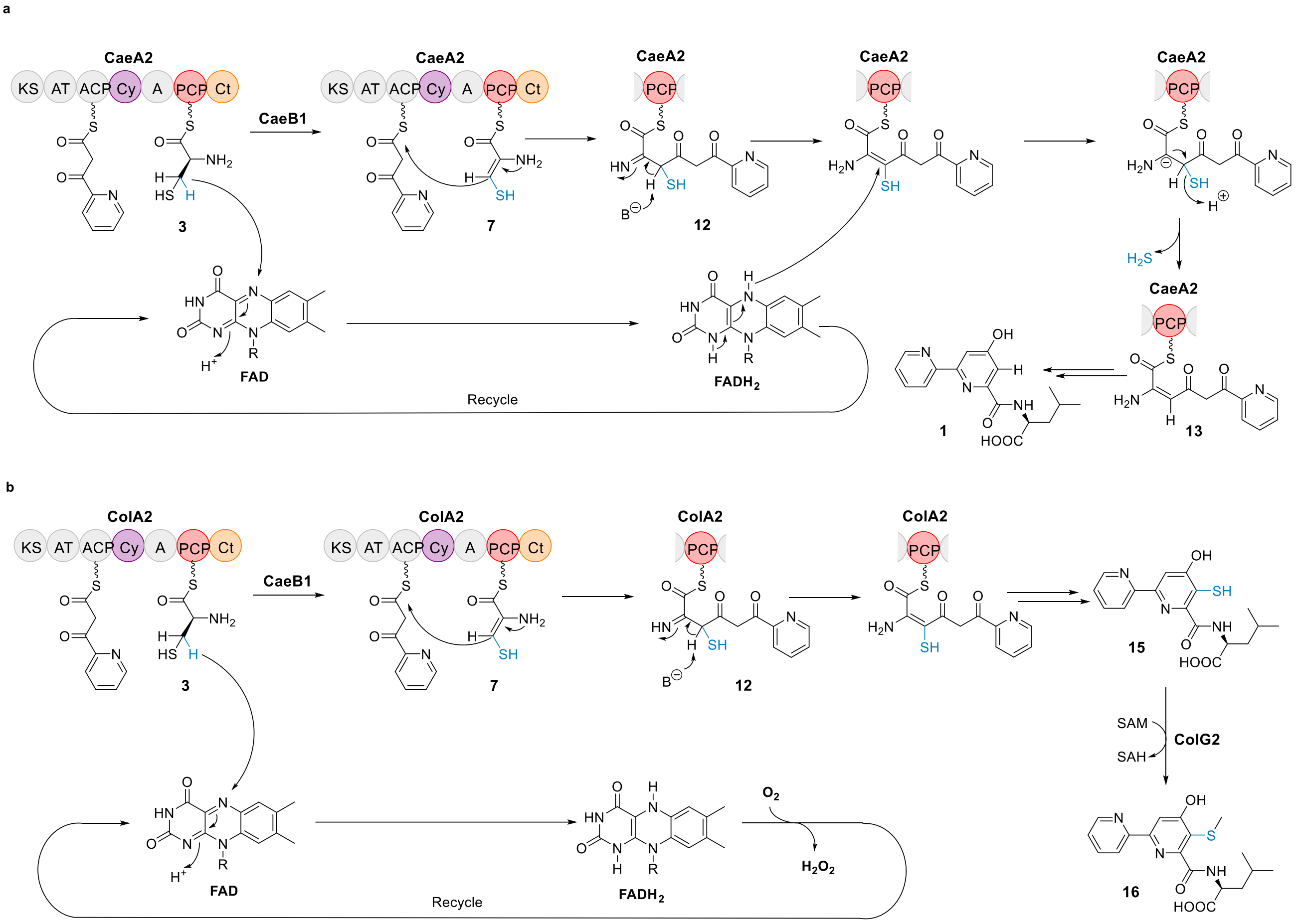

Supplementary Figure 8. Proposed mechanisms for flavin redox recycle in the CAEs (**a**) and COLs (**b**) biosynthetic pathways, respectively.

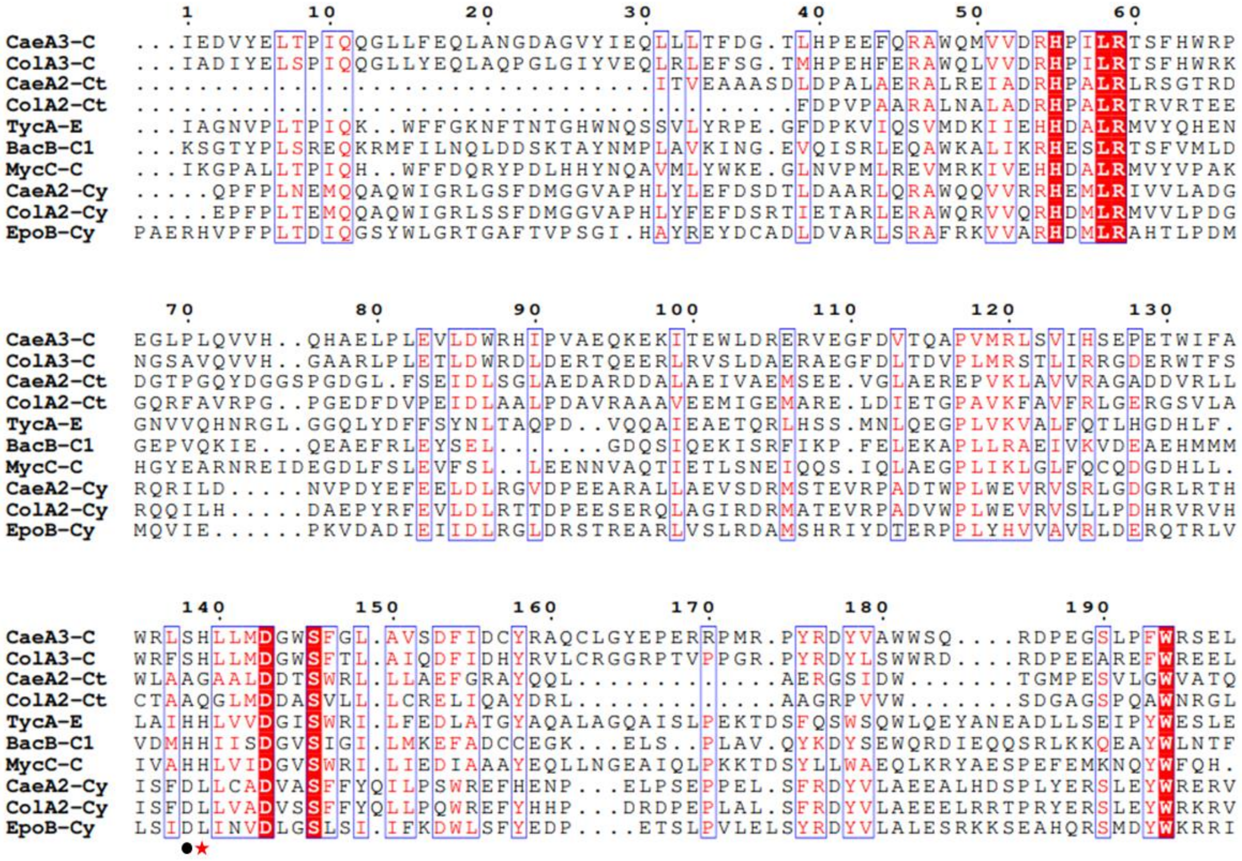

Supplementary Figure 9. Sequence analysis of the C domains of CaeA3 and ColA3, and the Cy and Ct domains of CaeA2 and ColA2 For comparison, the homologs include the epimerization (E) domain of the NRPS TycA (610-913 aa, AAC45928.1) in tyrocidine biosynthesis[12], the first C domain of the NRPS BacB (72-361 aa, AAC06347.1) in bacitracin biosynthesis[13], the C domain of the NRPS MycC (855-1156 aa, AAF08797.1) in mycosubtilin biosynthesis[14], and the Cy domain of the NRPS EpoB (71-364 aa, ADB12489.1) in epothilone biosynthesis[15]. The boundaries of each domain were determined by Pfam. The active site residue L-histine of C domains is indicated by red star, and the active site residue L-glutamic acid of Cy domains is indicated by black dot.

a

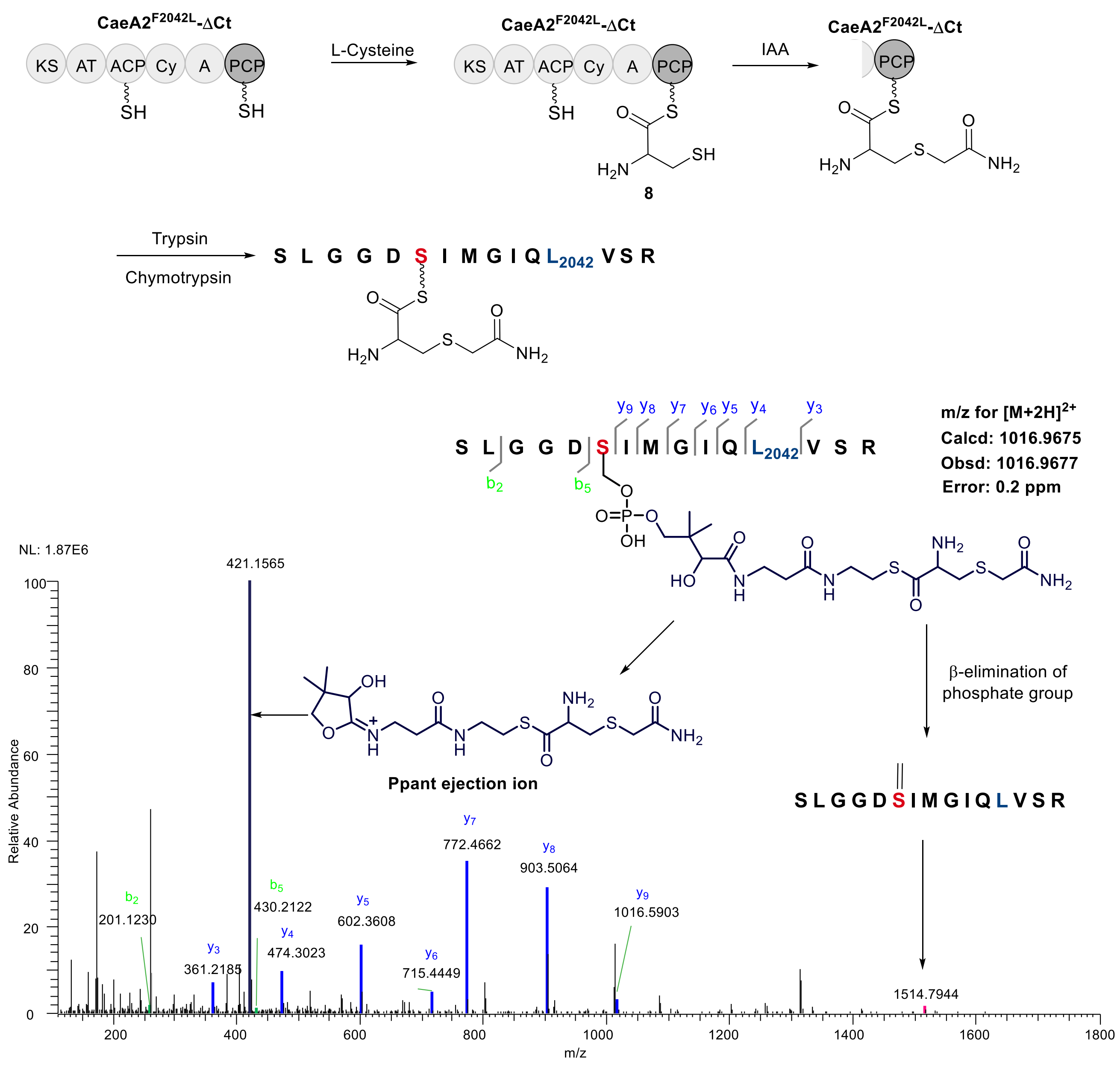

Supplementary Figure 10. Characterization of L-Cysteinyl-*S*-CaeA2^{F2042L}△Ct (**8**) by nanoLC-MS/MS following treatment with IAA and subsequent complete digestion with trypsin and chymotrypsin. (**a**) Incubation of truncated CaeA2^{F2042L}△Ct and L-cysteine.

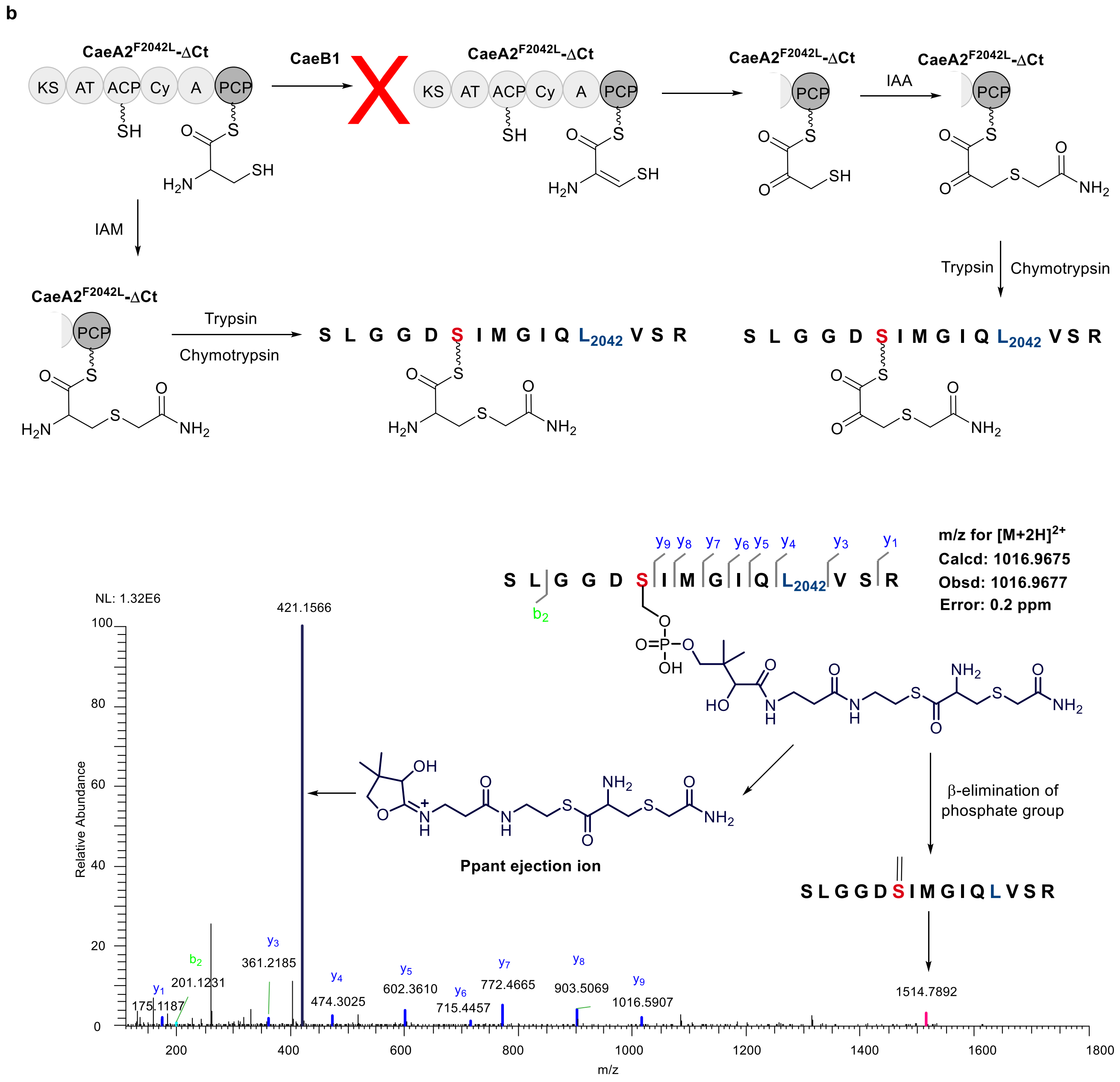

(**b**) Incubation of CaeB1 with truncated CaeA2^{F2042L}△Ct and L-cysteine.

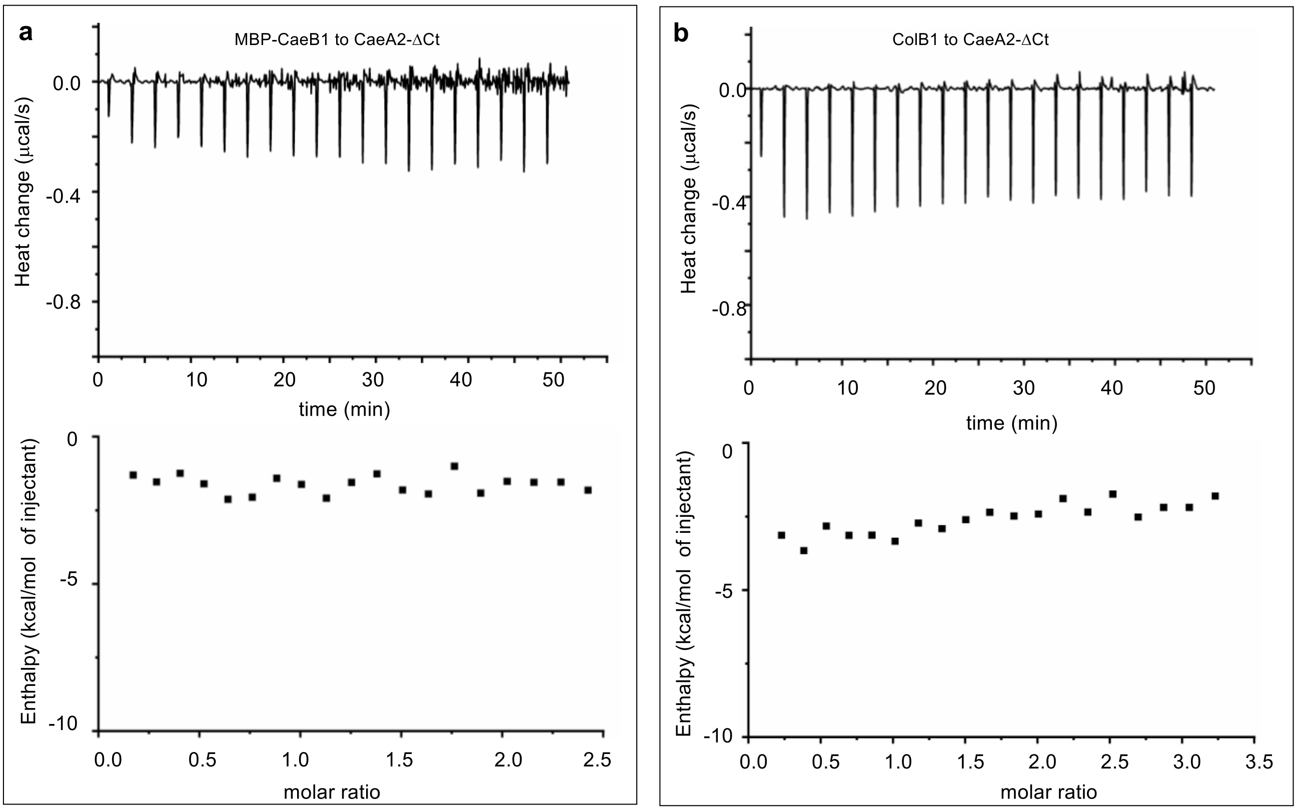

Supplementary Figure 11. Measurement of the interactions of truncated CaeA2-ΔCt with related flavoproteins by ITC. Raw data were shown on top, and the integrated curves containing experimental points and the best fitting line obtained from the single binding site model were shown on bottom. (**a**) Titrating MBP-fused CaeB1 to CaeA2-ΔCt. (**b**) Titrating ColB1 to CaeA2-ΔCt.

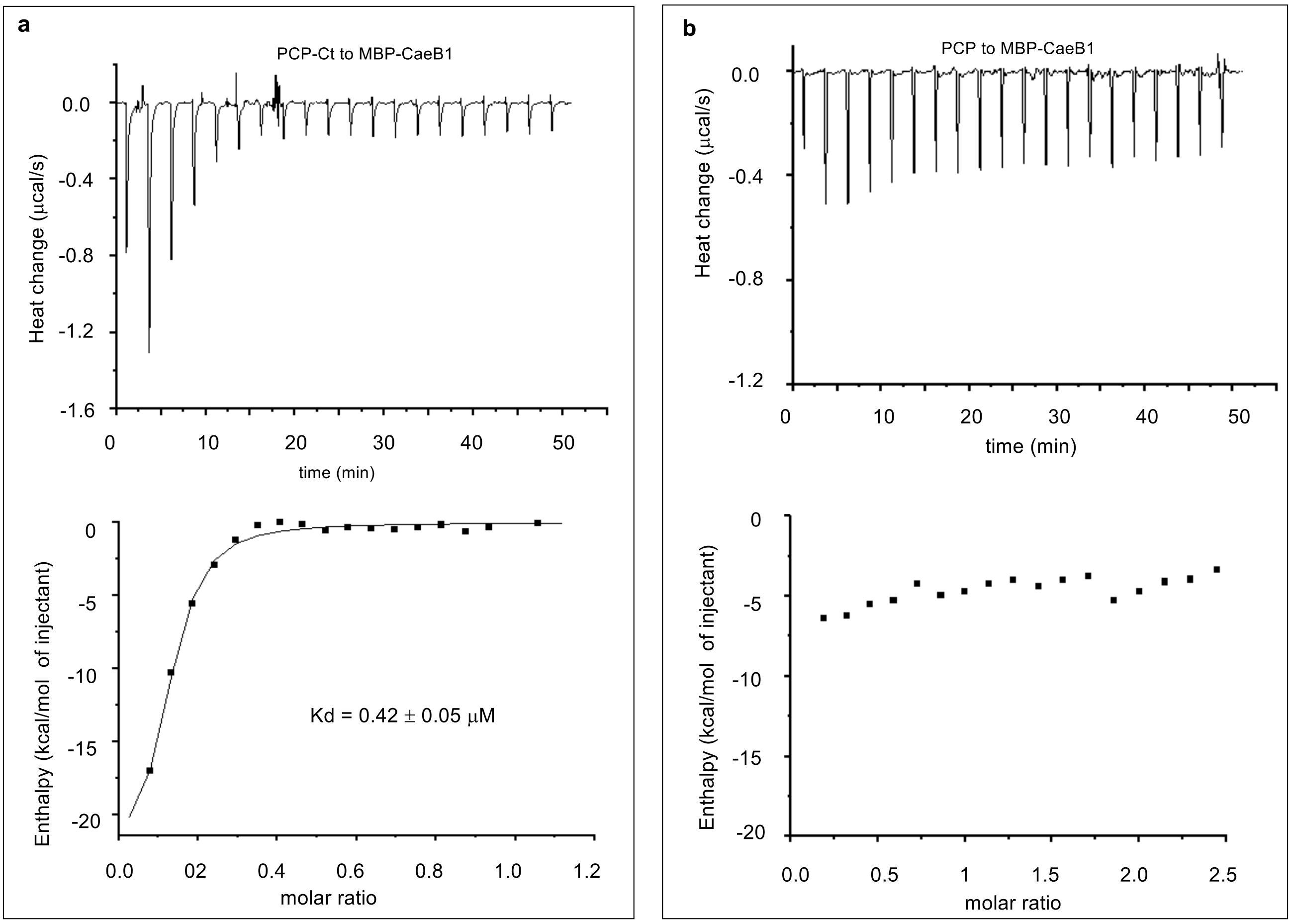

Supplementary Figure 12. Measurement of the interactions of PCP-Ct$_{\mathrm{CaeA2}}$ and PCP $_{\mathrm{CaeA2}}$ with related flavoproteins by ITC. Raw data were shown on top, and the integrated curves containing experimental points and the best fitting line obtained from the single binding site model were shown on bottom. (**a**) Titrating MBP-fused CaeB1 to Sumo-PCP-Ct$_{\mathrm{CaeA2}}$. (**b**) Titrating MBP-fused CaeB1 to PCP$_{\mathrm{CaeA2}}$.

a

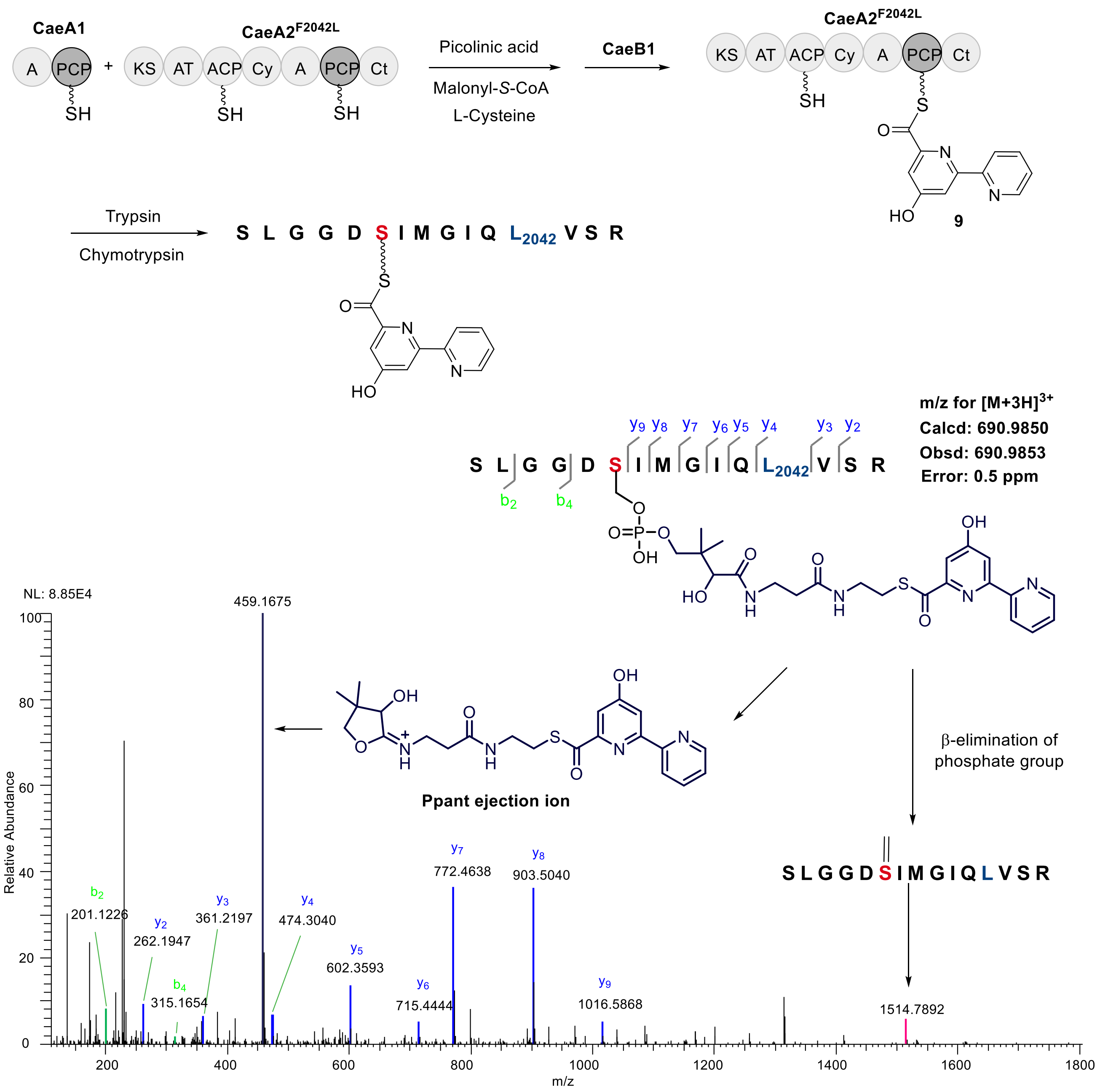

Supplementary Figure 13. Characterization of 2,2'-bipyridinyl-*S*-CaeA2^{F2042L} (**9**) by nanoLC-MS/MS following complete digestion with trypsin and chymotrypsin. (**a**) Incubation of CaeA1, CaeA2^{F2042L} and CaeB1 with picolinic acid, malonyl-*S*-CoA and L-cysteine.

b

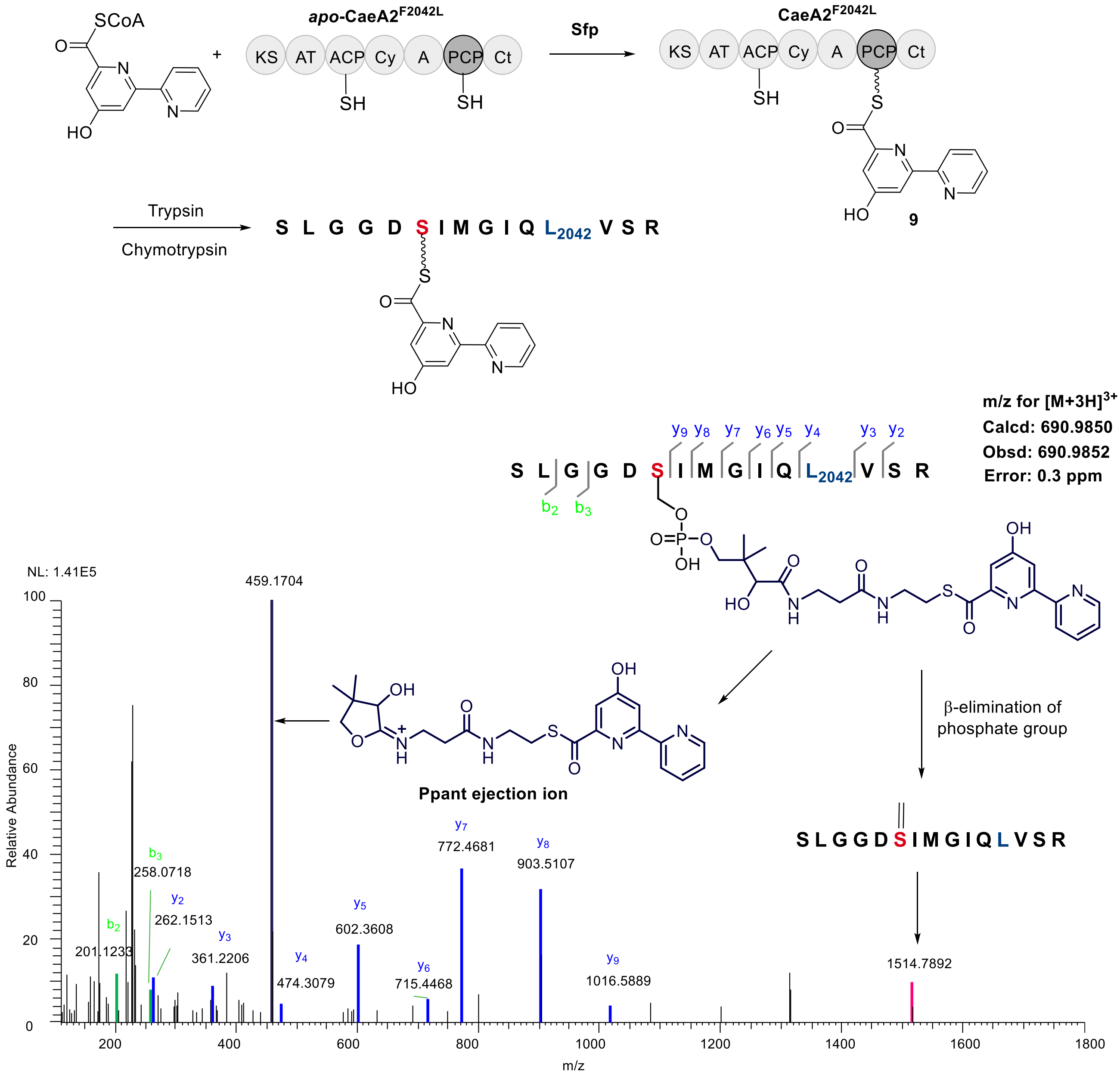

(**b**) Incubation of Ppant-unmodified CaeA2^{F2042L} (produced in *E. coli* BL21(DE3)) with synthesized 2,2'-bipyridinyl-*S*-CoA in the presence of Sfp.

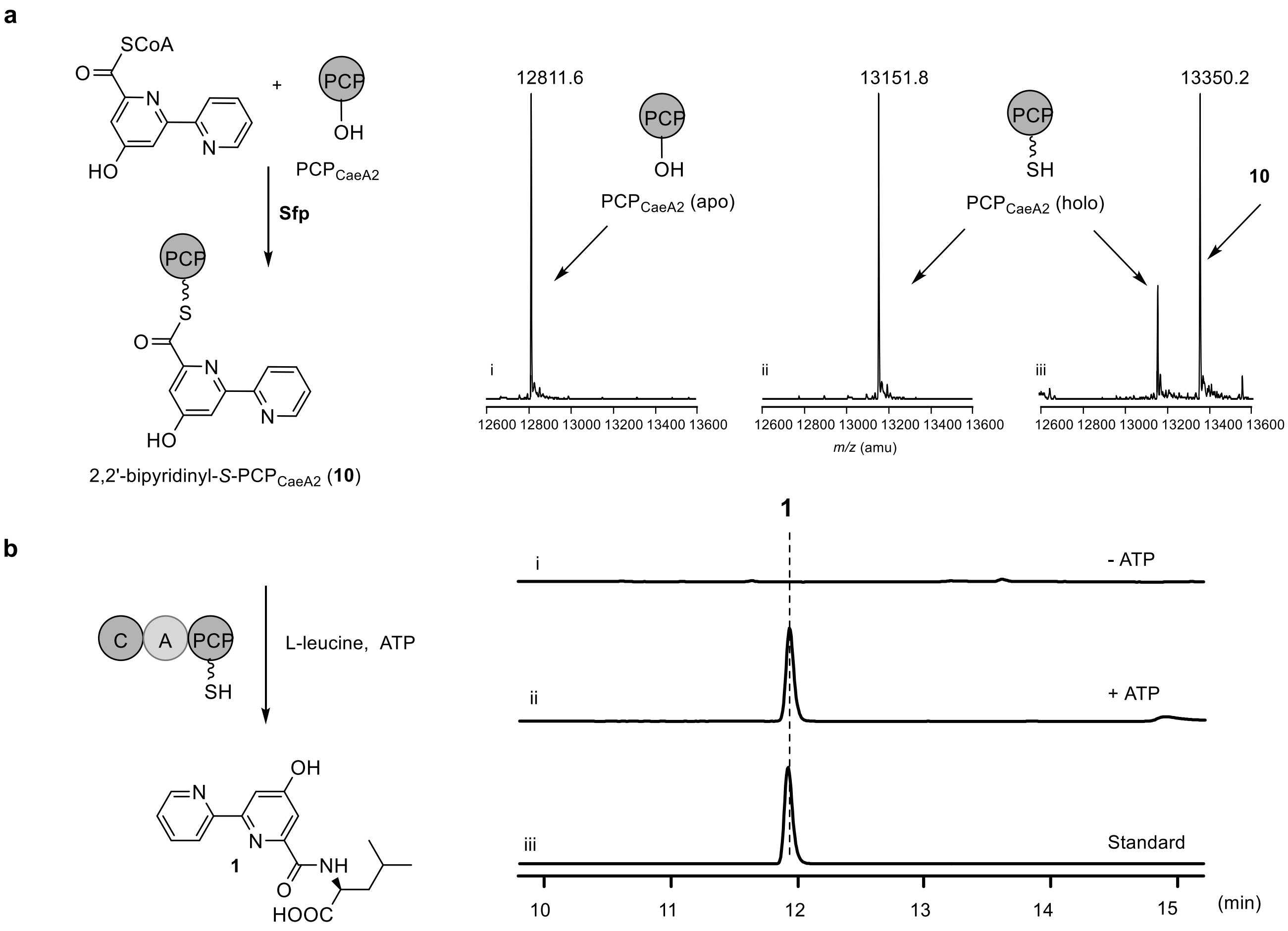

Supplementary Figure 14. Validation of 2,2'-bipyridinyl-*S*-Ppant as an intermediate in the production of **1**. (**a**) Preparation of 2,2'-bipyridinyl-*S*-PCP$_{CaeA2}$ (**10**) by the incubation of PCP$_{CaeA2}$ in apo form (produced in *E. coli* BL21(DE3)) with synthesized 2,2'-bipyridinyl-*S*-CoA in the presence of Sfp. PCP$_{CaeA2}$ in both apo (i) and holo (ii) forms and 2,2'-bipyridinyl-*S*-PCP$_{CaeA2}$ (iii) were examined by HR-MS. (**b**) Incubation of prepared 2,2'-bipyridinyl-*S*-PCP$_{CaeA2}$ with CaeA3 and L-leucine in the absence (i) and presence (ii) of ATP， the standard of **1** (iii).

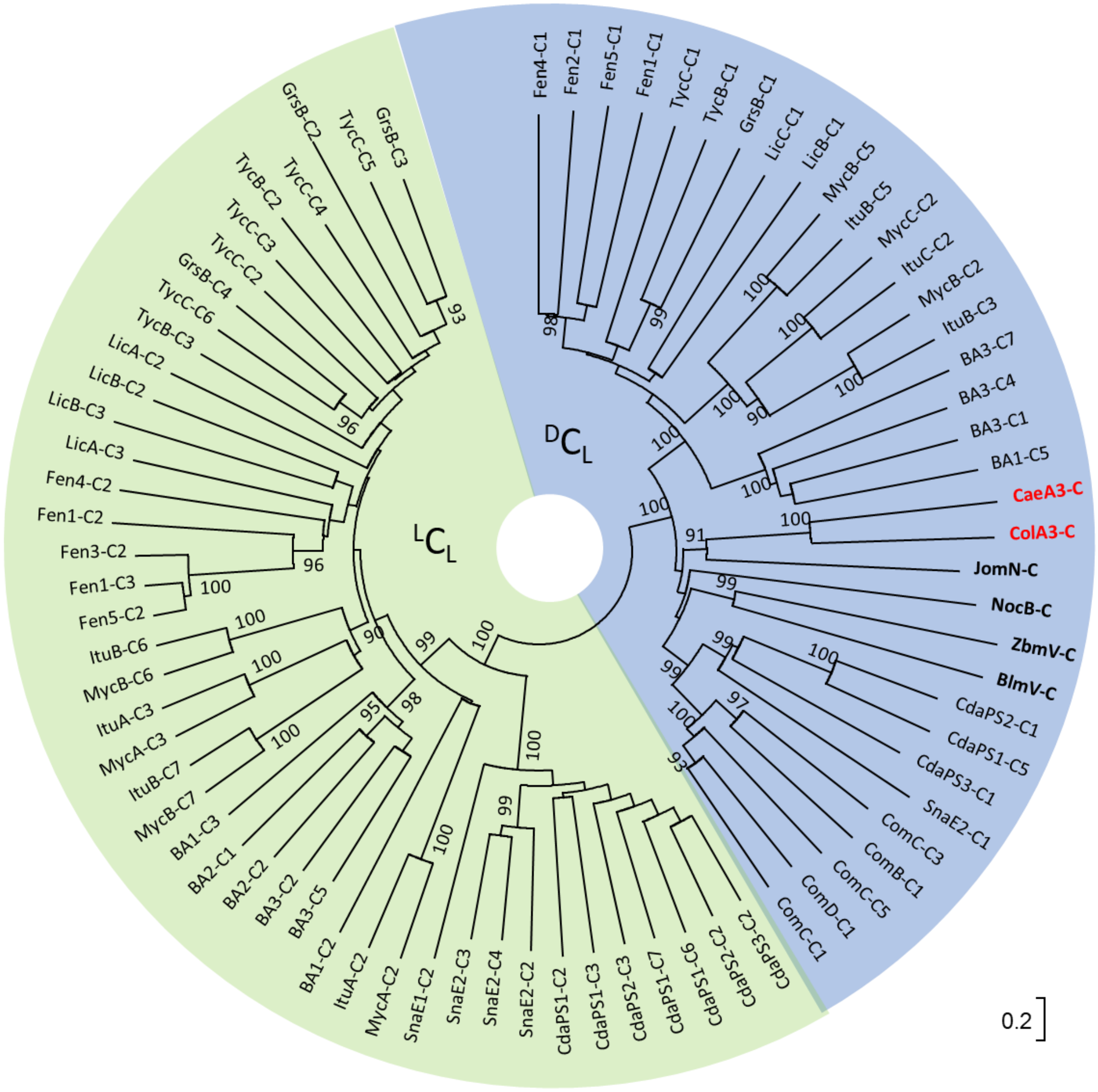

Supplementary Figure 15. Phylogenetic analysis of the C domains of NRPS CaeA3 and ColA3 in substrate stereo-chemistry. The evolutionary distances were computed using the p-distance method. The support for grouping the clades $^{L}C_{L}$ (green) and $^{D}C_{L}$(blue) is indicated by bootstrap value. The C domains of CaeA3 and ColA3 are shown in red and bold, the C domain of NocB that mediates β-lactam formation in nocardicin biosynthesis[16] and the C domains of JomN, BlmV and ZbmV in jomthonic acids[46], bleomycins[31] and zorbamycin[47] biosynthesis, respectively, are shown in bold. The

homologous C domains arise from the NRPSs BA1, BA2 and BA3 in bacitracin biosynthesis[13]; CdaPS1, CdaPS2 and CdaPS3 in calcium-dependent antibiotic biosynthesis[17]; ComB, ComC and ComD in complestatin biosynthesis[18]; Fen1, Fen2, Fen3, Fen4 and Fen5 in fengycin bioysynthesis[19]; GrsB in gramicidin biosynthesis[20]; ItuA, ItuB and ItuC in iturin biosynthesis[21]; LicA, LicB and LicC in lichenicin biosynthesis[22]; MycA, MycB and MycC in mycosubtilin biosynthesis[23]; SnaE1 and SnaE2 in pristinamycin biosynthesis[24]; and TycB and TycC in tyrocidine biosynthesis[12]. The sequences were downloaded from the database of NaPDoS, in which the biochemical function and substrate stereo-chemistry of these related C domains have been confirmed[25,26].

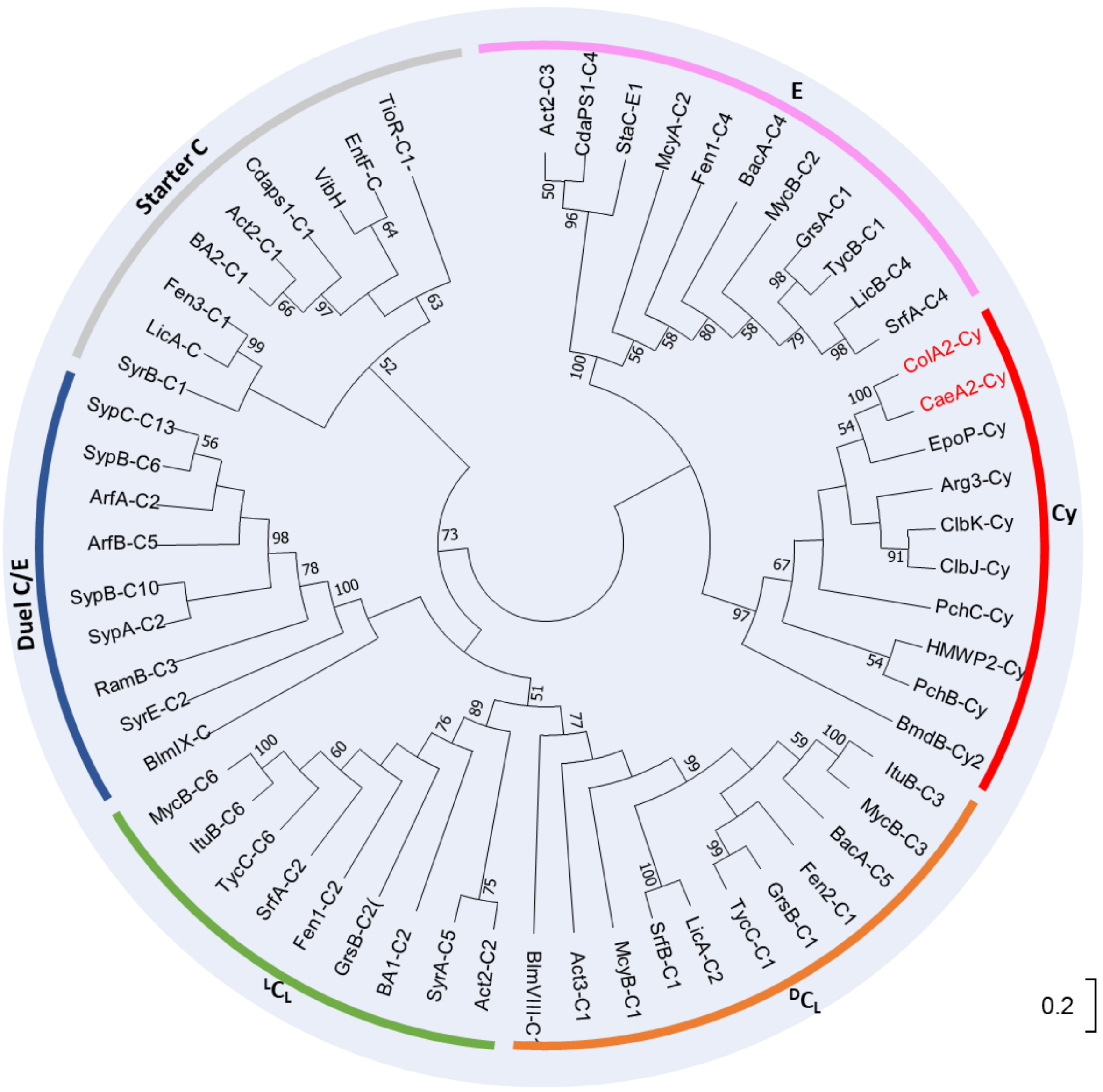

Supplementary Figure 16. Phylogenetic analysis of the Cy domains of the NRPSs CaeA2 and ColA2. The evolutionary distances were computed using the p-distance method. The support for grouping the C domain subtypes is indicated by bootstrap value. The Cy domains of CaeA2 and ColA2 are shown in red. Members of the C domain subtypes ($^{L}C_{L}$, $^{D}C_{L}$, Starter C, Epimerization (E), Duel E/C and Heterocyclization (Cy) domains) include those of the NRPSs Act2 and Act3 in actinomycin biosynthesis[27]; ArfA and ArfB in arthrofactin biosynthesis[28], Arg3 in argyrins biosynthesis[29]; BA1 and BA2 in bacitracin biosynthesis[13]; BmdB in bacillamide biosynthesis[30]; BlmVIII and BlmIV in bleomycin biosynthesis[31]; ClbJ and ClbK in colibactin biosynthesis[32]; CdaPS1 in calcium-dependent antibiotic biosynthesis[17]; EpoP in epothilone biosynthesis[15]; EntF in enterobactin biosynthesis[33]; Fen1,

Fen2 and Fen3 in fengycin bioysynthesis[19]; GrsA and GrsB in gramicidin biosynthesis[20]; HMWP2 in yersiniabactin biosynthesis[34]; ItuB in iturin biosynthesis[21]; LicA and LicB in lichenicin biosynthesis[22]; McyA and McyB in microcystin biosynthesis[35]; MycB in mycosubtilin biosynthesis[23]; PchB and PchC in pyochelin biosynthesis[36]; RamB in ramoplanin biosynthesis[37]; SrfA and SrfB in surfactin biosynthesis[38]; SypA, SypB and SypC in syringopeptin biosynthesis[39]; StaC in A47934 biosynthesis[40]; SyrA, SyrB and SyrE in syringomycin biosynthesis[41]; TycB and TycC in tyrocidin biosynthesis[12]; TioR in thiocoraline biosynthesis[42]; VibH in vibriobactin biosynthesis[43]. The sequences were downloaded from the database of NaPDoS, in which the biochemical function of these related C domains have been characterized[25,26].

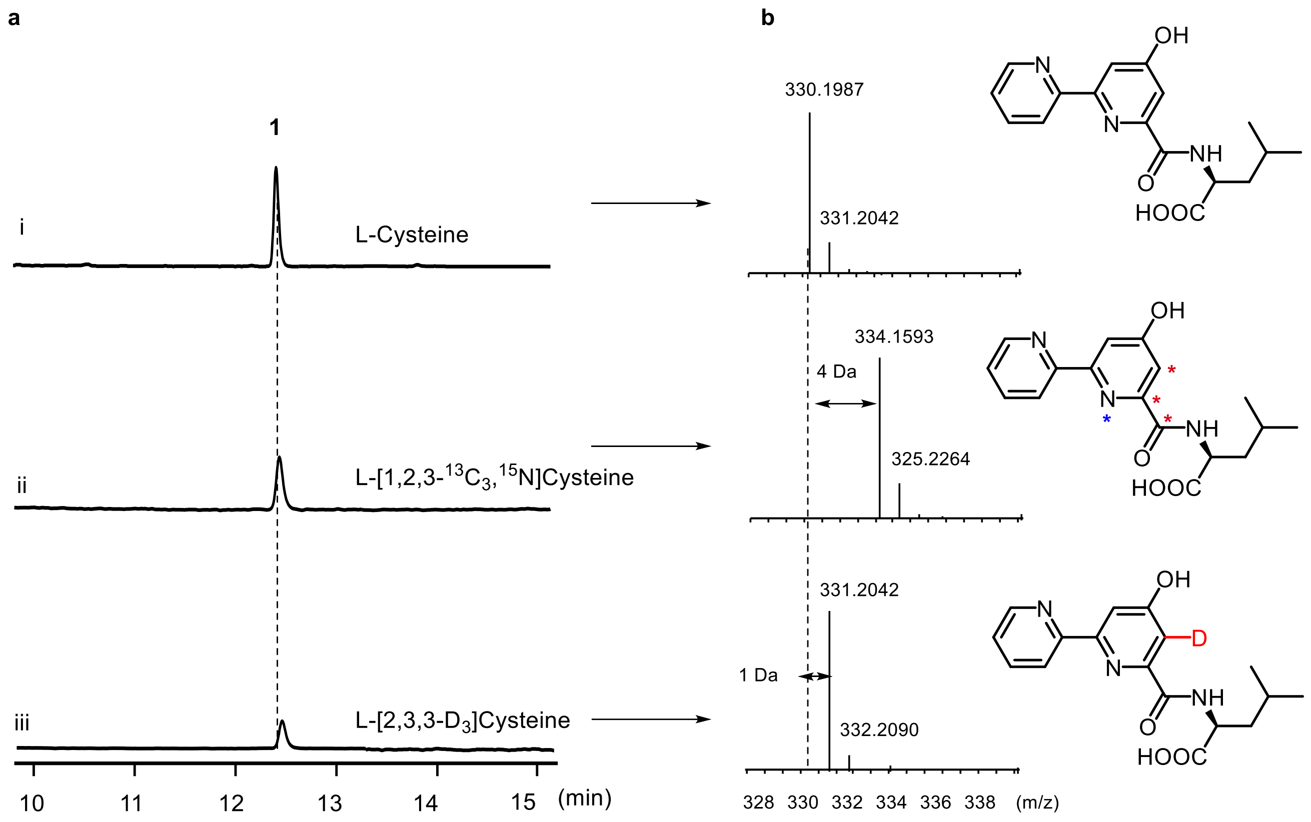

Supplementary Figure 17. Comparison in the production of the CAE 2,2'-bipyridine intermediate **1** using the substrate L-cysteine (i), L-[1,2,3-$^{13}C_3$,^{15}N]cysteine (ii) and L-[2,3,3-D_3]cysteine (iii), respectively. (**a**) HPLC analysis. (**b**) HR-MS analysis.

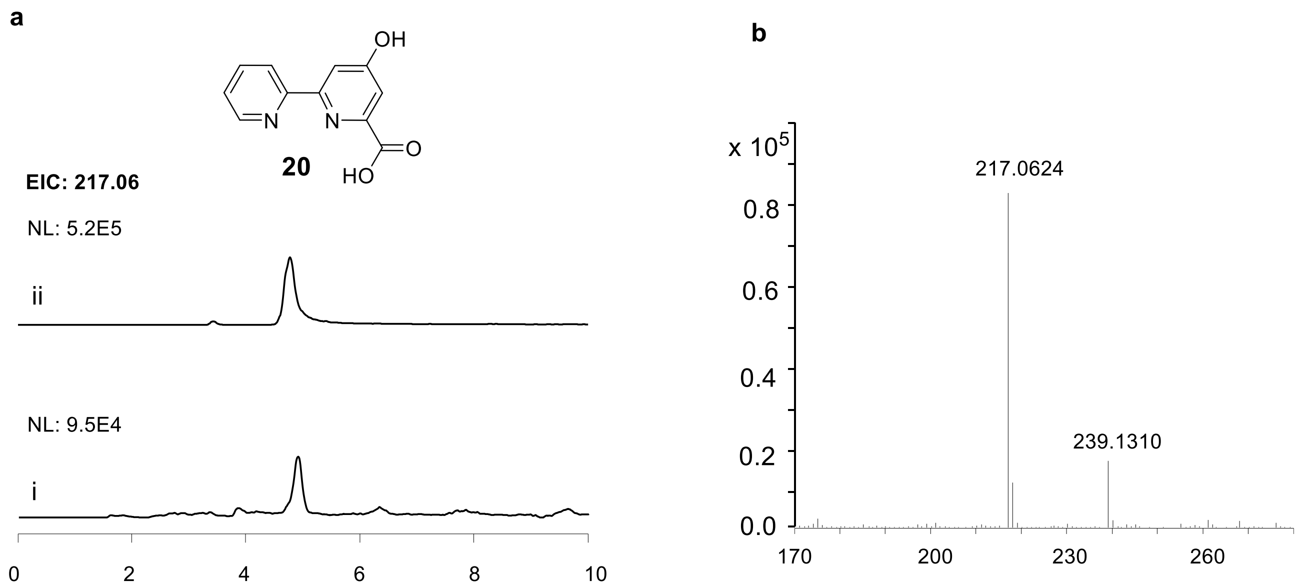

Supplementary Figure 18. Examination of dethiolated 2,2'-bipyridine carboxylate (**20**). (**a**) HPLC-MS analysis of the fermentation broth of the wild-type *S. roseosporus* strain, where sulfhydryl-2,2'-bipyridine antibiotics COLs are produced (i), with synthetic **20** as a standard (ii). (**b**) HR-MS analysis of **20** ($[M + H]^+$ *m/z*: cald. 217.0613 for $C_{11}H_9O_3N_2$, obs. 217.0624) observed in the wild-type *S. roseosporus* strain.

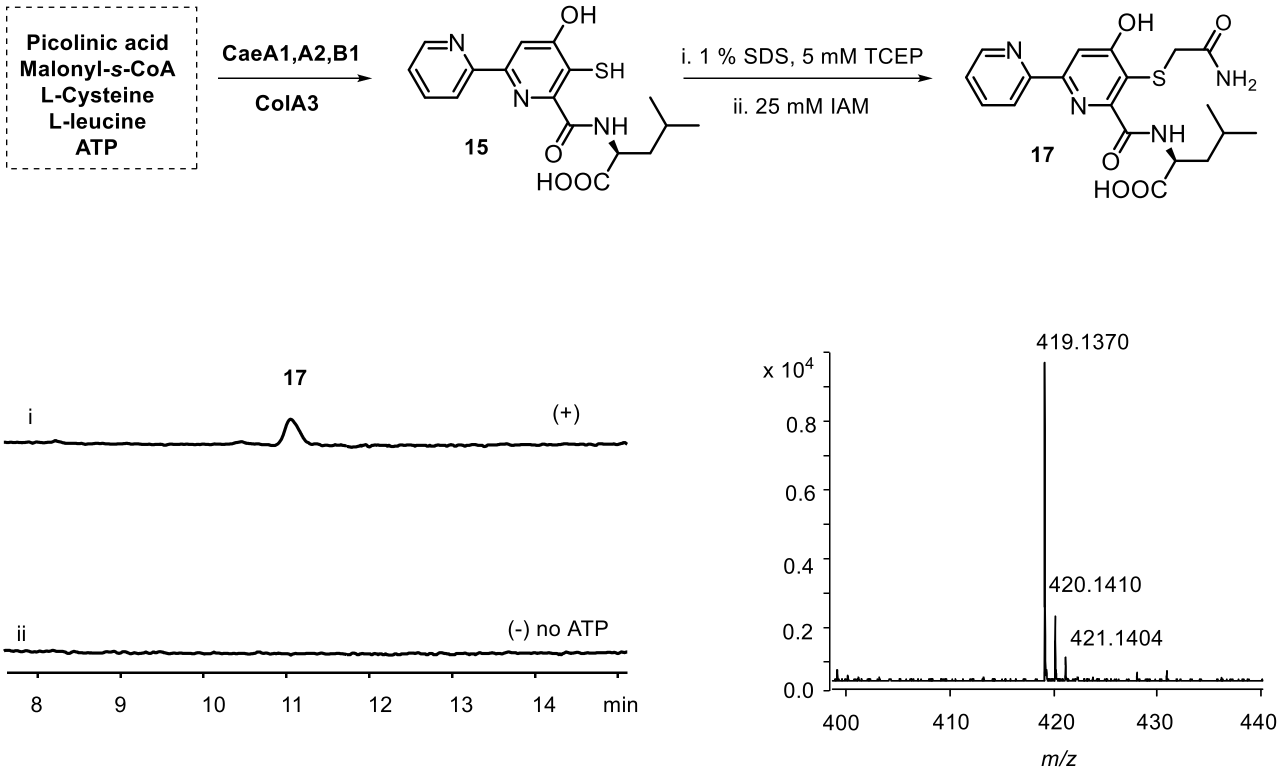

Supplementary Figure 19. Examination of thiol intermediate 5-sulfhydryl-2,2'-bipyridinyl-L-leucine (**15**) by IAA derivatization. **15** was produced in the reactions where CaeA1, CaeA2, CaeB1and ColA3 were combined with picolinic acid, malonyl-*S*-CoA, L-cysteine and L-leucine in the presence (i) or absence (ii) of ATP. The reaction mixtures were treated with IAA to produce **17**, which were then analyzed by HPLC (left, λ = 315 nm) and HR-MS (right, $[M + H]^+$ *m/z*: cald. 419.1389 for $C_{19}H_{22}O_5N_4S$, obs. 419.1370).

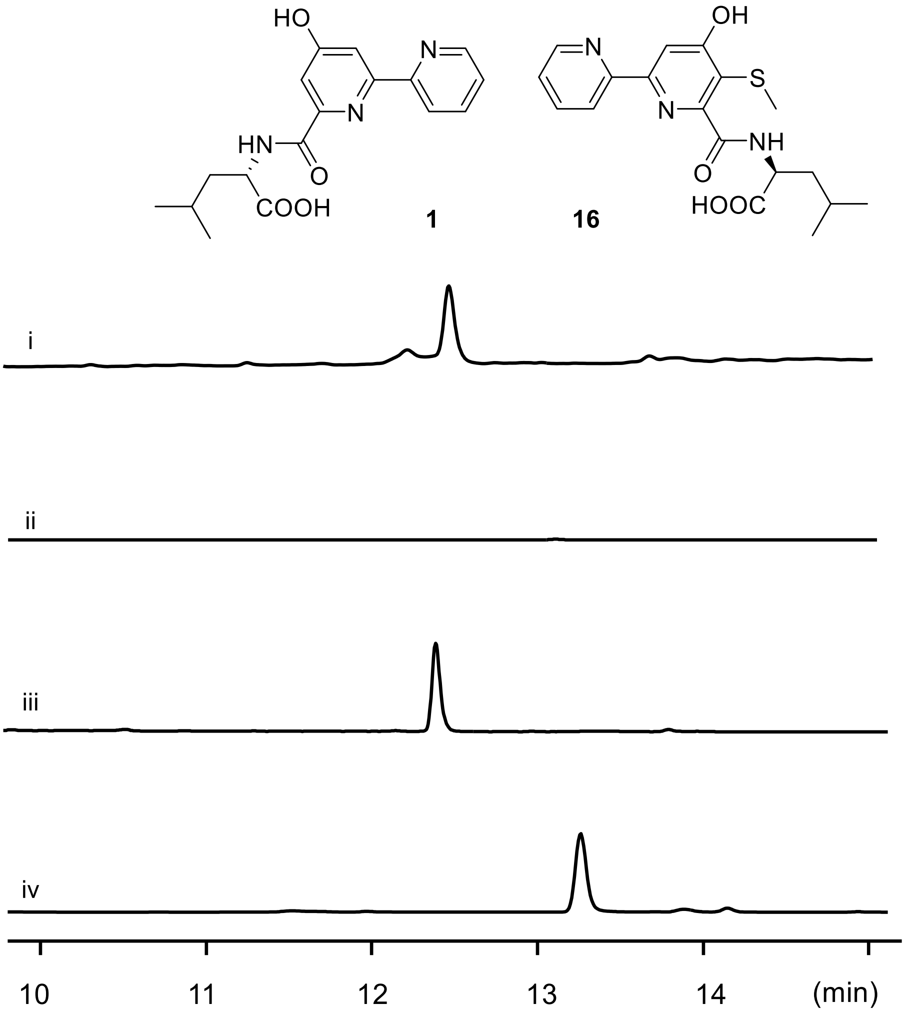

Supplementary Figure 20. Determination of the O2-dependence of CAE or COL biosynthesis by examining the production of **1** or **16** *in vitro* under anaerobic conditions. For **1** production (i), the reaction was conducted by combining the proteins CaeA1, CaeA2, CaeA3 and CaeB1 with the substrates picolinic acid, malonyl-*S*-CoA, L-cysteine, L-leucine, and ATP in the absence of O_2. For **16** production (ii), CaeA3 was replaced with ColA3 and ColG2 and SAM were added into the above reaction mixture. Synthetic **1** (iii) and **16** (iv) were used as standards.

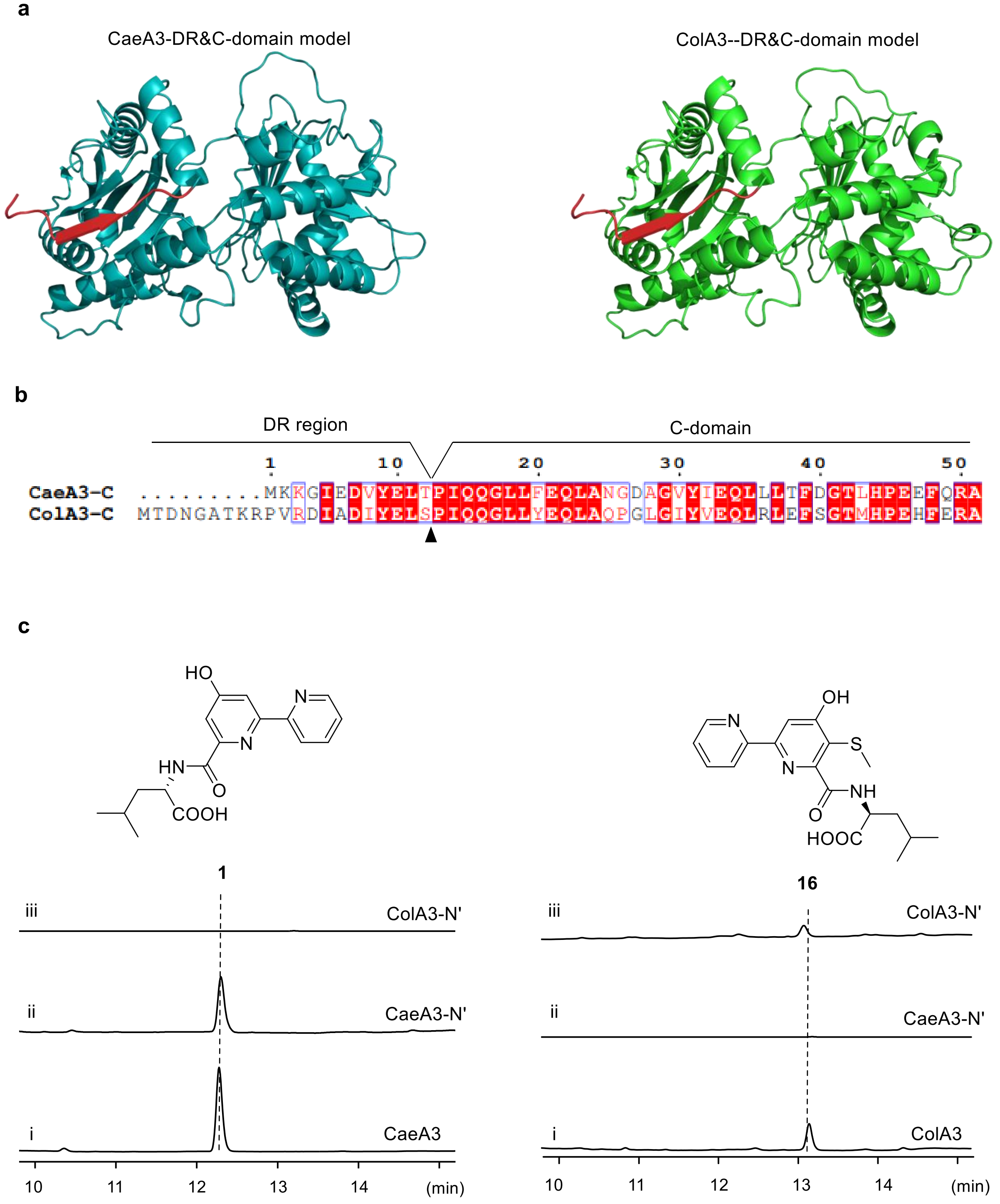

Supplementary Figure 21. Analysis of the docking regions (DRs) of CaeA3 and ColA3.

(**a**) Homology modeling of the N-terminal DRs and C-domains of CaeA3 (left) and ColA3 (right) based on the first ranked structural homolog, the SrfA C-domain (PDB: 2VSQ), from *Bacillus subtilis* by SWISS-MODEL tools. Secondary structure prediction revealed the N-terminal DRs of CaeA3 and

ColA3 that vary in size, share high homology to each other and feature a ß-sheet-containing sequence (red).

(**b**) Sequence alignment. The black triangle indicates the border between the N-terminal DR and the C-domain from CaeA3 or ColA3.

(**c**) *In vitro* assays for the exchangeability of DRs. Tested reactions were derived from the combination of CaeA1, CaeA2 and CaeB1 with picolinic acid, malonyl-*S*-CoA, L-cysteine, L-leucine, and ATP. For **1** production (left), CaeA3 (i), CaeA3-N' (ii) and ColA3-N' were incorporated, respectively. for **16** production (right), ColA3(i), CaeA3-N' (ii) and ColA3-N' were incorporated, respectively, along with ColG2 and SAM.

SUPPLEMENTARY TABLES

Supplementary Table 1. Bacterial strains in this study.

Strain/plasmid	Characteristics	Source
E. coli		
DH5α	Host for general cloning	Invitrogen
S17-1	Donor strain for conjugation between *E. coli* and *Streptomyces*	[2]
BL21 (DE3)	Host for protein expression	NEB
BAP1	Host for protein expression, contains *sfp* gene under the control of the T7 RNA polymerase promoter	[44]
S. raseosporus		
NRRL 11379	COL-producing wild type strain	NRRL
QL2003	*colA2* inframe deletion mutant, COL non-producing	[7]
QL2006	QL2003 derivative, in which chimeric gene *col/caeA2* was expressed *in trans*	This study
A. cyanogriseus		
NRRL B-2194	CAE-producing wild type strain	NRRL

Supplementary Table 2. Plasmids in this study.

**Plasmids**	Characteristics	Source
pMD19-T	*E. coli* subcloning vector, ampicillin resistance	Takara
pSET152	*E. coli-Streptomyces* shuttle vector for gene complementation, apramycin resistance	2
pET-28a(+)	Protein expression vector used in *E.coli*, encoding N-terminal 6 × His-tag, kanamycin resistance	Novagen
pET-37b(+)	Protein expression vector used in *E.coli*, encoding C-terminal 8 × His-tag, kanamycin resistance	Novagen
pSJ5	Protein expression vector used in *E.coli*, encoding N-terminal $His_{\times 6}$-Trx-tag, ampicillin resistance	Transformed from pET32a, adding Trx-tag and after N-terminal His
pQ8	Protein expression vector used in *E.coli*, encoding N-terminal $His_{\times 6}$-MBP-$His_{\times 6}$-tag, kanamycin resistance	45
pQL1022	pSET152 derivative containing the 8.0 kb *ermE** promoter + *colA2* gene	7
pQL1026	pMD19-T derivative, containing *caeA1*	This study
pQL1027	pET-28a(+) derivative, containing *caeA1*	This study
pQL1028	pMD19-T derivative, containing *caeA2*	This study
pQL1029	pET-37b(+) derivative, containing *caeA2*	This study
pQL1030	pMD19-T derivative, containing *caeA3*	This study
PQL1031	pET-28a(+) derivative, containing *caeA3*	This study
pQL1032	pMD19-T derivative, containing *caeB1*	This study
pQL1033	pET-28a(+) derivative, containing *caeB1*	This study
pQL1034	pMD19-T derivative, containing *colB1*	This study
PQL1035	pET-28a(+) derivative, containing *colB1*	This study
pQL1036	pMD19-T derivative, containing *colA3*	This study

pQL1037	pET-28a(+) derivative, containing *colA3*	This study
pQL1038	pMD19-T derivative, containing *caeA4*	This study
pQL1039	pET-28a(+) derivative, containing *ceaA4*	This study
pQL1040	pMD19-T derivative, containing *caeA2-ΔCt*	This study
PQL1041	pET-28a(+) derivative, containing *caeA2-ΔCt*	This study
pQL1042	pMD19-T derivative, containing *colG2*	This study
pQL1043	pET-28a(+) derivative, containing *colG2*	This study
pQL1044	pMD19-T derivative, containing the gene encoding the PCP domain of CaeA2	This study
pQL1045	pET-28a(+) derivative, containing the gene encoding the PCP domain of CaeA2	This study
pQL1046	pMD19-T derivative, the gene encoding the C domain of CaeA3	This study
pQL1047	pMD19-T derivative, containing the gene encoding the A-PCP didomain of ColA3	This study
pQL1048	pET-28a(+) derivative, containing the gene encoding chimeric NRPS Cae/ColA3	This study
pQL1049	pET-37b(+) derivative, containing the gene encoding CaeA2-F2042L	This study
pQL1050	pET-37b(+) derivative, containing the gene encoding CaeA2-F2042I	This study
pQL1051	pET-37b(+) derivative, containing the gene encoding CaeA2-F2042V	This study
pQL1052	pET-37b(+) derivative, containing the gene encoding CaeA2-F2042L-Δ Ct	
pQL1053	pMD19-T derivative, containing the gene encoding the NRPS module of CaeA2	This study

pQL1054	pQL1022 derivative, containing the chimeric gene *col/caeA2* coding for the hybrid protein that harbors the PKS module from ColA2 and the NRPS module from CaeA2	This study
pQL1055	pSJ5 derivative, containing the gene encoding the Ct domain of CaeA2	This study
pQL1056	pQ8 derivative, containing the gene encoding CeaB1	This study
pQL1057	pET-28a(+) derivative, containing the gene encoding the docking region of ColA3 and the core region of CaeA3 (named *caeA3-N'*)	This study
pQL1058	pET-28a(+) derivative, containing the gene encoding the docking region of CaeA3 and the core region of ColA3 (named *colA3-N'*)	This study
pQL1059	pET-28a(+) derivative, containing the gene encoding Sumo-tagged PCP-Ct$_{CaeA2}$	This study

Supplementary Table 3. Primers used in this study. The sequences of restriction enzymes are underlined.

Primer	Sequence
CaeA1-For	5'-GCGCGAATTCCATATGGAAGAACTGCGAAGGATT-3' (NdeI)
CaeA1-Rev	5'-ATATAAGCTTACTCGAGGGTTCGGGAAGCGTCC-3' (HindIII)
CaeA2-For	5'-AGCTGAATTCCATATGACCGGTCATGACACCGT-3' (NdeI)
CaeA2-Rev	5'-AGCTAAGCTTACTCGAGCTTCTCAAGGGTGGACAGC-3' (XhoI)
CaeA3-For	5'-GCGCGAATTCCATATGAGCCAGTTGAAAAAGGGCATCG-3' (NdeI)
CaeA3-Rev	5'-ATATAAGCTTACTCGAGCGCCCGCTCCCTCTCCAC-3' (HindIII)

CaeA4-For	5’-GCGCGAATTCCATATG TTCAGGTCGTGGTCGGCGG-3’(NdeI)
CaeA4-Rev	5’-ATATAAGCTTTCAGGGGGACGACCGCTGCGGCG-3’ (HindIII)
CaeB1-For	5’-ATGCGAATTCCATATGAGCGCACCGACGGTGACCAC-3’ (NdeI)
CaeB1-Rev	5’-ATGCAAGCTTACTCGAGCAGCGACCCCGCGATGCTCC-3’ (HindIII)
ColB1-For	5’-GCGCGAATTCCATATGAATACGAACCCCGGACAGAGC-3’ (NdeI)
ColB1-Rev	5’-ATATAAGCTTACTCGAGGTCACCGGGGCTCCCT-3’ (HindIII)
ColA3-For	5’-TTGAATTCCATATGGCGACAACGGCGGACGGGACT-3’ (NdeI)
ColA3-Rev	5’-TTAAGCTTACTCGAGCCGTGCCCCCTCCCCCGCGACC-3’ (HindIII)
CaeA2-ΔCt-For	5’-TTGAATTCCATATGACCGGTCATGACACCGTCGAGTC-3’ (NdeI)
CaeA2-ΔCt-Rev	5’-TTAAGCTTACTCGAGGGCCGCCTGGTGCGGGGTGAAC-3’ (XhoI)
ColG2-For	5’-TTGAATTCCATATGAGCGGCGCGATATCGACCGAG-3’ (NdeI)
ColG2-Rev	5’-TTAAGCTTCTACCCCGGTACGACGGTCTCGA-3’ (HindIII)
CaeA2-PCP-For	5’-TTGAATTCCATATGCCCGCGCCGGACCGCGTCGG-3’ (NdeI)
CaeA2-PCP-Rev	5’-TTAAGCTTACTCGAGCGCGACCGTCCCGTCCGCCTC-3’ (HindIII)
CaeA3-C-For	5’-TTGAATTCCATATGAAAAAGGGCATCGAGGATGTCTACGAACTCAC-3’ (NdeI)
CaeA3-C-Rev	5’-TTGCTAGCGTCGGGGTTGGCGGCGGCCAGGTC-3’ (NheI)
ColA3-APCP-For	5’-TTGCTAGCGCCGTGCGCTGCGGAGGCCG-3’ (NheI)
ColA3-APCP-Rev	5’-TTAAGCTTACTCGAGCCGTGCCCCCTCCCCCGCGAC-3’ (HindIII)
CaeA2-F2042L-For	5’-TGGGTATCCAGTTGGTCAGCCGTGCCG-3’

CaeA2-F2042L-Rev	5’-GGCTGACCAACTGGATACCCATGATCGA-3’
CaeA2-F2042I- For	5’-TGGGTATCCAGATCGTCAGCCGTGCCG-3’
CaeA2-F2042I-Rev	5’-GGCTGACGATCTGGATACCCATGATCGA-3’
CaeA2-F2042V-For	5’-TGGGTATCCAGGTCGTCAGCCGTGCCG-3’
CaeA2-F2042V-Rev	5’-GGCTGACGACCTGGATACCCATGATCGA-3’
CaeA3-Core-For	5’-CTCACCCCGATCCAGCAGGGCCTGCTGT-3’
CaeA3-Core-Rev	5’-GTGGTGGTGCTCGAGTCACGCCCGCTCCCTC-3’
ColA3-DR-For	5’-CTCGAGCACCACCACCACCACCACTGAGATC-3’
ColA3-DR-Rev	5’-GATCGGGGTGAGTTCGTAGATGTCCGCGATGT-3’
ColA3-Core-For	5’GCGCGGCAGCCATATGAGCCAGTTGAAAAAGGGCATCGAGG ATGTCTACGAACTCTCGCCGATCC-3’
ColA3-Core-Rev	5’-TGGTGCTCGAGTCACCGTGCCCCCTCC-3’
CaeA2-PCP-Ct-For	5’-TGGTGGATCC<u>GAATTC</u>ATGGAGCGGCTGCTCGCCCT-3’
CaeA2-PCP-Ct-Rev	5’-TGCGGCCGC<u>AAGCTT</u>TCACTTCTCAAGGGTG-3’
Cae-CO-for	5’-TT<u>AGATCT</u>CAGCCGTTCCCGCTGAACGAGAT-3’ (BglII)
Cae-Ct-rev	5’-TT<u>GATATC</u>TTACTTCTCAAGGGTGGACAGCAGGGC-3’ (EcoRV)

Supplementary Table 4. MS/MS data for PCP-aminoacylation on CaeA2.

Detail MS/MS data for **Supplementary Figure 5A**

	Calcd *m/z*	Obsd *m/z*	Error
b2	201.1239	201.1220	9 ppm
y3	361.2198	361.2183	4 ppm
y4	508.2882	508.2856	5 ppm
y5	636.3467	636.3420	7 ppm
y7	806.4521	806.4467	6 ppm
y8	937.4925	937.4888	4 ppm
y9	1050.576	1050.5739	2 ppm
Ppant ejection ion	261.1267	261.1252	5 ppm

Detail MS/MS data for **Supplementary Figure 5B**

	Calcd *m/z*	Obsd *m/z*	Error
b2	201.1239	201.1220	9 ppm
y3	361.2198	361.2178	5 ppm
y4	474.3038	474.3040	0.5 ppm
y5	602.3623	602.3628	1 ppm
y6	715.4463	715.4470	1 ppm
y7	772.4677	772.4686	1 ppm
y8	903.5081	903.5049	3 ppm
y9	1016.592	1016.5834	8 ppm
Ppant ejection ion	261.1267	261.1269	1 ppm

Detail MS/MS data for **Supplementary Figure 6A**

	Calcd *m/z*	Obsd *m/z*	Error
b2	201.1239	201.1233	3 ppm
y2	262.1514	262.1507	3 ppm
y4	474.3038	474.3027	2 ppm
y5	602.3623	602.3621	3 ppm
y6	715.4463	715.4460	4 ppm
y7	772.4677	772.4669	1 ppm
y8	903.5081	903.5062	2 ppm
y9	1016.592	1016.5920	0 ppm
Ppant ejection ion	364.1359	364.1356	1 ppm

Detail MS/MS data for **Supplementary Figure 6B**

	Calcd *m/z*	Obsd *m/z*	Error
b2	201.1239	201.1225	7 ppm
b4	430.1936	430.1879	13 ppm
y3	361.2198	361.2177	6 ppm
y4	474.3038	474.3012	5 ppm
y5	602.3623	602.3595	5 ppm
y6	715.4463	715.4433	4 ppm
y7	772.4677	772.4642	4 ppm
y8	903.5081	903.5040	4 ppm
y9	1016.592	1016.5881	4 ppm
Ppant ejection ion	421.1574	421.1555	4 ppm

Detail MS/MS data for **Supplementary Figure 7A**, **i**

	Calcd *m/z*	Obsd *m/z*	Error
y3	361.2198	361.2189	2 ppm
y4	474.3038	474.3031	1 ppm
y5	602.3623	602.3622	0.2 ppm
y6	715.4463	715.4452	1 ppm
y7	772.4677	772.4666	1 ppm
y8	903.5081	903.5055	3 ppm
y9	1016.592	1016.5910	1 ppm
Ppant ejection ion	421.1574	421.1570	1 ppm

Detail MS/MS data for **Supplementary Figure 7A**, **ii**

	Calcd *m/z*	Obsd *m/z*	Error
b2	201.1239	201.1225	7 ppm
b3	258.1453	258.1435	7 ppm
y3	361.2198	361.2168	8 ppm
y4	474.3038	474.3021	3 ppm
y5	602.3623	602.3593	5 ppm
y6	715.4463	715.4423	5 ppm
y7	772.4677	772.4636	5 ppm
y8	903.5081	903.5048	4 ppm
y9	1016.592	1016.6003	8 ppm
Ppant ejection ion	420.1257	420.1234	5 ppm

Detail MS/MS data for **Supplementary Figure 7B**, **i**

	Calcd *m/z*	Obsd *m/z*	Error
b2	201.1239	201.1227	6 ppm
b3	315.1667	315.1646	7 ppm
y3	361.2198	361.2180	5 ppm
y4	474.3038	474.3016	4 ppm
y5	602.3623	602.3599	4 ppm
y6	715.4463	715.4435	4 ppm
y7	772.4677	772.4647	4 ppm
y8	903.5081	903.5048	4 ppm
y9	1016.592	1016.5881	4 ppm
Ppant ejection ion	425.1646	425.1629	4 ppm

Detail MS/MS data for **Supplementary Figure 7B**, **ii**

	Calcd *m/z*	Obsd *m/z*	Error
b2	201.1239	201.1226	6 ppm
b3	315.1667	315.1642	8 ppm
y3	361.2198	361.2181	5 ppm
y4	474.3038	474.3016	5 ppm
y5	602.3623	602.3597	4 ppm
y6	715.4463	715.4440	3 ppm
y7	772.4677	772.4651	3 ppm
y8	903.5081	903.5044	4 ppm
y9	1016.592	1016.5879	4 ppm
Ppant ejection ion	423.1360	423.1341	4 ppm

Detail MS/MS data for **Supplementary Figure 7C**, **i**

	Calcd *m/z*	Obsd *m/z*	Error
b2	201.1239	201.1227	6 ppm
y3	361.2198	361.2181	5 ppm
y4	474.3038	474.3016	5 ppm
y5	602.3623	602.3602	3 ppm
y6	715.4463	715.4440	3 ppm
y7	772.4677	772.4651	3 ppm
y8	903.5081	903.5044	4 ppm
y9	1016.592	1016.5883	3 ppm
Ppant ejection ion	424.1762	424.1747	3 ppm

Detail MS/MS data for **Supplementary Figure 7C**, **ii**

	Calcd *m/z*	Obsd *m/z*	Error
b2	201.1239	201.1227	6 ppm
b5	430.1936	430.2017	18 ppm
y3	361.2198	361.2181	5 ppm
y4	474.3038	474.3021	3 ppm
y5	602.3623	602.3597	4 ppm
y6	715.4463	715.4438	3 ppm
y7	772.4677	772.4651	3 ppm
y8	903.5081	903.5052	3 ppm
y9	1016.592	1016.5890	3 ppm
Ppant ejection ion	420.1257	420.1244	3 ppm

Detail MS/MS data for **Supplementary Figure 10A**

	Calcd *m/z*	Obsd *m/z*	Error
b2	201.1239	201.1230	5 ppm
b5	430.1936	430.2122	40 ppm
y3	361.2198	361.2185	4 ppm
y4	474.3038	474.3023	3 ppm
y5	602.3623	602.3608	2 ppm
y6	715.4463	715.4449	2 ppm
y7	772.4677	772.5064	50 ppm
y8	903.5081	903.5052	3 ppm
y9	1016.592	1016.5903	2 ppm
Ppant ejection ion	421.1574	421.1565	2 ppm

Detail MS/MS data for **Supplementary Figure 10B**

	Calcd *m/z*	Obsd *m/z*	Error
b2	201.1239	201.1231	4 ppm
y1	175.1194	175.1187	4 ppm
y3	361.2198	361.2185	3 ppm
y4	474.3038	474.3025	2 ppm
y5	602.3623	602.3610	2 ppm
y6	715.4463	715.4457	1 ppm
y7	772.4677	772.4665	1 ppm
y8	903.5081	903.5069	1 ppm
y9	1016.592	1016.5907	1 ppm
Ppant ejection ion	421.1574	421.1566	2 ppm

Detail MS/MS data for **Supplementary Figure 13A**

	Calcd *m/z*	Obsd *m/z*	Error
b2	201.1239	201.1226	6 ppm
b4	315.1667	315.1654	4 ppm
y3	361.2198	361.2197	0.5 ppm
y4	474.3038	474.3040	0.5 ppm
y5	602.3623	602.3593	5 ppm
y6	715.4463	715.4444	3 ppm
y7	772.4677	772.4638	5 ppm
y8	903.5081	903.5040	5 ppm
y9	1016.592	1016.5868	5 ppm
Ppant ejection ion	459.1697	459.1675	5 ppm

Detail MS/MS data for **Supplementary Figure 13B**

	Calcd *m/z*	Obsd *m/z*	Error
b2	201.1239	201.1233	3 ppm
Y2	262.1514	262.1513	0.5 ppm
y3	361.2198	361.2206	2 ppm
y4	474.3038	474.3079	9 ppm
y5	602.3623	602.3608	2 ppm
y6	715.4463	715.4468	1 ppm
y7	772.4677	772.4681	0.5 ppm
y8	903.5081	903.5107	3 ppm
y9	1016.592	1016.5889	3 ppm
Ppant ejection ion	459.1697	459.1704	2 ppm

SUPPLEMENTARY REFERENCES

1 Green, M. R. & Sambrook, J. *Molecular Cloning: A Laboratory Manual* (Cold Spring Harbor Laboratory Press, 2012).

2 Kieser, T., Bibb, M. J., Buttner, M. J., Chater, K. F. & Hopwood, D. A. *Practical Streptomyces Genetics*. (John Innes Foundation, 2000).

3 Li, D. *et al.* pFind: a novel database-searching software system for automated peptide and protein identification via tandem mass spectrometry. *Bioinformatics* **21**, 3049-3050 (2005).

4 Chen, M., Pang, B., Du, Y. N., Zhang, Y. P. & Liu, W. Characterization of the metallo-dependent amidohydrolases responsible for "auxiliary" leucinyl removal in the biosynthesis of 2,2'-bipyridine antibiotics. *Synth Syst Biotechnol* **2**, 137-146 (2017).

5 Wei, C. *et al.* Dual-Reactable Fluorescent Probes for Highly Selective and Sensitive Detection of Biological H_2S. *Chem. Asian J.* **11**, 1376-1381 (2016).

6 Bumpus, S. B. & Kelleher, N. L. Accessing natural product biosynthetic processes by mass spectrometry. *Curr. Opin. Chem. Biol.* **12**, 475-482 (2008).

7 Qu, X. *et al.* Caerulomycins and collismycins share a common paradigm for 2,2'-bipyridine biosynthesis via an unusual hybrid polyketide-peptide assembly Logic. *J. Am. Chem. Soc.* **134**, 9038-9041 (2012).

8 Watanabe, K., Khosla, C., Stroud, R. M. & Tsai, S.-C. Crystal Structure of an Acyl-ACP Dehydrogenase from the FK520 Polyketide Biosynthetic Pathway: Insights into Extender Unit Biosynthesis. *J. Mol. Biol.* **334**, 435-444 (2003).

9 Toogood, H. S. *et al.* Extensive domain motion and electron transfer in the human electron transferring flavoprotein.medium chain Acyl-CoA dehydrogenase complex. *J. Biol. Chem.* **279**, 32904-32912 (2004).

10 Battaile, K. P. *et al.* Crystal structure of rat short chain acyl-CoA dehydrogenase complexed with acetoacetyl-CoA: comparison with other acyl-CoA dehydrogenases. *J. Biol. Chem.* **277**, 12200-12207 (2002).

11 Kung, J. W., Seifert, J., von Bergen, M. & Boll, M. Cyclohexanecarboxyl-coenzyme A (CoA) and cyclohex-1-ene-1-carboxyl-CoA dehydrogenases, two enzymes involved in the fermentation of benzoate and crotonate in Syntrophus aciditrophicus. *J. Bacteriol.* **195**, 3193-3200 (2013).

12 Mootz, H. D. & Marahiel, M. A. The tyrocidine biosynthesis operon of Bacillus brevis: complete nucleotide sequence and biochemical characterization of functional internal adenylation domains. *J. Bacteriol.* **179**, 6843-6850 (1997).

13 Konz, D., Klens, A., Schörgendorfer, K. & Marahiel, M. A. The bacitracin biosynthesis operon of Bacillus licheniformis ATCC 10716: molecular characterization of three multi-modular peptide synthetases. *Chem. Biol.* **4**, 927-937 (1997).

14 Duitman, E. H. *et al.* The mycosubtilin synthetase of Bacillus subtilis ATCC6633: a multifunctional hybrid between a peptide synthetase, an amino transferase, and a fatty acid synthase. *Proc. Natl. Acad. Sci. U. S. A.* **96**, 13294-13299 (1999).

15 Molnár, I. *et al.* The biosynthetic gene cluster for the microtubule-stabilizing agents epothilones A and B from Sorangium cellulosum So ce90. *Chem. Biol.* **7**, 97-109 (2000).

16 Gaudelli, N. M., Long, D. H. & Townsend, C. A. beta-Lactam formation by a non-ribosomal

peptide synthetase during antibiotic biosynthesis. *Nature* **520**, 383-387 (2015).
17 Hojati, Z. *et al.* Structure, Biosynthetic Origin, and Engineered Biosynthesis of Calcium-Dependent Antibiotics from Streptomyces coelicolor. *Chem. Biol.* **9**, 1175-1187 (2002).
18 Chiu, H. T. *et al.* Molecular cloning and sequence analysis of the complestatin biosynthetic gene cluster. *Proc. Natl. Acad. Sci. U. S. A.* **98**, 8548-8553 (2001).
19 Steller, S. *et al.* Structural and functional organization of the fengycin synthetase multienzyme system from Bacillus subtilis b213 and A1/3. *Chem. Biol.* **6**, 31-41 (1999).
20 Turgay, K., Krause, M. & Marahiel, M. A. Four homologous domains in the primary structure of GrsB are related to domains in a superfamily of adenylate-forming enzymes. *Mol. Microbiol.* **6**, 529-546 (1992).
21 Tsuge, K., Akiyama, T. & Shoda, M. Cloning, sequencing, and characterization of the iturin A operon. *J. Bacteriol.* **183**, 6265-6273 (2001).
22 Konz, D., Doekel, S. & Marahiel, M. A. Molecular and biochemical characterization of the protein template controlling biosynthesis of the lipopeptide lichenysin. *J. Bacteriol.* **181**, 133-140 (1999).
23 Bamas-Jacques, N., Lorenzon, S., Lacroix, P., de Swetschin, C. & Crouzet, J. Cluster organization of the genes of Streptomyces pristinaespiralis involved in pristinamycin biosynthesis and resistance elucidated by pulsed-field gel electrophoresis. *J. Appl. Microbiol.* **87**, 939-948 (1999).
24 Mast, Y. *et al.* Characterization of the 'pristinamycin supercluster' of Streptomyces pristinaespiralis. *Microb Biotechnol* **4**, 192-206 (2011).
25 Ziemert, N. *et al.* The natural product domain seeker NaPDoS: a phylogeny based bioinformatic tool to classify secondary metabolite gene diversity. *PLoS One* **7**, e34064 (2012).
26 Rausch, C., Hoof, I., Weber, T., Wohlleben, W. & Huson, D. H. Phylogenetic analysis of condensation domains in NRPS sheds light on their functional evolution. *BMC Evol. Biol.* **7**, 78-92 (2007).
27 Schauwecker, F., Pfennig, F., Schroder, W. & Keller, U. Molecular cloning of the actinomycin synthetase gene cluster from Streptomyces chrysomallus and functional heterologous expression of the gene encoding actinomycin synthetase II. *J. Bacteriol.* **180**, 2468-2474 (1998).
28 Roongsawang, N. *et al.* Cloning and characterization of the gene cluster encoding arthrofactin synthetase from Pseudomonas sp MIS38. *Chem. Biol.* **10**, 869-880 (2003).
29 Pogorevc, D. *et al.* Biosynthesis and Heterologous Production of Argyrins. *Acs Synth Biol* **8**, 1121-1133 (2019).
30 Yuwen, L. *et al.* The role of aromatic L-amino acid decarboxylase in bacillamide C biosynthesis by Bacillus atrophaeus C89. *Sci. Rep.* **3**, 1753-1762 (2013).
31 Du, L., Sánchez, C., Chen, M., Edwards, D. J. & Shen, B. The biosynthetic gene cluster for the antitumor drug bleomycin from Streptomyces verticillus ATCC15003 supporting functional interactions between nonribosomal peptide synthetases and a polyketide synthase. *Chem. Biol.* **7**, 623-642 (2000).
32 Homburg, S., Oswald, E., Hacker, J. & Dobrindt, U. Expression analysis of the colibactin gene cluster coding for a novel polyketide in Escherichia coli. *FEMS Microbiol. Lett.* **275**, 255-262 (2007).
33 Gehring, A. M., Mori, I. & Walsh, C. T. Reconstitution and characterization of the Escherichia coli enterobactin synthetase from EntB, EntE, and EntF. *Biochemistry* **37**, 2648-2659 (1998).

34 Gehring, A. M. *et al.* Iron acquisition in plague: modular logic in enzymatic biogenesis of yersiniabactin by Yersinia pestis. *Chem. Biol.* **5**, 573-586 (1998).
35 Nishizawa, T. *et al.* Polyketide synthase gene coupled to the peptide synthetase module involved in the biosynthesis of the cyclic heptapeptide microcystin. *J. Biochem.* **127**, 779-789 (2000).
36 Nowak-Thompson, B., Chaney, N., Wing, J. S., Gould, S. J. & Loper, J. E. Characterization of the pyoluteorin biosynthetic gene cluster of Pseudomonas fluorescens Pf-5. *J. Bacteriol.* **181**, 2166-2174 (1999).
37 Chen, J.-S. *et al.* Functional identification of the gene encoding the enzyme involved in mannosylation in ramoplanin biosynthesis in Actinoplanes sp. *Biotechnol. Lett* **35**, 1501-1508 (2013).
38 Bruner, S. D. *et al.* Structural basis for the cyclization of the lipopeptide antibiotic surfactin by the thioesterase domain SrfTE. *Structure* **10**, 301-310 (2002).
39 Scholz-Schroeder, B. K., Soule, J. D. & Gross, D. C. The sypA, sypB and sypC synthetase genes encode twenty-two modules involved in the nonribosomal peptide synthesis of syringopeptin by Pseudomonas syringae pv. syringae B301D. *Mol. Plant. Microbe Interact.* **16**, 271-280 (2003).
40 Pootoolal, J. *et al.* Assembling the glycopeptide antibiotic scaffold: The biosynthesis of A47934 from Streptomyces toyocaensis NRRL 15009. *Proc. Natl. Acad. Sci. U. S. A.* **99**, 8962-8967 (2002).
41 Guenzi, E., Galli, G., Grgurina, I., Gross, D. C. & Grandi, G. Characterization of the syringomycin synthetase gene cluster - A link between prokaryotic and eukaryotic peptide synthetases. *J. Biol. Chem.* **273**, 32857-32863 (1998).
42 Lombo, F. *et al.* Deciphering the biosynthesis pathway of the antitumor thiocoraline from a marine actinomycete and its expression in two Streptomyces species. *Chembiochem* **7**, 366-376 (2006).
43 Wyckoff, E. E., Stoebner, J. A., Reed, K. E. & Payne, S. M. Cloning of a Vibrio cholerae vibriobactin gene cluster: Identification of genes required for early steps in siderophore biosynthesis. *J. Bacteriol.* **179**, 7055-7062 (1997).
44 Pfeifer, B. A., Admiraal, S. J., Gramajo, H., Cane, D. E. & Khosla, C. Biosynthesis of complex polyketides in a metabolically engineered strain of E. coli. *Science* **291**, 1790-1792 (2001).
45 Fang, J. *et al.* Cloning and characterization of the tetrocarcin A gene cluster from Micromonospora chalcea NRRL 11289 reveals a highly conserved strategy for tetronate biosynthesis in spirotetronate antibiotics. *J. Bacteriol.* **190**, 6014-6025 (2008).
46 Garcia-Salcedo, R. *et al.* Characterization of the Jomthonic Acids Biosynthesis Pathway and Isolation of Novel Analogues in Streptomyces caniferus GUA-06-05-006A. *Mar. Drugs* **16** (2018).
47 Galm, U. *et al.* The biosynthetic gene cluster of zorbamycin, a member of the bleomycin family of antitumor antibiotics, from Streptomyces flavoviridis ATCC 21892. *Mol Biosyst* **5**, 77-90 (2009).
