# Supplementary material for: Caerulomycin and collismycin antibiotics share a trans-acting flavoprotein-dependent assembly line for 2,2’-bipyridine formation
Source: Nat Commun. 2021 May 25;12:3124. doi: 10.1038/s41467-021-23475-4 (PMC8149447; doi:10.1038/s41467-021-23475-4)
Supplement: Supplementary file 3 — Reporting Summary [file 41467_2021_23475_MOESM3_ESM.pdf]

nature research

Corresponding author(s): Wen Liu

Last updated by author(s): April 15, 2021

# Reporting Summary

Nature Research wishes to improve the reproducibility of the work that we publish. This form provides structure for consistency and transparency in reporting. For further information on Nature Research policies, see our Editorial Policies and the Editorial Policy Checklist.

## Statistics

For all statistical analyses, confirm that the following items are present in the figure legend, table legend, main text, or Methods section.

| n/a | Confirmed | |
|---|---|---|
| ☐ | ☒ | The exact sample size (*n*) for each experimental group/condition, given as a discrete number and unit of measurement |
| ☐ | ☒ | A statement on whether measurements were taken from distinct samples or whether the same sample was measured repeatedly |
| ☒ | ☐ | The statistical test(s) used AND whether they are one- or two-sided<br>*Only common tests should be described solely by name; describe more complex techniques in the Methods section.* |
| ☒ | ☐ | A description of all covariates tested |
| ☒ | ☐ | A description of any assumptions or corrections, such as tests of normality and adjustment for multiple comparisons |
| ☒ | ☐ | A full description of the statistical parameters including central tendency (e.g. means) or other basic estimates (e.g. regression coefficient) AND variation (e.g. standard deviation) or associated estimates of uncertainty (e.g. confidence intervals) |
| ☒ | ☐ | For null hypothesis testing, the test statistic (e.g. *F*, *t*, *r*) with confidence intervals, effect sizes, degrees of freedom and *P* value noted<br>*Give P values as exact values whenever suitable.* |
| ☒ | ☐ | For Bayesian analysis, information on the choice of priors and Markov chain Monte Carlo settings |
| ☒ | ☐ | For hierarchical and complex designs, identification of the appropriate level for tests and full reporting of outcomes |
| ☒ | ☐ | Estimates of effect sizes (e.g. Cohen's *d*, Pearson's *r*), indicating how they were calculated |

*Our web collection on statistics for biologists contains articles on many of the points above.*

## Software and code

Policy information about availability of computer code

| | |
|---|---|
| Data collection | HPLC-ESI-MS and MS/MS data for small molecules were recorded on a Thermo Fisher LTQ Fleet ESI-MS spectrometer (Thermo Fisher Scientific Inc., USA). NanoLC-MS/MS and MS/MS for peptides were performed on an EASY-nLC 1200 (Thermo Fisher Scientific Inc., USA) coupled with a Q Exactive HF mass spectrometer (Thermo Fisher Scientific Inc., USA). High resolution ESI-MS (HR-ESI-MS) analysis for small molecules was carried out on an Agilent 6230B Accurate Mass TOF LC/MS System (Agilent Technologies Inc., USA). NMR data were recorded on an Agilent 500 MHz PremiumCompact+ NMR spectrometer (Agilent Technologies Inc., USA). ITC date were recorded on MicroCal-ITC200 (Malvern). |
| Data analysis | Open reading frames (ORFs) were identified using the FramePlot 4.0beta program (http://nocardia.nih.go.jp/fp4/). The deduced proteins were compared with other known proteins in the databases using available BLAST methods (http://blast.ncbi.nlm.nih.gov/Blast.cgi). Amino acid sequence alignments were performed using Vector NT1 and ESPript 3.0 (http://espript.ibcp.fr/ESPript/ESPript/). To probe peptide-tethered intermediates, all MS/MS data were transformed into .pymzML format file by MSConvert and then analyzed using script written by Python. Data obtained from LTQ Fleet ESI-MS spectrometer and Q Exactive HF mass spectrometer were analyzed by Thermo Xcalibur 2.0 software. Data obtained from Agilent 6230B Accurate Mass TOF were analyzed by Agilent MassHunter Qualitative Analysis B.06.00 software. NMR data were analyzed by MestReNova. ITC data were analyzed by MicroCal-PEAQ-ITC 1.21. |

For manuscripts utilizing custom algorithms or software that are central to the research but not yet described in published literature, software must be made available to editors and reviewers. We strongly encourage code deposition in a community repository (e.g. GitHub). See the Nature Research guidelines for submitting code & software for further information.

## Data

Policy information about availability of data

All manuscripts must include a data availability statement. This statement should provide the following information, where applicable:

- Accession codes, unique identifiers, or web links for publicly available datasets
- A list of figures that have associated raw data
- A description of any restrictions on data availability

The data underlying the findings of this study are available in this article and its Supplementary Information, or are available from the corresponding authors upon reasonable request.

# Field-specific reporting

Please select the one below that is the best fit for your research. If you are not sure, read the appropriate sections before making your selection.

[x] Life sciences  [ ] Behavioural & social sciences  [ ] Ecological, evolutionary & environmental sciences

For a reference copy of the document with all sections, see nature.com/documents/nr-reporting-summary-flat.pdf

# Life sciences study design

All studies must disclose on these points even when the disclosure is negative.

| | |
|---|---|
| Sample size | In this study, the HPLC and LC-MS analysis were performed >= 3 times |
| Data exclusions | No data were excluded from the analyses. |
| Replication | All experiments were repeated independently and showed similar results. Number of repeats is given in the figure legends. |
| Randomization | N/A. In this study, the data represented are the biochemical assay on enzyme function, which require a rational approach to data collection and analysis. |
| Blinding | N/A. This is an enzymatic study and appropriate control experiments were included. |

# Reporting for specific materials, systems and methods

We require information from authors about some types of materials, experimental systems and methods used in many studies. Here, indicate whether each material, system or method listed is relevant to your study. If you are not sure if a list item applies to your research, read the appropriate section before selecting a response.

## Materials & experimental systems

| n/a | Involved in the study | |
|---|---|---|
| [x] | [ ] | Antibodies |
| [x] | [ ] | Eukaryotic cell lines |
| [x] | [ ] | Palaeontology and archaeology |
| [x] | [ ] | Animals and other organisms |
| [x] | [ ] | Human research participants |
| [x] | [ ] | Clinical data |
| [x] | [ ] | Dual use research of concern |

## Methods

| n/a | Involved in the study | |
|---|---|---|
| [x] | [ ] | ChIP-seq |
| [x] | [ ] | Flow cytometry |
| [x] | [ ] | MRI-based neuroimaging |
